# Supplementary material for: Single‐cell profiling of muscle‐infiltrating T cells in idiopathic inflammatory myopathies
Source: EMBO Mol Med. 2023 Jul 31;15(10):e17240. doi: 10.15252/emmm.202217240 (PMC10565639; doi:10.15252/emmm.202217240)
Supplement: Supplementary file 1 — Appendix [file EMMM-15-e17240-s010.pdf]

Single-cell profiling of muscle-infiltrating T cells in idiopathic inflammatory myopathies

Alexandra Argyriou[1,2,6], Begum Horuluoglu[1,2,6], Angeles Shunashy Galindo-Feria[1,2,6], Juan Sebastian Diaz-Boada[1,2], Merel Sijbranda[1,2], Antonella Notarnicola[1,2,3], Lara Dani[1,3], Annika van Vollenhoven[1,2], Daniel Ramsköld[4], Inger Nennesmo[5], Maryam Dastmalchi[1,2,3], Ingrid E. Lundberg[1,2,3], Lina-Marcela Diaz-Gallo[1,2,7] and Karine Chemin[1,2,7]

[1]Division of Rheumatology, Department of Medicine, Solna, Karolinska Institutet, Stockholm, Sweden

[2]Center for Molecular Medicine, Karolinska Institutet, Stockholm, Sweden

[3]Department of Gastro, Dermatology and Rheumatology, Karolinska University Hospital, Stockholm, Sweden

[4]Department of Cell and Molecular Biology, Karolinska Institutet, Stockholm, Sweden

[5]Department of Pathology, Karolinska University Hospital, Stockholm, Sweden.

[6]co-first authors contributed equally to this manuscript, and each has the right to list themselves first in author order on their CVs

[7]equal senior contribution

Correspondence to:

Karine Chemin (karine.chemin@ki.se)

The authors declare that they have no conflict of interest.

TABLE OF CONTENT

Appendix Figure S1: Immune infiltrates in muscle biopsies from 6 patients with Idiopathic inflammatory myopathies and one patient with undefined myopathy

Appendix Figure S2: Flow cytometry gating strategy

Appendix Figure S3: Workflow representing the Smart-seq2 single-cell sequencing methodology

Appendix Table S1: Reagents

Appendix Table S2: Characteristics of additional patients with IIM from whom muscle biopsies were used for immunofluorescence and immunohistochemistry stainings

Appendix Figure S1

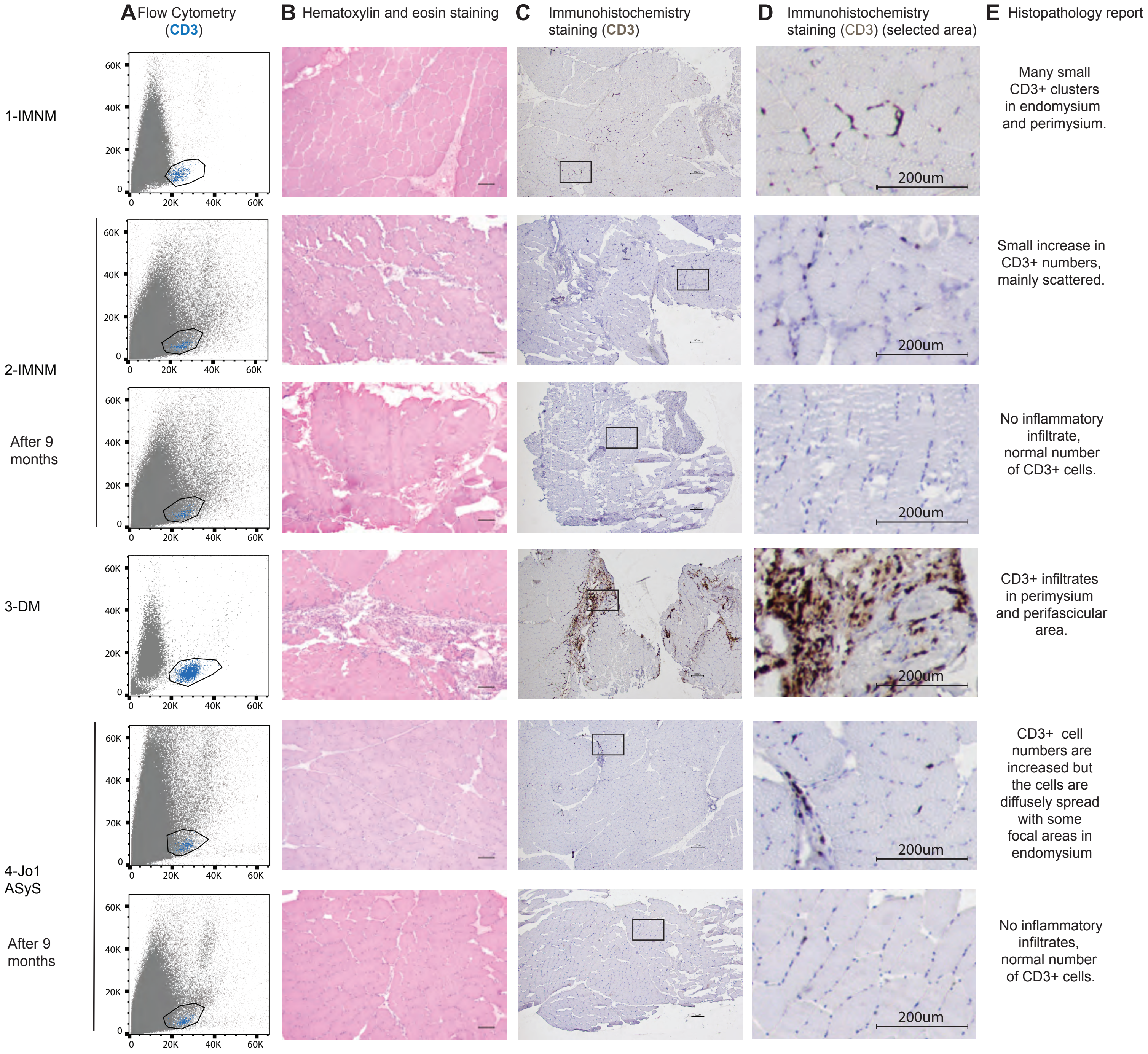

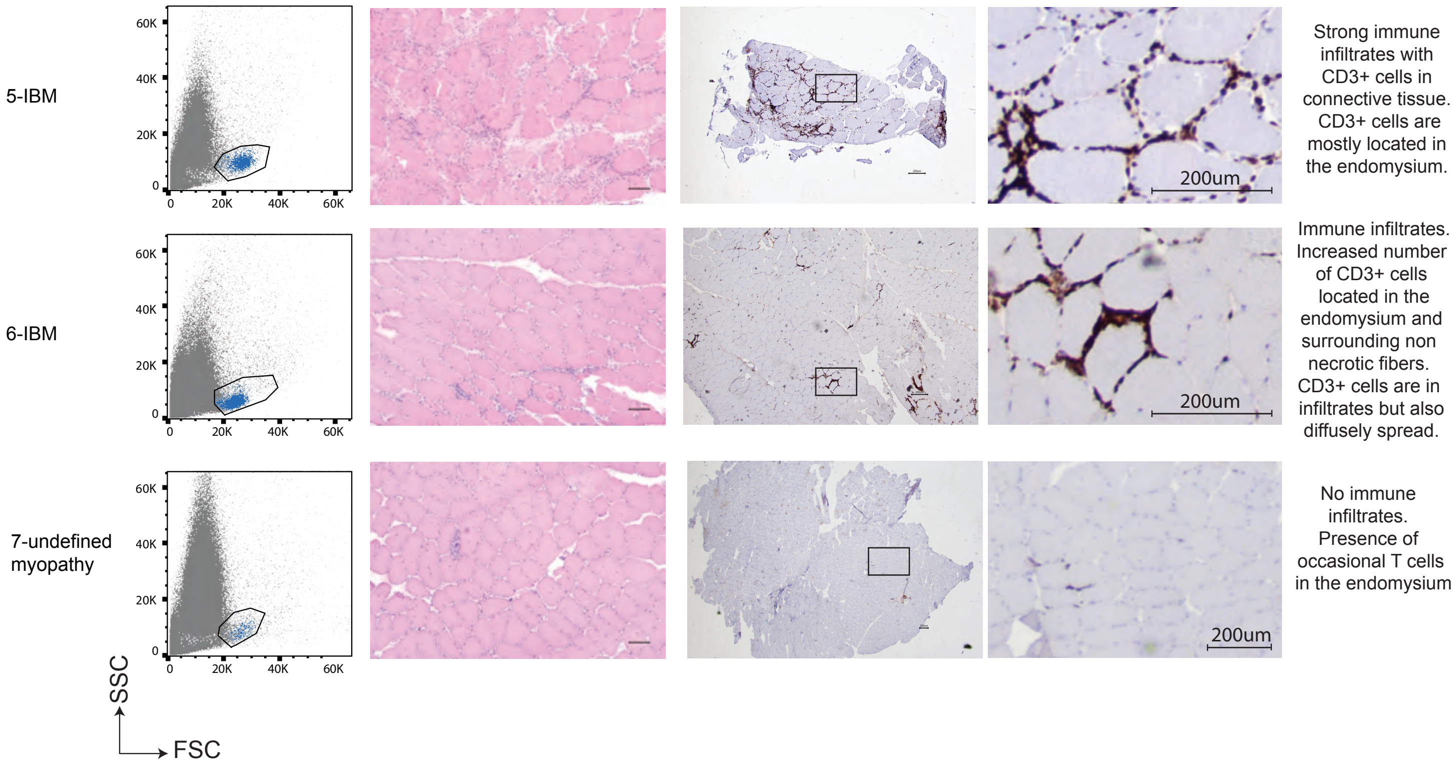

Appendix Figure S1. Immune infiltrates in muscle biopsies from six patients with myositis (1-6) and one patient with undefined myopathy (7). A) Flow cytometry plot showing CD45+CD3+ lymphocyte infiltrates (in blue) in muscle biopsies. **B)** Hematoxylin and eosin staining of muscle biopsies, scale bar=100um. **C)** Immunohistochemistry staining showing CD3+ lymphocytes (in brown), scale bar=200um **D)** selected square area from **C)** show examples of T cell infiltrates. **E)** Histopathology report. SSC: side scatter, FSC: forward side scatter. IMNM:Immune-Mediated Necrotizing Myopathy; DM: DermatoMyositis; ASyS:AntiSYnthetase Syndrome; IBM:Inclusion Body Myositis.

Appendix Figure S2

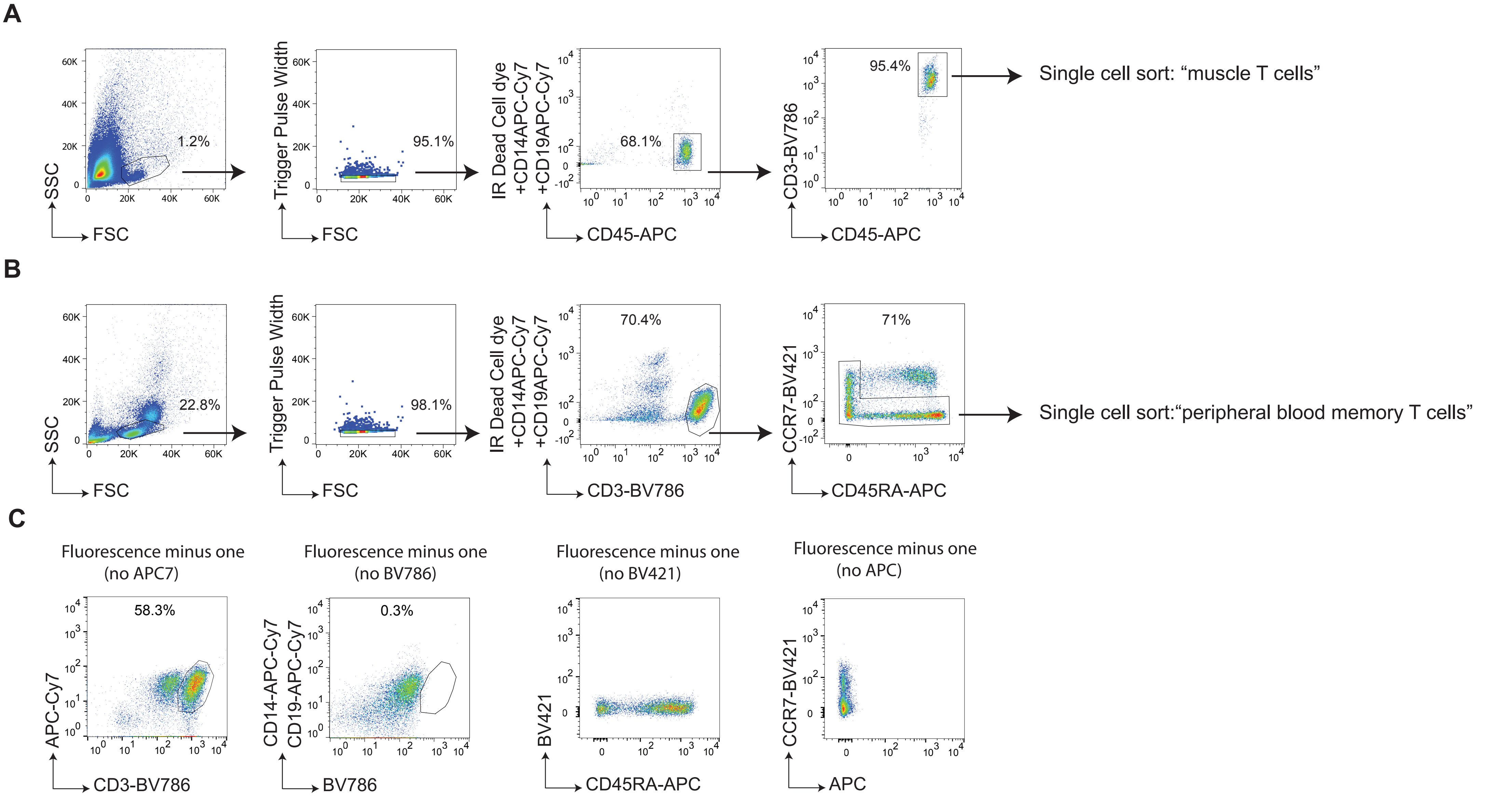

Appendix Figure S2. A) Flow cytometry gating strategy for single cell sorting of T cells from muscle biopsy included lymphocyte gating (based on forward and side scatter) followed by doublets, CD14+, CD19+ and dead cells exclusion.CD45+ CD3+ T cells were then further selected. Representative patient 6-IBM depicted. **B**) Flow cytometry gating strategy for single cell sorting of memory T cells from peripheral blood included lymphocyte gating (based on forward and side scatter) followed by doublets, CD14+,CD19+ and dead cells exclusion. CD3+ and memory T cells were then further selected (by excluding CCR7+ CD45RA+ cells). Representative patient 6-IBM depicted. **C**) Fluoroscence minus one controls performed on peripheral blood from an healthy donor.

Appendix Figure S3

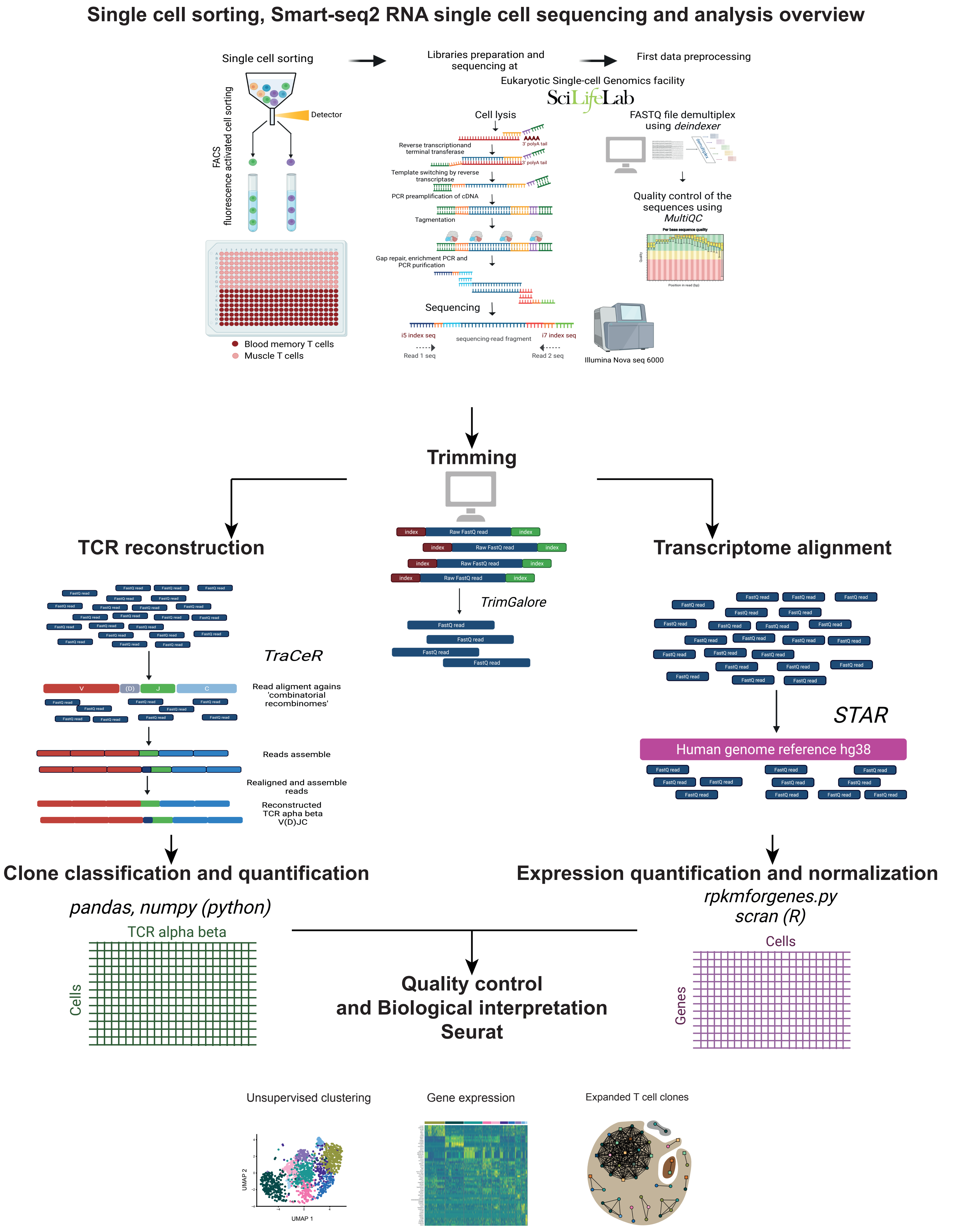

Appendix Figure 3. Workflow showing the methodology used for the RNA single-cell sequencing. The procedure starts with single-cell sorting, library preparation, and smart-seq2 single cell RNA sequencing, followed by data processing and analysis representation. The analysis contains two branches, one for the T cell receptor (TCR) reconstruction and the other for the transcriptome analyses. These branches converged in the biological interpretation using python and Seurat in R.

Appendix Table S1. Reagents

Flow cytometry single cell sorting antibodies				
**Antibody**	**Fluorochrome**	**Clone**	**Source**	**Reference**
CD3	BV786	SK7	BD Bioscience	563800
CD4	APC	RPAT4	BD Bioscience	561841
CD4	BV421	RPAT4	BD Bioscience	562424
CD8	BB515	RPAT8	BD Bioscience	564526
CCR7	BV421	G043H7	Biolegend	353208
CD45RA	PECY7	L48	BD Bioscience	337186
CD45	APC	HI30	BD Bioscience	561864
CD14	APC-H7	MΦP9	BD Bioscience	560180
CD19	APC-H7	SJ25C1	BD Bioscience	560177
gd	PE	REA 591	Miltenyi Biotec	130-114-038

Confocal antibodies				
**Antibody**	**Fluorochrome**	**Clone**	**Source**	**Reference**
mouse anti-human CD3	unconjugated	SK7	BD Bioscience	347340
rabbit anti-human HOBIT	unconjugated	polyclonal	ThermoFisher	PA5-54902
rabbit anti-human Dystrophin	Alexa Fluor 647	monoclonal	abcam	ab282171
mouse IgG1	unconjugated	monoclonal	DAKO	X0931
rabbit IgG	unconjugated	polyclonal	Invitrogen	02-6102
goat anti-mouse	Alexa Fluor 488	polyclonal	ThermoFisher	A-11029
goat anti-rabbit	Alexa Fluor 647	polyclonal	Cell Signaling	4414
donkey anti-rabbit	Cy3	polyclonal	Jackson Immunoresearch	711-165-152

Dyes

Name	Source	Reference
Hoeschst 333342	ThermoFischer	3570
Near-IR Dead Cell Stain Kit	Invitrogen	L10119

Appendix Table S2. Characteristics of additional patients used for immunofluorescence and immunohistochemistry stainings

Patient number	Diagnosis	Age interval at onset	Disease duration at time of biopsy (months)	Manual Muscle Testing (MMT8) (a)	Extramuscular global assessment (VAS) (b)	PhyGA (VAS) (c)	Health Assessment Questionnaire (HAQ)(d)	Health-related quality of life EQ-5D-5L (e)	Creatinine Kinase (CK, microcat/l)	Extra-muscular manifestations	Myositis-specific autoantibodies (MSA)	Antinuclear Antibodies (ANA)	Treatment at time of biopsy	Histology report	Flow cytometry immune infiltrate (f)	Biopsy location
16	IBM	70-75	120	66	32		NA	0.552	5.6	Dysphagia	negative	Negative	Methotrexate	-MHCI in several fibers -CD3 and CD8 in necrotic fibers	NA	Anterior tibialis (right)
17	IBM	65-70	24	45	20		NA	NA	6.6		SSA/SSB+	ANA+	Methotrexate	NA	NA	Vastus lateralis (left)
18	IBM	60-65	24	68	NA		NA	NA	5.4		NA	NA	Azathioprine	-MHCI staining -CD3 cell infiltration	NA	Vastus lateralis (right)
