# Supplementary material for: Single‐cell profiling of muscle‐infiltrating T cells in idiopathic inflammatory myopathies
Source: EMBO Mol Med. 2023 Jul 31;15(10):e17240. doi: 10.15252/emmm.202217240 (PMC10565639; doi:10.15252/emmm.202217240)
Supplement: Supplementary file 2 — Expanded View Figures PDF [file EMMM-15-e17240-s001.pdf]

Expanded View Figures

Figure EV1. Smart-seq2 single-cell sequencing data from muscle T cells in patients with idiopathic inflammatory myopathies.

A Left panel: Violin plots showing the total number of genes captured, the number of transcripts and percentage of mitochondrial genes per patient. Right panel: UMAPs displaying before data integration and after data integration with Harmony package per sequencing batch (n = 6 IIM, n = 1 undefined myopathy).
B Violin plots showing the expression levels of selected differentially expressed genes per cluster in muscle T cells. Each color represents one T-cell cluster in the muscle tissue as indicated (n = 6 IIM, n = 1 undefined myopathy).
C Violin plot showing the expression level of *ITGA1* per patient in the four T_{RM} clusters. Each color represents one T_{RM} cluster as indicated (n = 6 IIM, n = 1 undefined myopathy).
D Violin plot showing the expression level of *KLRG1* in muscle T cells per patient.

Data information: CM: central memory; EM: effector memory, T_{RM}: tissue resident memory. IMNM: Immune-Mediated Necrotizing Myopathy; DM: DermatoMyositis; ASyS: AntiSYnthetase Syndrome; IBM: Inclusion Body Myositis; IIM: Idiopathic Inflammatory Myopathies (n = 6 IIM, n = 1 undefined myopathy).

 © 2023 The Authors

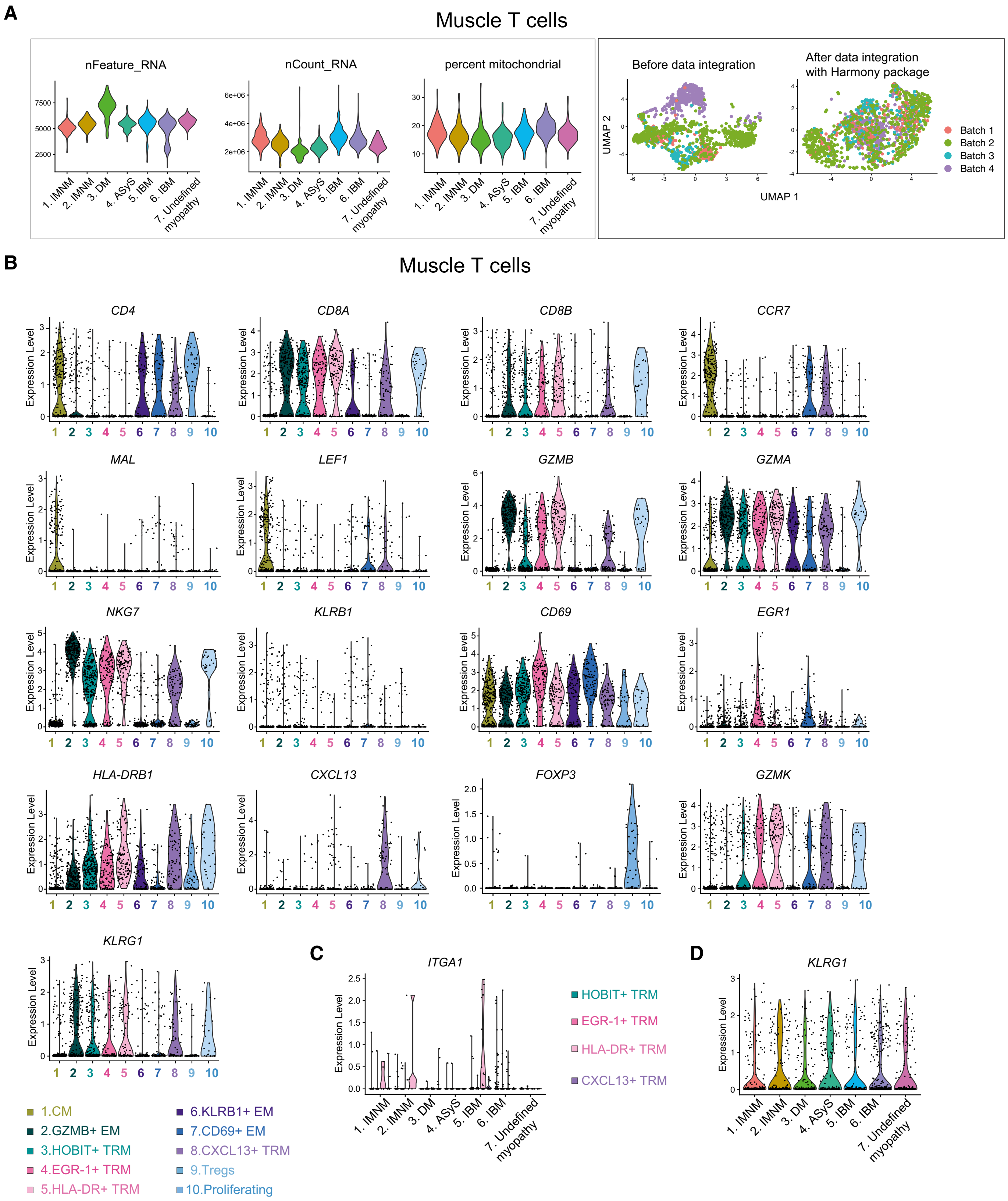
A
Muscle T cells
nFeature_RNA
nCount_RNA
percent mitochondrial
1. IMNM
2. IMNM
3. DM
4. ASyS
5. IBM
6. IBM
7. Undefined myopathy
Before data integration
After data integration with Harmony package
UMAP 2
UMAP 1
Batch 1
Batch 2
Batch 3
Batch 4
B
Muscle T cells
CD4
CD8A
CD8B
CCR7
MAL
LEF1
GZMB
GZMA
NKG7
KLRB1
CD69
EGR1
HLA-DRB1
CXCL13
FOXP3
GZMK
KLRG1
Expression Level
1.CM
2.GZMB+ EM
3.HOBIT+ TRM
4.EGR-1+ TRM
5.HLA-DR+ TRM
6.KLRB1+ EM
7.CD69+ EM
8.CXCL13+ TRM
9.Tregs
10.Proliferating
C
ITGA1
HOBIT+ TRM
EGR-1+ TRM
HLA-DR+ TRM
CXCL13+ TRM
D
KLRG1

Figure EV1.

Figure EV2. Smart-seq2 single-cell sequencing data from peripheral blood memory T cells in patients with idiopathic inflammatory myopathies.

A Left panel: Violin plots showing the total number of genes captured, the number of transcripts and percentage of mitochondrial genes per patient. Right panel: UMAPs displaying before data integration and after data integration with Harmony package per sequencing batch (n = 6 IIM, n = 1 undefined myopathy).
B Violin plots showing the expression level of selected genes in peripheral blood memory T cells from patients with IIM (n = 6 IIM, n = 1 undefined myopathy).
C UMAP feature plots displaying the normalized expression of *KLRC1*, *KLRC2*, *FCGR3A* and *IKZF2* genes.
D UMAP feature plot showing cells using *TRAV1-2* and *TRAV10* genes.
E Flow cytometry dot plots showing the frequency of $CD3^+CD8^+$ T cells in peripheral blood (PB) of patients with DM (left) and IMNM (right) patients.
F Frequency of $CD3^+CD8^+$ T cells in PB of patients with IIM.

Data information: CM: central memory; EM: effector memory. IMNM: Immune-Mediated Necrotizing Myopathy; DM: DermatoMyositis; ASyS: AntiSYnthetase Syndrome; IBM: Inclusion Body Myositis; IIM: Idiopathic Inflammatory Myopathies.

 © 2023 The Authors

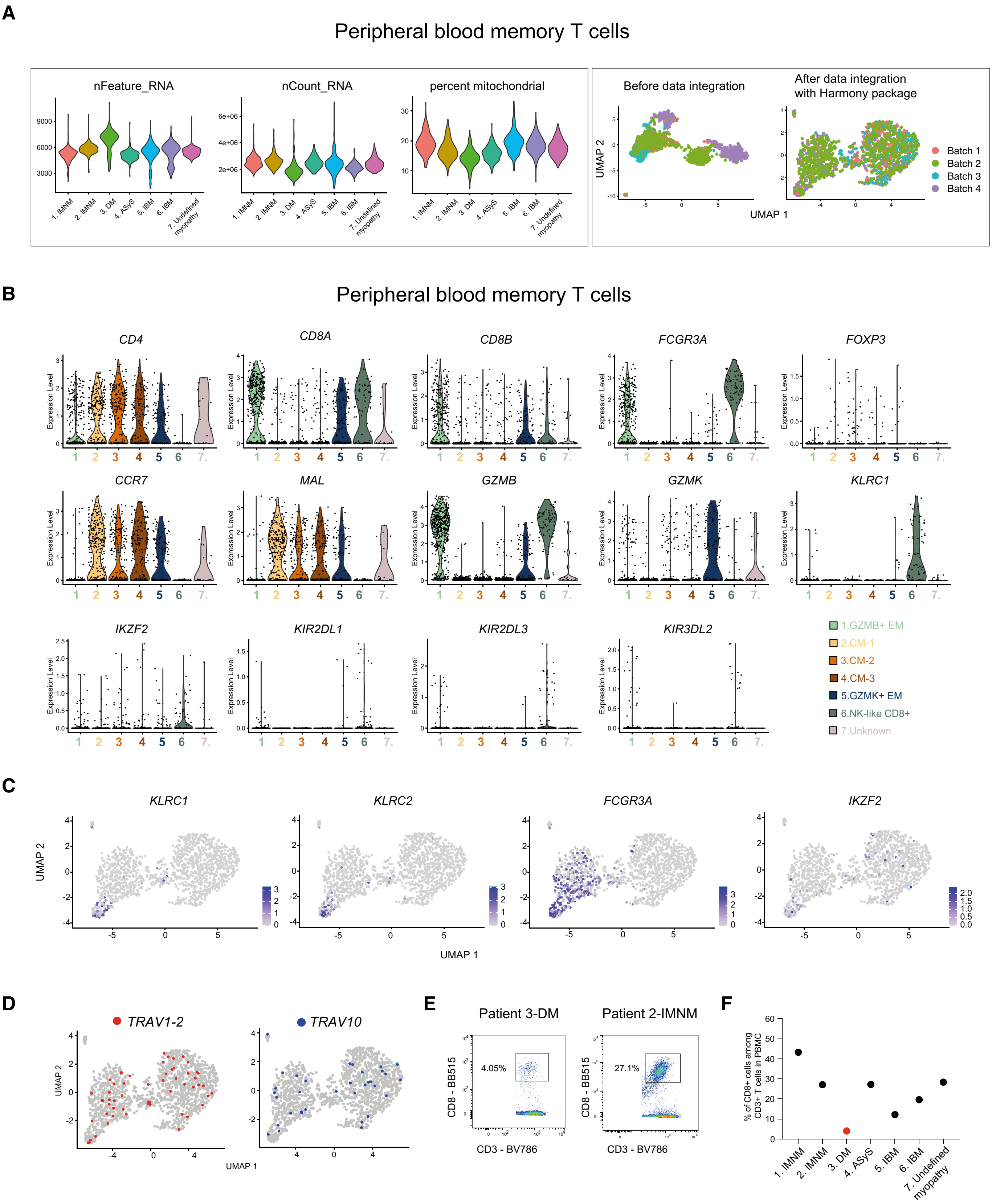
A
Peripheral blood memory T cells
nFeature_RNA
nCount_RNA
percent mitochondrial
1. IMNM
2. IMNM
3. DM
4. ASyS
5. IBM
6. IBM
7. Undefined myopathy
Before data integration
After data integration with Harmony package
UMAP 2
UMAP 1
Batch 1
Batch 2
Batch 3
Batch 4
B
Peripheral blood memory T cells
CD4
CD8A
CD8B
FCGR3A
FOXP3
CCR7
MAL
GZMB
GZMK
KLRC1
IKZF2
KIR2DL1
KIR2DL3
KIR3DL2
Expression Level
1.GZMB+ EM
2.CM-1
3.CM-2
4.CM-3
5.GZMK+ EM
6.NK-like CD8+
7.Unknown
C
KLRC1
KLRC2
FCGR3A
IKZF2
UMAP 2
UMAP 1
D
TRAV1-2
TRAV10
UMAP 2
UMAP 1
E
Patient 3-DM
Patient 2-IMNM
4.05%
27.1%
CD8 - BB515
CD3 - BV786
F
% of CD8+ cells among CD3+ T cells in PBMC
1. IMNM
2. IMNM
3. DM
4. ASyS
5. IBM
6. IBM
7. Undefined myopathy

Figure EV2.

Figure EV3. Single-cell sequencing data from healthy donors and patients with idiopathic inflammatory myopathies.

A Workflow for Smart-seq3 sequencing.
B UMAP displaying eight memory T-cell clusters identified in peripheral blood (PB) of healthy donors (n = 439 cells, n = 3 donors).
C UMAP feature plots displaying the normalized expression of *CD4*, *CD8A*, *CD8B*, *IKZF2*, *KLRC1* and *KLRC2*.
D Heatmap showing the normalized and scaled expression of the top 10 differentially expressed genes per cluster (Wilcoxon Rank Sum, P value < 0.009; $\log_2$ fold change > 0.25).
E Violin plots showing the expression level of *TRAV1-2* and *TRAV10* genes per cluster in PB memory T cells from healthy donors (n = 3).
F Heatmap showing the normalized and scaled expression of the top 25 upregulated and top 25 downregulated genes sorted by average log2FC values (Wilcoxon Rank Sum, P value $< 10^{-15}$, $-2.6 < \log_2$ fold change < 4.2).
G Heatmap showing the normalized and scaled expression of the top 25 upregulated and top 25 downregulated genes in muscle T cells versus PB memory T cells from patients with IIM after filtering out genes identified in Dataset EV5 (Wilcoxon Rank Sum, P value $< 8 \times 10^{-5}$, $-0.25 < \log_2$ fold change < 2.3).
H Dotplots showing selected genes in PB of patients with IIM (n = 7).
I Dotplots showing selected genes in PB of healthy donors (n = 3).

Data information: CM: central memory; EM: effector memory. IMNM: Immune-Mediated Necrotizing Myopathy; DM: DermatoMyositis; ASyS: AntiSYnthetase Syndrome; IBM: Inclusion Body Myositis; IIM: Idiopathic Inflammatory Myopathies; HD: healthy donors.

© 2023 The Authors

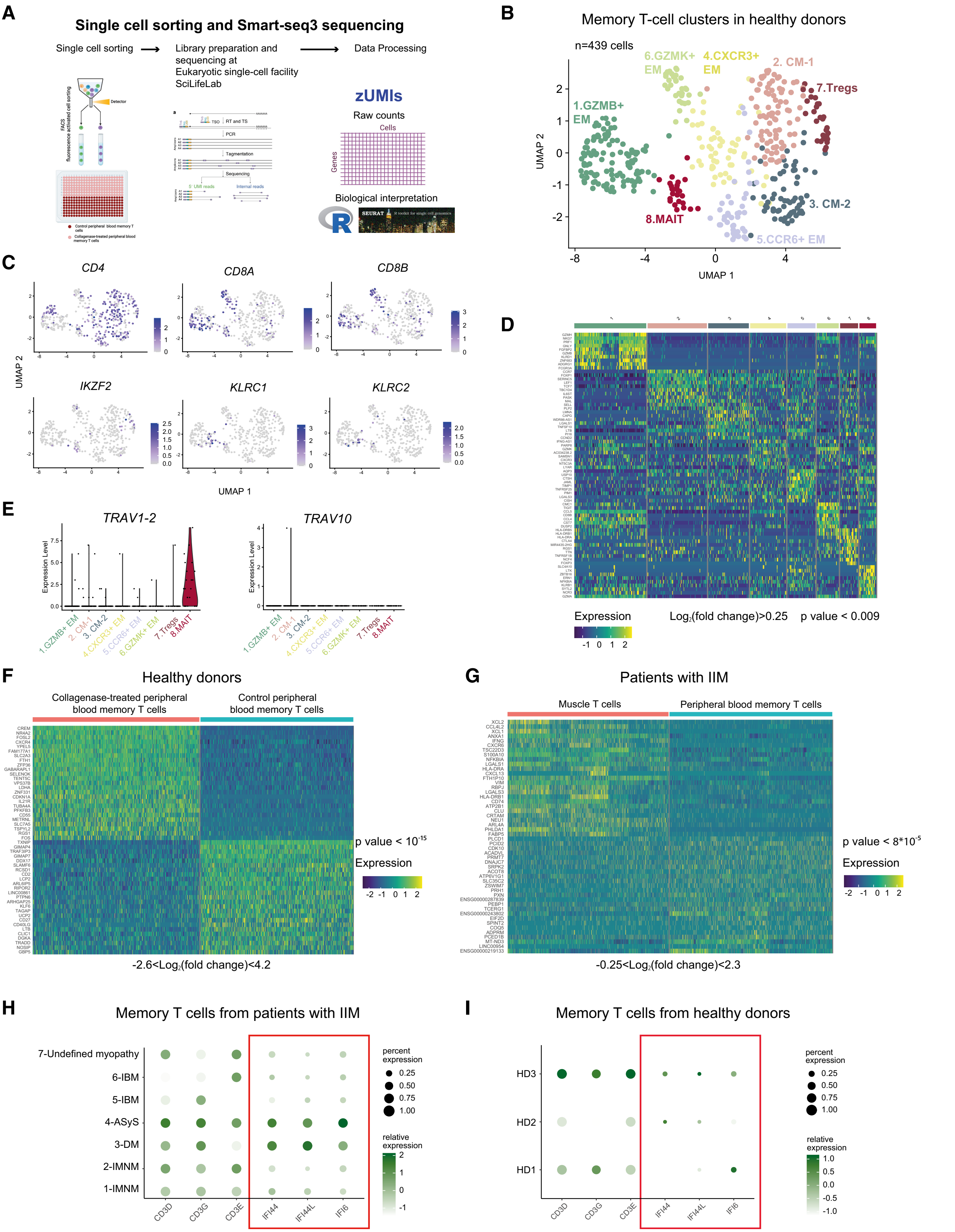
A
Single cell sorting and Smart-seq3 sequencing
Single cell sorting
Library preparation and sequencing at Eukaryotic single-cell facility SciLifeLab
Data Processing
zUMIs
Raw counts
Cells
Genes
Biological interpretation
Control peripheral blood memory T cells
Collagenase-treated peripheral blood memory T cells
B
Memory T-cell clusters in healthy donors
n=439 cells
1.GZMB+ EM
2. CM-1
3. CM-2
4.CXCR3+ EM
5.CCR6+ EM
6.GZMK+ EM
7.Tregs
8.MAIT
UMAP 1
UMAP 2
C
CD4
CD8A
CD8B
IKZF2
KLRC1
KLRC2
UMAP 1
UMAP 2
D
Expression
Log2(fold change)>0.25
p value < 0.009
E
TRAV1-2
TRAV10
Expression Level
F
Healthy donors
Collagenase-treated peripheral blood memory T cells
Control peripheral blood memory T cells
p value < 10-15
Expression
-2.6<Log2(fold change)<4.2
G
Patients with IIM
Muscle T cells
Peripheral blood memory T cells
p value < 8*10-5
Expression
-0.25<Log2(fold change)<2.3
H
Memory T cells from patients with IIM
7-Undefined myopathy
6-IBM
5-IBM
4-ASyS
3-DM
2-IMNM
1-IMNM
CD3D
CD3G
CD3E
IFI44
IFI44L
IFI6
percent expression
relative expression
I
Memory T cells from healthy donors
HD3
HD2
HD1
CD3D
CD3G
CD3E
IFI44
IFI44L
IFI6
percent expression
relative expression

Figure EV3.

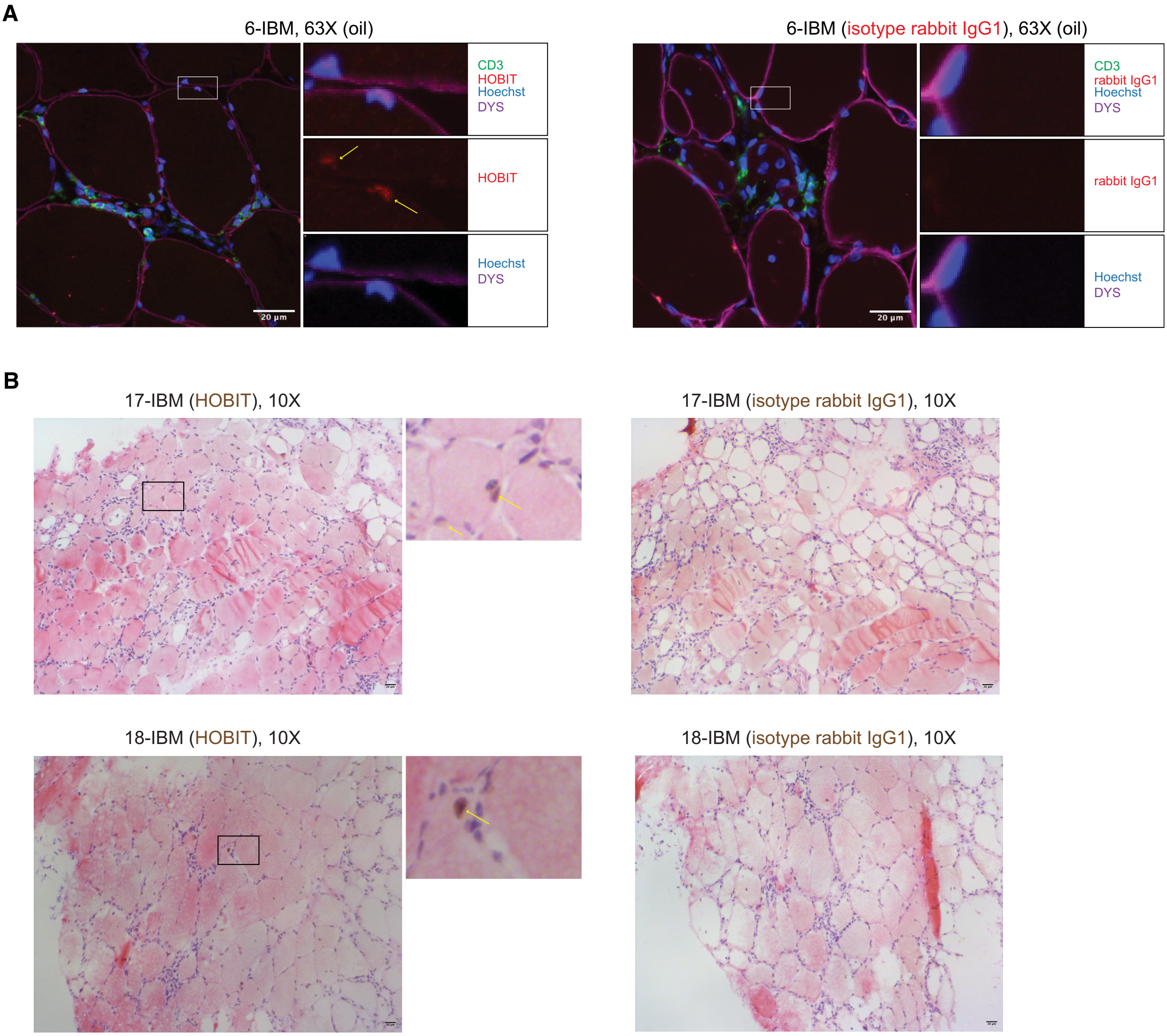

Figure EV4. Immunofluorescence and immunohistochemistry stainings showing HOBIT expression in muscle fibers of biopsies from patients with inclusion body myositis.

A Representative immunofluorescence stainings of HOBIT (red), CD3 (green), dystrophin (purple) and Hoechst 33342 (blue) performed on muscle tissue from patient 6 (IBM) (left panel) and isotype rabbit IgG1 staining (red) (right panel). Images were acquired using a LSM 880 confocal without Airyscan microscope (63× oil objective). Scale bar = 20 μm. The yellow arrows show HOBIT expression in muscle nuclei.

B Representative immunohistochemistry stainings of HOBIT (brown, left panels), and isotype rabbit IgG (right panels) with hematoxylin (purple) and eosin (red) stainings performed on muscle tissue of two patients with IBM. Images were acquired using a Leica Reichert Polyvar 2 light microscope (10× objective). Scale bar = 20 μm. The yellow arrows show HOBIT expression in muscle nuclei. IBM: Inclusion Body Myositis.

© 2023 The Authors

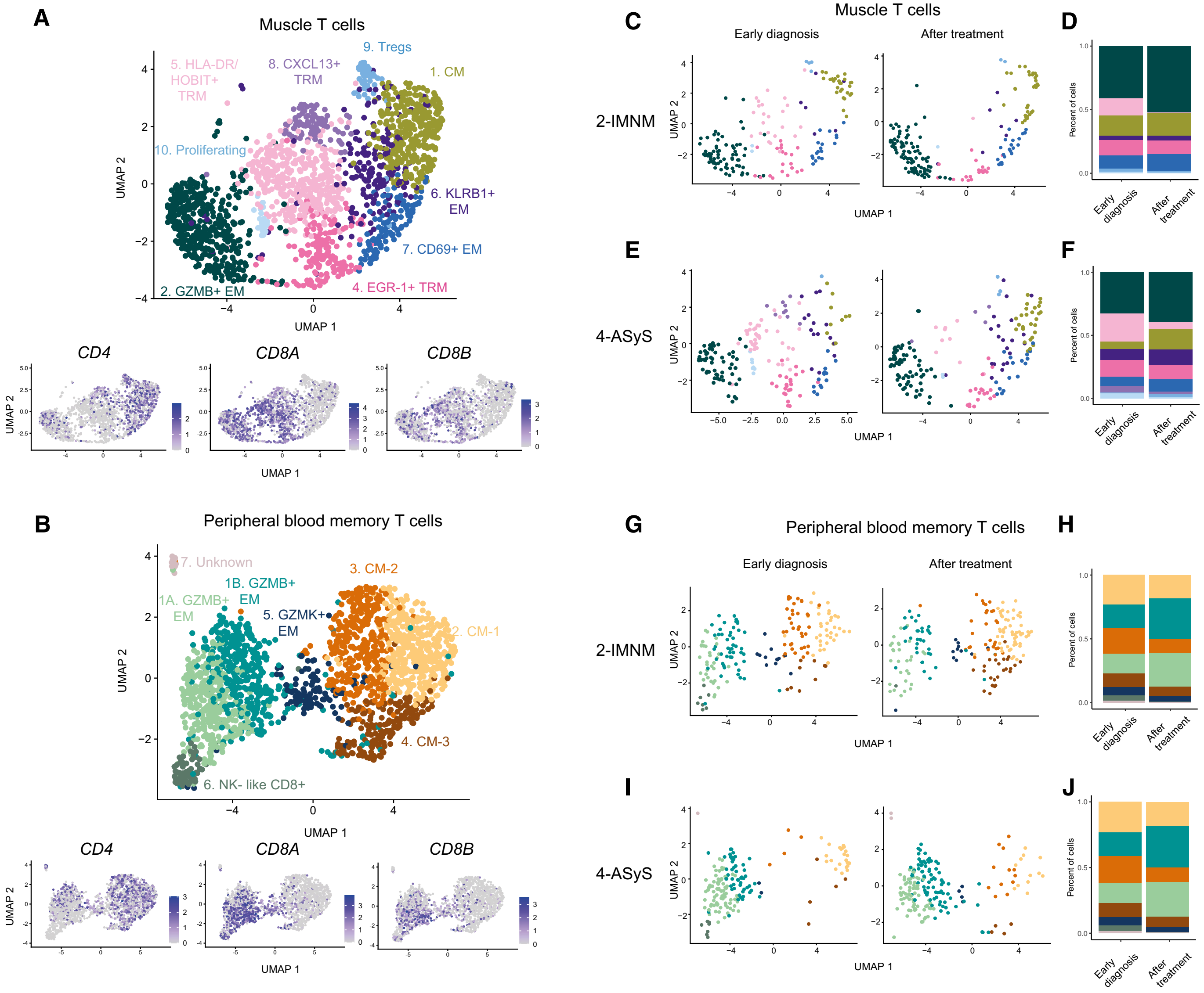

Figure EV5. Smart-seq2 single-cell data from muscle T cell and peripheral blood memory T cells from patients at early diagnosis and after treatment.

A Upper Panel: UMAP displaying nine T-cell clusters in the muscle of patients with idiopathic inflammatory myopathies (IIM) at early diagnosis ($n = 7$) and after treatment ($n = 2$) ($n = 1747$ cells). Lower panel: UMAP feature plots displaying the normalized expression of *CD4*, *CD8A*, and *CD8B*.

B Upper panel: UMAP displaying eight T-cell clusters in peripheral blood (PB) memory T cells of patients with IIM at early diagnosis ($n = 7$) and after treatment ($n = 2$) ($n = 1788$ cells). Lower panel: UMAP feature plots displaying the normalized expression of *CD4*, *CD8A* and *CD8B*.

C UMAP displaying nine T-cell clusters split by treatment status in muscle of patient 2 (IMNM).

D Stacked bar plots of T-cell cluster composition in early diagnosis and after treatment in muscle of patient 2 (IMNM).

E UMAP displaying nine T-cell clusters split by treatment status in muscle of patient 4 (ASyS).

F Stacked bar plots of T-cell cluster composition in early diagnosis and after treatment in muscle of patient 4 (ASyS).

G UMAP displaying eight T-cell clusters split by treatment status in PB of patient 2 (IMNM).

H Stacked bar plots of T-cell cluster composition in early diagnosis and after treatment in PB of patient 2 (IMNM).

I UMAP displaying eight T-cell clusters split by treatment status in PB of patient 4 (ASyS).

J Stacked bar plots of T-cell cluster composition in early diagnosis and after treatment in PB of patient 4 (ASyS).

Data information: IMNM: Immune-Mediated Necrotizing Myopathy; ASyS: AntiSYnthetase Syndrome; IIM: Idiopathic Inflammatory Myopathies.

© 2023 The Authors
