# Supplementary material for: Single‐cell profiling of muscle‐infiltrating T cells in idiopathic inflammatory myopathies
Source: EMBO Mol Med. 2023 Jul 31;15(10):e17240. doi: 10.15252/emmm.202217240 (PMC10565639; doi:10.15252/emmm.202217240)
Supplement: Supplementary file 16 — PDF+ [file EMMM-15-e17240-s008.pdf]

Resource

SOURCE DATA | TRANSPARENT PROCESS | OPEN ACCESS

EMBO Molecular Medicine

Single-cell profiling of muscle-infiltrating T cells in idiopathic inflammatory myopathies

Alexandra Argyriou[1,2,†], Begum Horuluoglu[1,2,†], Angeles Shunashy Galindo-Feria[1,2,†], Juan Sebastian Diaz-Boada[1,2], Merel Sijbranda[1,2], Antonella Notarnicola[1,2,3], Lara Dani[1,3], Annika van Vollenhoven[1,2], Daniel Ramsköld[4], Inger Nennesmo[5], Maryam Dastmalchi[1,2,3], Ingrid E Lundberg[1,2,3], Lina-Marcela Diaz-Gallo[1,2,‡] & Karine Chemin[1,2,*,‡]

Abstract

Idiopathic inflammatory myopathies (IIM) are rare autoimmune systemic diseases characterized by muscle weakness and the presence of muscle-infiltrating T cells. IIM represent a clinical challenge due to heterogeneity of symptoms and variability of response to immunosuppressive treatment. Here, we performed in-depth single-cell sequencing on muscle-infiltrating T cells and peripheral blood memory T cells in six patients with recently diagnosed IIM. We identified tissue resident memory T-cell (T_{RM}) signatures including the expression of *HOBIT*, *XCL1* and *CXCR6* in the muscle biopsies of all patients with IIM. Clonally expanded T-cell clones were mainly found among cytotoxic and T_{RM} implying their role in the disease pathogenesis. Finally, identical expanded T-cell clones persisting at follow-up in the muscle tissue of two patients suggest their involvement in disease chronicity. Our study reveals a muscle tissue resident memory T-cell signature in patients with IIM and a transcriptomic map to identify novel therapeutic targets in IIM.

Keywords HOBIT; idiopathic inflammatory myopathies; muscle; T-cell receptor; tissue resident memory T cells
Subject Categories Immunology; Musculoskeletal System
DOI 10.15252/emmm.202217240 | Received 29 November 2022 | Revised 4 July 2023 | Accepted 10 July 2023 | Published online 31 July 2023
EMBO Mol Med (2023) 15: e17240
See also: **M Yazdani *et al*** (October 2023)

Introduction

Idiopathic inflammatory myopathies (IIM) or myositis are rare, chronic, systemic autoimmune diseases characterized by skeletal muscle inflammation and varying degrees of immune infiltrates in the skeletal muscle (Furst *et al*, 2012; Svensson *et al*, 2017; Dobloug *et al*, 2018; Lundberg *et al*, 2018). IIM are divided into the major subgroups dermatomyositis (DM), juvenile dermatomyositis (JDM), clinical amyopathic dermatomyositis (CADM), inclusion body myositis (IBM), immune-mediated necrotizing myopathy (IMNM), anti-synthetase syndrome (ASyS) and polymyositis (PM) (Medsger *et al*, 1970; Bohan & Peter, 1975; Love *et al*, 1991; Troyanov *et al*, 2005). Most patients with IIM experience muscle weakness suggesting a common pathogenic mechanism affecting skeletal muscle tissue. Immunosuppressive agents in combination with physical exercise are used to treat patients with IIM, however, often with disappointing results and complete recovery of muscle performance is rarely achieved. In particular, patients with IBM rarely respond to immunosuppressive treatment and develop progressive muscle atrophy and disability (Barsotti & Lundberg, 2018).

Although the causes of myositis are still unknown, genome-wide association studies (GWAS) have identified *HLA-DR* (Miller *et al*, 2015; Rothwell *et al*, 2016, 2019) as the strongest locus associated with the disease implicating that antigen presentation and subsequent T-cell activation are implicated in pathogenesis. This hypothesis is supported by the identification of T-cell infiltrates in muscle biopsies of patients with IIM which have been described for decades (Arahata & Engel, 1984, 1986; Malmstrom *et al*, 2012) and is important for the diagnosis and classification of IIM (Dorph *et al*, 2001). In the subset DM, mainly $CD4^+$ T cells were identified in the muscle tissue whereas in patients with PM or IBM, $CD8^+$ T cells are suggested to be the main pathogenic T-cell subset (Arahata & Engel, 1984; Dalakas & Sivakumar, 1996). However, the mechanisms involved in T-cell persistence in the skeletal muscle of patients with IIM during disease development and chronicity are currently unknown.

Here, we performed an unbiased single-cell profiling of muscle-infiltrating T cells and peripheral blood memory T cells in six

1 Division of Rheumatology, Department of Medicine, Solna, Karolinska Institutet, Stockholm, Sweden
2 Center for Molecular Medicine, Karolinska Institutet, Stockholm, Sweden
3 Department of Gastro, Dermatology and Rheumatology, Karolinska University Hospital, Stockholm, Sweden
4 Department of Cell and Molecular Biology, Karolinska Institutet, Stockholm, Sweden
5 Department of Oncology-Pathology, Karolinska University Hospital, Stockholm, Sweden
*Corresponding author. Tel: +46 08 524 800 00; E-mail: karine.chemin@ki.se
†These authors contributed equally to this work as first authors
‡These authors contributed equally to this work as senior authors

© 2023 The Authors. Published under the terms of the CC BY 4.0 license

recently diagnosed patients with IIM and in a patient with an undefined myopathy as a comparator. We identified T cells expressing a tissue resident memory (T_{RM}) signature including the transcription factor *HOBIT* in the muscle of all patients with IIM. Clonally expanded muscle T cells displayed cytotoxic or T_{RM} features. The same expanded T-cell clones were identified at early diagnosis and after treatment in two patients. Altogether, our study provides an immune profiling map of T cells in patients with IIM which contributes to the understanding of the pathogenic mechanisms and may help to identify potential new therapies.

Results

CD8$^+$ and CD4$^+$ T-cell populations in the muscle of patients with myositis

First, we evaluated the presence of T-cell infiltrates in fresh muscle biopsies by flow cytometry (Fig 1A) and immunohistochemistry from patients with suspicion of myositis (Dataset EV1). T cells could be sorted by flow cytometry in seven out of 15 patients corresponding to the identification of T-cell infiltrates in the muscle by histopathology (Fig 1B, Appendix Fig S1). We then performed Smart-seq2 single-cell RNA sequencing on T cells isolated from fresh muscle biopsies (hereafter named “muscle T cells”) and matched peripheral blood (PB) of six patients with a confirmed IIM diagnosis and one patient with an undefined myopathy (Fig 1A and Dataset EV1). Muscle T cells were sorted based on the co-expression of CD45 and CD3 whereas PB memory T cells were sorted based on the exclusion of naïve CD45RA$^+$CCR7$^+$CD3$^+$ T cells (gating strategy in Appendix Fig S2). After quality control, filtering, and normalization of the gene expression matrix (Appendix Fig S3, Fig EV1A), 1,402 muscle T cells were recovered from the analysis. We then performed unsupervised clustering of muscle T cells which revealed 10 clusters annotated based on known genes and visualized by UMAP (Figs 1C–F and EV1B, Dataset EV2). Cluster 1 (CM) contained central memory T cells expressing *MAL*, *CCR7* (Fig EV1B) and *LEF1* (Szabo *et al*, 2019) and was enriched in CD4$^+$ T cells. Several clusters with effector memory features were detected. Cluster 2 (GZMB$^+$ EM) identified effector memory T cells, expressed high levels of *GZMB*, *PRF1* and *NKG7* and consisted of both CD4$^+$ and CD8$^+$ T cells. Cluster 6 (KLRB1$^+$ EM) and cluster 7 (CD69$^+$ EM) represented two clusters of effector memory T cells expressing low levels of *CCR7*, *KLRB1* (encoding CD161) and *CD69*, mainly composed of CD4$^+$ T cells. Four clusters of T cells enriched in tissue resident memory (T_{RM}) signatures (*XCL1*, *XCL2*, *CXCR6* (Wein *et al*, 2019) and *CRTAM* (Cortez *et al*, 2014)) were identified: cluster 3 (HOBIT$^+$ T_{RM}) expressing *ZNF683* (Mackay *et al*, 2016; Parga-Vidal *et al*, 2021; encoding HOBIT) and enriched in CD8$^+$ T cells; cluster 4 (EGR1$^+$ T_{RM}) expressing *EGR1*, *IFNG* and enriched in CD8$^+$ T cells; cluster 5 (HLA-DR$^+$ T_{RM}) expressing *CD74*, *HLA-DRB1* and *GZMB* and enriched in CD8$^+$ T cells; cluster 8 (CXCL13$^+$ T_{RM}) expressing *CXCL13*, *HLA-DRB1*, *TNFRSF9* and containing both CD8$^+$ and CD4$^+$ T cells. Cluster 9 (Tregs) was composed of regulatory CD4$^+$ T cells expressing *FOXP3* (Zemmour *et al*, 2018), *TNFRSF9* and *TIGIT*. Cluster 10 (Proliferating) consisted of proliferating T cells expressing *MKI67* (Uxa *et al*, 2021) and was enriched in CD8$^+$ T cells. All T-cell clusters were detected in all patients at different frequencies (Fig 1F and G). While the CXCL13$^+$ T_{RM} cluster was more prominent in patient with DM, the HOBIT$^+$ T_{RM} cluster was larger in patient 6 with IBM. Patient 7 with the undefined myopathy presented with similar clusters as the patients with IIM although fewer T_{RM} cells were detected (Fig 1F and G). We further evaluated key T-cell receptors and transcription factors associated with T-cell effector functions within the clusters (Figs 1H and EV1B). GZMB$^+$ EM T cells (cluster 2) expressed genes associated with cytotoxic functions such as the receptors (*FGPBP2*, *ADGRG1*, *CX3CR1*, *KLRD1*, *KLRF1*, *KLRG1*), effector molecules (*GZMB*, *GZMA*, *GZMH*, *PRF1*, *NKG7*) and the *TBX21* transcription factor (Fig 1H). *GZMK* was expressed in T_{RM} clusters (clusters 4, 5, 8) but also in proliferating cells (cluster 10; Figs 1H and EV1B). EGR1$^+$ T_{RM} cells expressed *EGR1*, *IFNG*, *TNF* and the chemokines *CCL3*, *CCL4*, *CCL5* (Fig 1H). *CXCL13* was mainly detected in the CXCL13$^+$ T_{RM} (cluster 8) and to a lesser extent in the HLA-DR$^+$ T_{RM} subset (cluster 5). Together, our data show that in IIM, muscle infiltrating T cells are predominantly composed of cytotoxic, effector memory, tissue-resident, proliferating and regulatory T cells.

Figure 1. T-cell clusters identified in the muscle of patients with idiopathic inflammatory myopathies. ▶

A Methodological workflow including isolation of memory T cells from peripheral blood (PB, in green) and muscle T cells (in blue) from muscle biopsies, followed by Smart-seq2 single-cell sequencing in six patients with idiopathic inflammatory myopathies (IIM) and one with an undefined myopathy.
B Representative CD3 immunohistochemistry staining of a muscle biopsy from a patient with inclusion body myositis (6-IBM).
C UMAP displaying 10 T-cell clusters in the muscle of patients with IIM (n = 1,402 cells).
D UMAP feature plots displaying the normalized expression of *CD4*, *CD8A*, and *CD8B* genes.
E Heatmap showing the normalized and scaled expression of the top seven differentially expressed genes per cluster (Wilcoxon Ranked Sum test, P value < 0.006; $0.5 < \log_2$ fold change < 4.5) as well as *CD4*, *CD8B* and *CD8A* gene expression.[§]
F UMAP displaying 10 muscle T-cell clusters split by patient.
G Stacked bar plots of the muscle T-cell cluster composition in each patient with IIM. Color corresponds to muscle T-cell clusters depicted in (C).
H Dot plot showing the expression level of selected T-cell effector function related genes per cluster (n = 6 IIM, n = 1 undefined myopathy). Circle size indicates percentage of cells expressing, color intensity indicates average expression.

Data information: (C, D, F) Each dot represents one cell. (C, F) n indicates the number of cells. CM: central memory; EM: effector memory; TRM: tissue resident memory. IMNM: Immune-Mediated Necrotizing Myopathy; DM: DermatoMyositis; ASyS: AntiSYnthetase Syndrome; IBM: Inclusion Body Myositis, IIM: Idiopathic Inflammatory Myopathies.
Source data are available online for this figure.

[§]Correction added on 31 August 2023, after first online publication: Panel E has been corrected.

 © 2023 The Authors

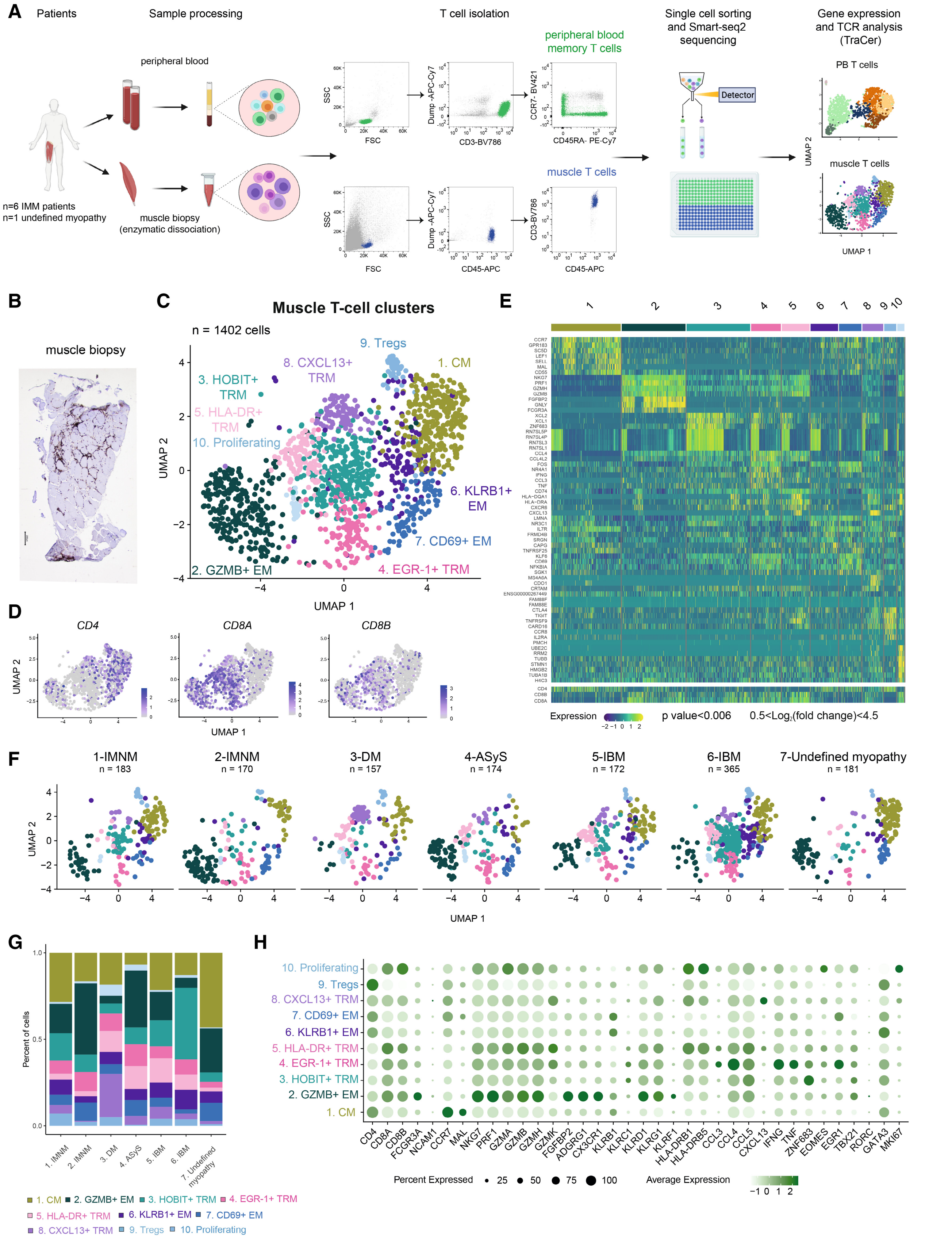
A
Patients
Sample processing
T cell isolation
Single cell sorting and Smart-seq2 sequencing
Gene expression and TCR analysis (TraCer)
peripheral blood
peripheral blood memory T cells
PB T cells
muscle T cells
muscle T cells
Detector
n=6 IMM patients
n=1 undefined myopathy
muscle biopsy (enzymatic dissociation)
B
muscle biopsy
C
Muscle T-cell clusters
n = 1402 cells
1. CM
2. GZMB+ EM
3. HOBIT+ TRM
4. EGR-1+ TRM
5. HLA-DR+ TRM
6. KLRB1+ EM
7. CD69+ EM
8. CXCL13+ TRM
9. Tregs
10. Proliferating
D
CD4
CD8A
CD8B
E
Expression
p value<0.006
0.5<Log2(fold change)<4.5
F
1-IMNM n = 183
2-IMNM n = 170
3-DM n = 157
4-ASyS n = 174
5-IBM n = 172
6-IBM n = 365
7-Undefined myopathy n = 181
G
Percent of cells
H
Percent Expressed
Average Expression

Figure 1.

$CD8^+$ and $CD4^+$ T-cell populations in peripheral blood of patients with myositis

After quality control, filtering, and normalization of the gene expression matrix (Appendix Fig S3, Fig EV2A), 1,417 PB memory T cells were recovered from the analysis. Unsupervised clustering of PB T cells revealed seven clusters annotated based on known genes and visualized by UMAP (Fig 2A–D, Dataset EV3). Three central memory clusters expressing *MAL* and *CCR7* were detected: cluster 2 (CM-1), cluster 3 (CM-2), and cluster 4 (CM-3) and were mainly composed of $CD4^+$ T cells (Figs 2B and EV2B). Two subsets of effector memory T cells were identified. Cluster 1 ($GZMB^+$ EM) expressing high levels of *GZMB*, *CX3CR1* (Bottcher *et al*, 2015), *ADGRG1* (Peng *et al*, 2011; Argyriou *et al*, 2022; encoding GPR56) and *PRF1* contained both $CD4^+$ and $CD8^+$ T cells. Cluster 5 ($GZMK^+$ EM) expressing high levels of *GZMK* was composed of both $CD4^+$ and $CD8^+$ T cells. We also detected $CD8^+$ T cells expressing NK-cell receptors (*KLRC1*, *KLRC2*, *FCGR3A*, and *KIRs* (encoding Killer Ig-Like receptors)), the transcription factor *IKZF2* (encoding HELIOS) in cluster 6 (NK-like CD8; Li *et al*, 2022; Fig EV2B and C). Of note, the invariant *TRAV1-2* (encoding Vα7.2) and *TRAV-10* (encoding Vα24) genes, expressed on MAIT (Treiner *et al*, 2003) and invariant NKT (Dellabona *et al*, 1994) cells respectively, were not enriched in a specific cluster (Fig EV2D). This NK-like $CD8^+$ T-cell cluster expressing *KLRC1*, *KLRC2*, *IKZF2* was not identified in Smart-seq3 single-cell data of memory T cells from PB of healthy donors (Fig EV3A–E, Dataset EV4). All populations were detected in all seven patients although memory PB T cells mainly consisted of $GZMB^+$ EM T cells in patient 4 with antisynthetase syndrome (ASyS; Fig 2D and E). Patient 3 with dermatomyositis (DM) had lower frequencies of $CD8^+$ T cells in PB compared to the other patients (clusters 1 and 6; Fig 2D and E) as confirmed by flow cytometry (Fig EV2E and F). Of note, in peripheral blood of patients with IIM, $FOXP3^+$ Tregs did not separate into a distinct cluster but were detected in several clusters (clusters 2, 3, 4, 5) including central and effector memory $CD4^+$ T cells (Figs 2F and EV2B). Our data show that, in patients with IIM, PB memory T cells are mainly composed of central and effector memory T cells and NK-like $CD8^+$ T cells.

Tissue resident memory T-cell signatures are detected in the muscle of patients with IIM

To further identify gene signatures in muscle T cells, we compared muscle and PB T-cell gene expression. We first evaluated gene expression in PB memory T cells from healthy donors treated with collagenase (Fig EV3F, Dataset EV5) and identified *CREM*, *FOSL2* and *CXCR4* among others as genes upregulated after collagenase treatment. We then compared differentially expressed genes between muscle T cells and PB memory T cells from patients with IIM, after removal of all the genes affected by enzymatic digestion (Fig EV3G). In the top 50 upregulated genes after the filtering, *XCL1*, *XCL2* (Mackay *et al*, 2016), *CXCR6* (Wein *et al*, 2019), *CRTAM* (Cortez *et al*, 2014) and *HOBIT* (Mackay *et al*, 2016; Parga-Vidal *et al*, 2021) associated with T_{RM} formation in different tissues, were upregulated in muscle T cells (Fig EV3G and Dataset EV6). Indeed, clusters 3, 4, 5, 8 showed a positive enrichment for a T_{RM} signature (Kumar *et al*, 2017) when compared to the other clusters (Fig 3A). *ITGA4* (encoding CD49D), *ITGAE* (encoding CD103), *CXCR6*, *CD69*, *CRTAM*, *ZNF683*, *XCL1* and *XCL2* were detected at different levels in $HOBIT^+$ T_{RM} (cluster 3), $EGR\text{-}1^+$ T_{RM} (cluster 4), $HLA\text{-}DR^+$ T_{RM} (cluster 5) and $CXCL13^+$ T_{RM} (cluster 8) cells (Fig 3B–D). The other classically associated tissue resident receptor *ITGA1* (encoding CD49a) had low expression in these cell populations (Fig EV1C). Downregulation of *S1PR1* is implicated in resident memory $CD8^+$ T-cell formation (Skon *et al*, 2013). Likewise, a lower expression of *S1PR* genes was detected in these four T_{RM} populations (clusters 3, 4, 5, 8) as compared to central or effector memory populations (Fig 3B and C). Moreover, $FOXP3^+$ Treg expressing *TIGIT* and *TNFRSF9* also displayed *CXCR6*, *TOX*, *PRDM1* (encoding BLIMP1) and low expression of *S1PR* genes suggesting a tissue resident memory signature (Fig 3B). *ZNF683* expression was higher in patients with IBM compared to the other patients (patients 5 and 6; Fig 3D). At the protein level, we confirmed the presence of HOBIT-positive T cells among muscle infiltrating T cells using confocal microscopy in patient 6 (IBM) and to a lesser extent in patient 3 (DM) and 4 (ASyS; Fig 3E–G). Interestingly, HOBIT was also detected in muscle fibers in biopsies (Fig EV4A and B). Thus, our results identify four T-cell subsets expressing tissue resident memory features, including the transcription factor HOBIT, in the muscle biopsies of patients with IIM.

Clonally expanded T cells are identified in the muscle

Local inflammation might induce recruitment of bystander T cells which can potentially participate in the pathogenesis of the disease (Whiteside *et al*, 2018). Investigation of T-cell clones might therefore be a more accurate mirror of ongoing autoimmune reactions. T-cell receptor sequences were assembled using TraCeR (Stubbington *et al*, 2016; Dataset EV7). As a clone, we defined two or more cells that shared the same CDR3 amino acid sequence of the T-cell receptor alpha (TCRα) and beta (TCRβ) chains. Expanded T-cell clones

Figure 2. T-cell clusters identified in peripheral blood of patients with idiopathic inflammatory myopathies. ▶

A UMAP displaying seven T-cell clusters identified in peripheral blood (PB) of patients with IIM (n = 1,417 cells).
B UMAP feature plots displaying the normalized expression of *CD4*, *CD8A*, and *CD8B* genes.
C Heatmap showing the normalized and scaled expression of the top 10 differentially expressed genes per cluster (Wilcoxon Rank Sum test, P value < 0.005; 0.57 < $\log_2$ fold change < 3.38) as well as *CD4*, *CD8B* and *CD8A* gene expression.
D UMAP displaying seven PB T-cell clusters split by patient.
E Stacked bar plots of the PB T-cell cluster composition in each patient with IIM. Color corresponds to the PB T-cell clusters depicted in (A).
F Dot plot showing the expression level of selected T-cell effector function related genes per cluster (n = 6 IIM, n = 1 undefined myopathy). Circle size indicates percentage of cells expressing, color intensity indicates average expression.

Data information: (A, B, D) Each dot represents one cell. (A, D) n indicates the number of cells. CM: central memory; EM: effector memory; IMNM: Immune-Mediated Necrotizing Myopathy; DM: DermatoMyositis; ASyS: AntiSYnthetase Syndrome; IBM: Inclusion Body Myositis, IIM: Idiopathic Inflammatory Myopathies.
Source data are available online for this figure.

© 2023 The Authors

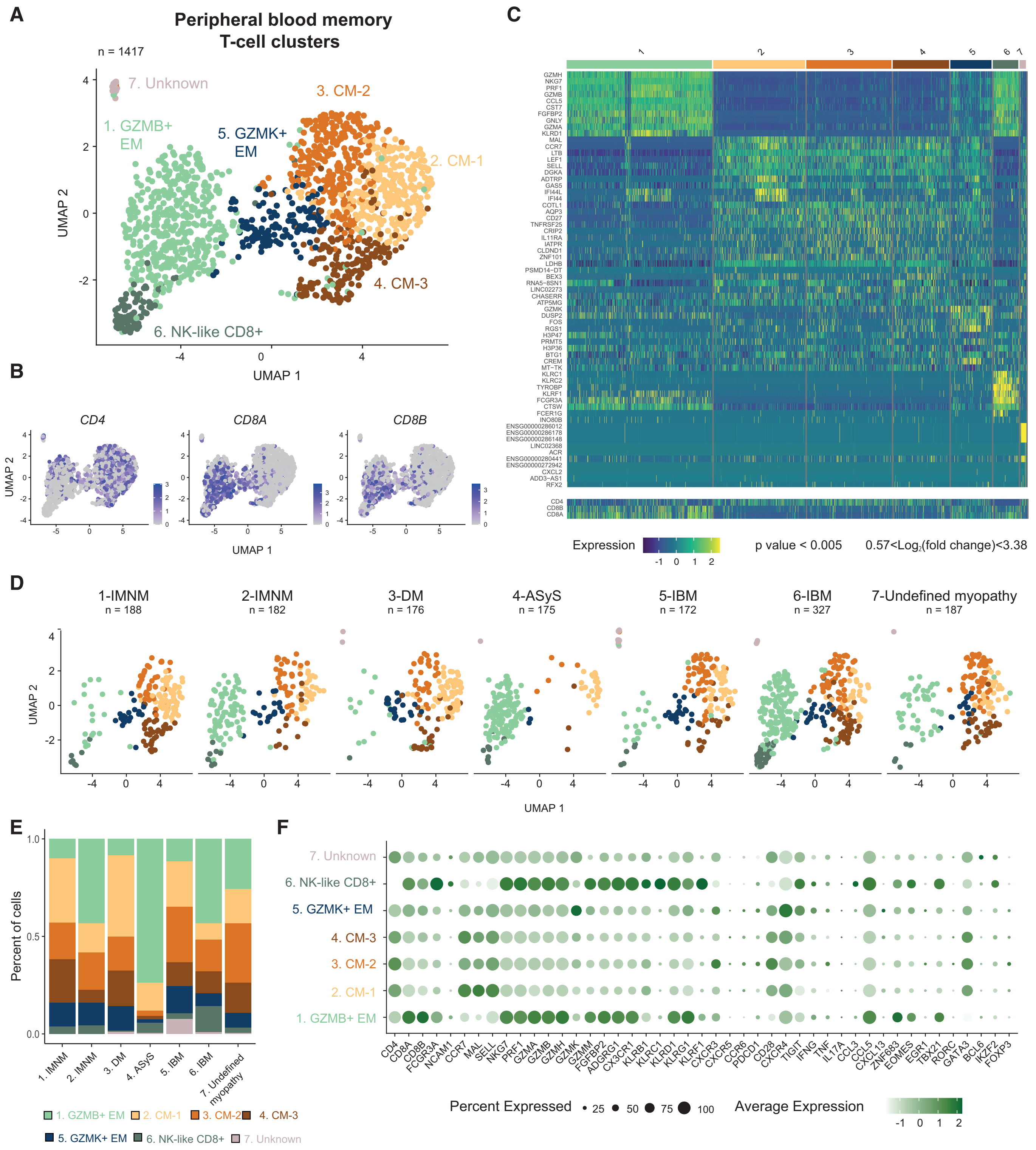

Figure 2.

were observed in the muscle tissue of all patients ranging between 20 to 50% of all CDR3 sequences (Fig 4A, left panel). In PB, expanded T-cell clones were mainly identified in patients 2 (IMNM), 4 (ASyS), and 6 (IBM; Fig 4A, right panel). In the two patients with IMNM, most expanded clones were identified among GZMB$^+$ EM CD8$^+$ T cells and were shared between PB and muscle (Fig 4B, Dataset EV8). Fewer clones were detected in the patient with DM and consisted of 2–3 cells. Patient 4 with ASyS presented expanded muscle GZMB$^+$ EM CD4$^+$ and CD8$^+$ T-cell clones with cytotoxic features (Fig 4B) sharing CDR3 sequences with PB GZMB$^+$ EM T cells. Most muscle expanded T-cell clones from the patients with IBM (patients 5 and 6) were among central memory, T_{RM} and KLRB1$^+$

© 2023 The Authors

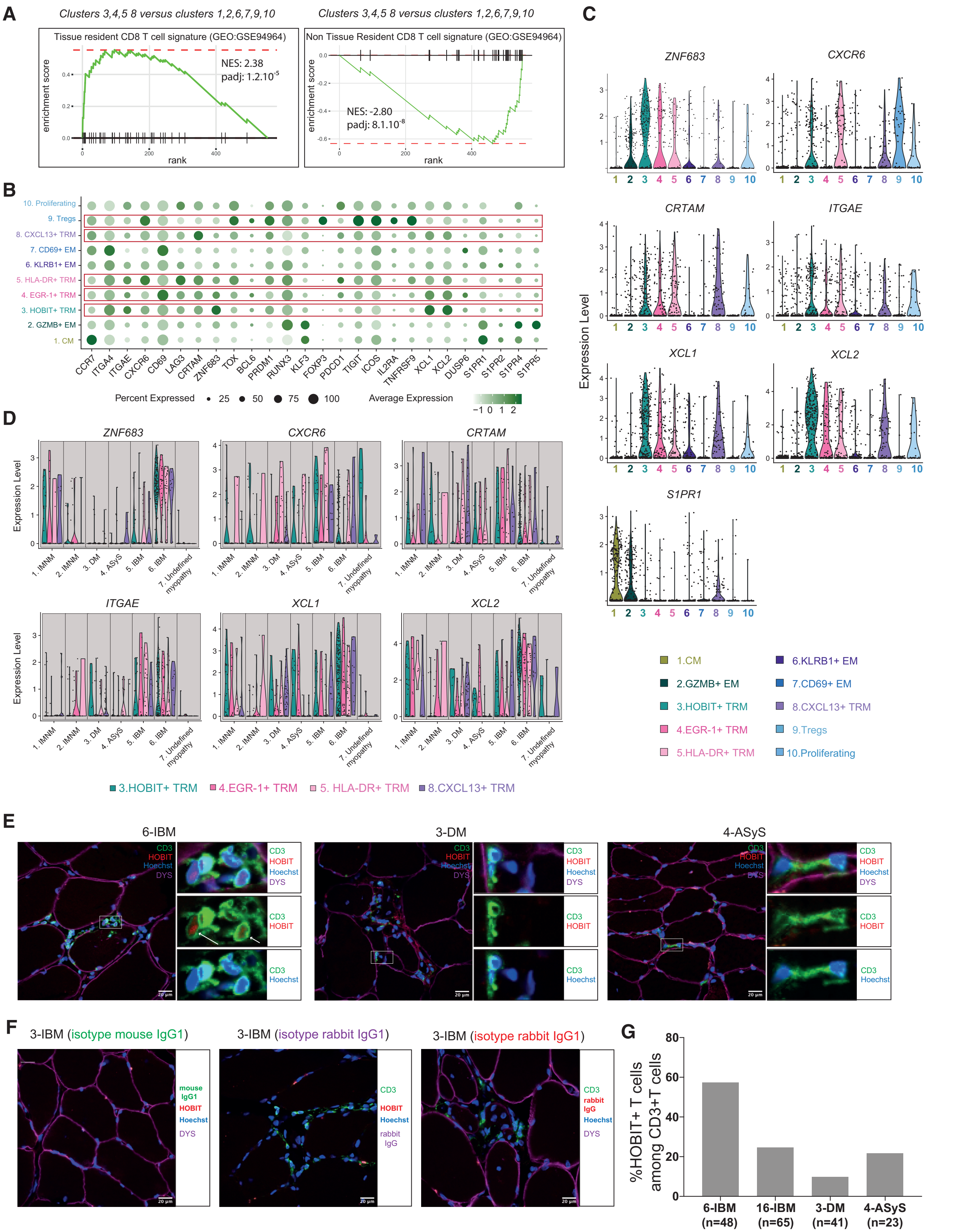

Figure 3.

© 2023 The Authors

Figure 3. Tissue resident memory and regulatory T-cell signatures in the muscle tissue of patients with idiopathic inflammatory myopathies.

A Gene Set Enrichment Analysis (GSEA) plot comparing muscle T-cell clusters 3, 4, 5, 8 versus clusters 1, 2, 6, 7, 9, 10 for the T_{RM} signature described in Kumar *et al*, 2017.

B Dot plot showing the normalized expression of selected genes coding for T_{RM} and regulatory (Tregs) signatures per cluster (n = 6 IIM, n = 1 undefined myopathy). Circle size indicates percentage of cells expressing, color intensity indicates average expression.

C Violin plots displaying the normalized expression level of selected genes per cluster in muscle T cells (n = 6 IIM, n = 1 undefined myopathy).

D Violin plots displaying the normalized expression level of selected genes per patient in the four T_{RM} clusters (n = 6 IIM, n = 1 undefined myopathy).

E Representative immunofluorescence stainings of HOBIT (red), CD3 (green), dystrophin (purple) and Hoechst 33342 (blue) performed on muscle tissue for patient 6 (IBM), patient 3 (DM) and 4 (ASyS). Images were acquired using a LSM 880 confocal without Airyscan microscope (63× oil objective). Scale bar = 20 μm. The white arrows show $HOBIT^+$ T cells.

F Isotype mouse and rabbit IgG1 immunofluorescence stainings.

G Quantification of $HOBIT^+$ T cells in muscle biopsies of patient 6 (IBM), patient 16 (IBM), patient 3 (DM) and 4 (ASyS). The number of counted CD3-positive T cells is indicated under each bar as n.

Data information: CM: central memory; EM: effector memory; TRM: tissue resident memory; Tregs: regulatory T cells. IMNM: Immune-Mediated Necrotizing Myopathy; DM: DermatoMyositis; ASyS: AntiSYnthetase Syndrome; IBM: Inclusion Body Myositis; IIM: Idiopathic Inflammatory Myopathies.
Source data are available online for this figure.

EM T cells. The patient with undefined myopathy mainly presented clones within $GZMB^+$ EM $CD4^+$ T cells which were shared with PB $GZMB^+$ EM T cells. However, there were very few expanded T_{RM} in this patient (Fig 4B). Sharing of T-cell clonality might reveal a differentiation link between different clusters. In muscle, we observed shared CDR3 sequences between central memory T cells (CM), $KLRB1^+$ EM T cells and the four T_{RM} clusters ($HOBIT^+$ T_{RM}, EGR-1^+ T_{RM}, HLA-DR^+ T_{RM}, $CXCL13^+$ T_{RM}) in patients 5 and 6. Of note, we did not detect any T-cell clones shared between patients. Altogether, these results show that most expanded clones were found among effector memory T cells with cytotoxic features and tissue resident memory T cells in the muscle of patients with IIM.

Expanded T-cell clones persist after treatment

To investigate T-cell clone persistence, a second muscle biopsy was performed in patient 4 (ASyS) and patient 2 (IMNM) after 9 months of immunosuppressive treatment. In both cases, after treatment, laboratory markers (C-reactive protein (CRP), erythrocyte sedimentation rate (ESR) and creatine-kinase (CK)) had normalized (Dataset EV1). Muscle strength had improved for patient 2 (IMNM) and remained unchanged for patient 4 (ASyS). Even though no immune infiltrates were detected in muscle biopsies by histopathology after treatment, T cells could be single-cell sorted (Dataset EV1, Appendix Fig S1). Unsupervised analysis of all seven samples taken at early diagnosis, combined with the two patient samples taken after treatment revealed nine clusters in muscle and eight clusters in blood (Fig EV5A and B) very similar to the previous clustering (Figs 1C and 2A) apart from $HLADR^+$ T_{RM} and $HOBIT^+$ T_{RM} clusters which were fused into one cluster (cluster 5, HLA-DR/$HOBIT^+$ T_{RM}) in muscle and the $GZMB^+$ EM cluster in PB that was split into two clusters (cluster 1A-$GZMB^+$ EM and cluster 1B-$GZMB^+$ EM). In both patients, the frequency of HLA-DR/$HOBIT^+$ T_{RM} cells in the muscle (cluster 5) was lower after treatment (Fig EV5C–F). In parallel, the frequency of muscle $GZMB^+$ EM (cluster 2) T cells increased after treatment. In blood, NK-like $CD8^+$ T cells (cluster 6) were not detected after treatment and the frequency of PB $GZMB^+$ EM (clusters 1A, 1B) T cells increased (Fig EV5G–J). T-cell clone frequencies in muscle and blood after treatment were very similar as before treatment (Fig 5A–D). Expanded T-cell clones represented around 55% of all CDR3 sequences in muscle and 25% in PB in the re-biopsy of patient 2 (IMNM; Fig 5A), whereas it ranged around 30% in the muscle and around 65% in PB in patient 4 (ASyS; Fig 5C). Analysis of these CDR3 sequences showed that the most expanded clones in muscle or PB at the time of diagnosis persisted after treatment (Fig 5B and D). In patient 2 (IMNM), most of the muscle expanded T-cell clones at diagnosis and after treatment originated from $GZMB^+$ EM $CD8^+$ T cells (cluster 2) and $EGR1^+$ T_{RM} (cluster 4; Fig 5E, left panel, Datasets EV9 and EV10). In patient 4 (ASyS), the expanded cytotoxic $CD4^+$ T-cell clone observed in the muscle (cluster 2) remained cytotoxic at re-biopsy but fewer HLA-DR/$HOBIT^+$ T_{RM} (cluster 5) were detected after treatment (Fig 5F left panel, Dataset EV10). In both patients, most expanded T cells in PB were identified within $GZMB^+$ EM $CD8^+$ or $CD4^+$ T cells before and after treatment (clusters 1A, 1B; Fig 5E and F, right panels, Datasets EV9 and EV10). These data indicate that expanded T-cell clones remain in the muscle after conventional immunosuppressive treatment.

Differentially expressed genes including a type 1 interferon (IFN) signature are revealed in distinct IIM subgroups

We then investigated differential gene expression in muscle T cells among the IIM subgroups. We identified type 1 IFN related genes

Figure 4. Expanded T-cell clones are identified in the muscle and corresponding memory peripheral blood T cells in patients with idiopathic inflammatory myopathies.

A Stacked bar plots showing the frequency of T-cell clones per patient in the muscle (left) and peripheral blood (PB) (right), including non-expanded (unique, lightest shade) and expanded clones (colored from light to dark shades, from less expanded to more expanded clones).

B Network plots displaying T-cell expanded clones per patient, in both muscle (circle) and PB (square). The color of the shape corresponds to the T-cell cluster as indicated. Dark brown indicates $CD4^+$ T cells, light brown indicates $CD8^+$ T cells, grey indicates $CD8^-CD4^-$ T cells. The connecting line between at least two cells indicates sharing of CDR3 sequences. n indicates the number of expanded T cell-clones per patient.

Data information: CM: central memory; EM: effector memory; TRM: tissue resident memory. IMNM: Immune-Mediated Necrotizing Myopathy; DM: DermatoMyositis; ASyS: AntiSYnthetase Syndrome; IBM: Inclusion Body Myositis; IIM: Idiopathic Inflammatory Myopathies.
Source data are available online for this figure.

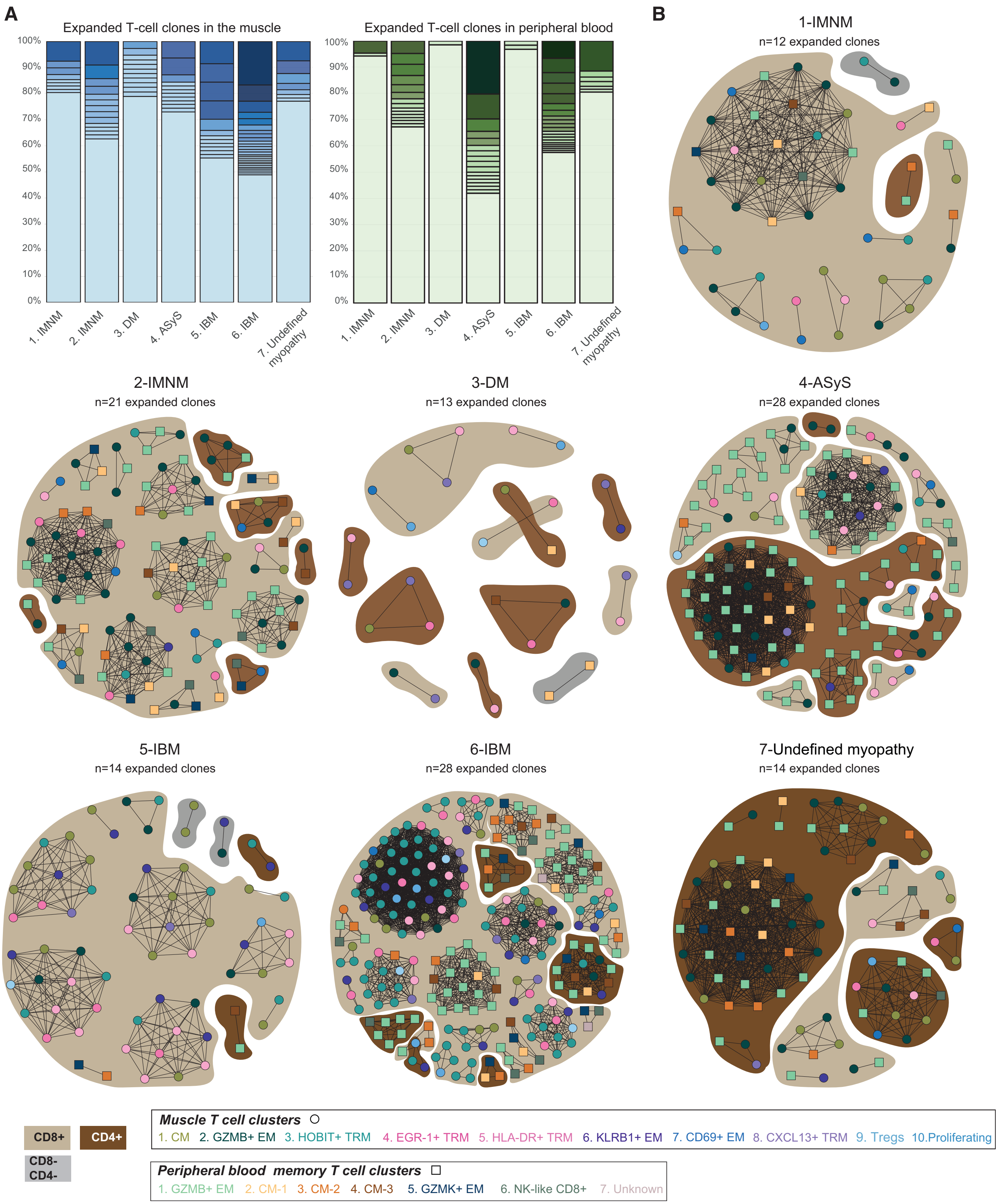

Figure 4.

© 2023 The Authors

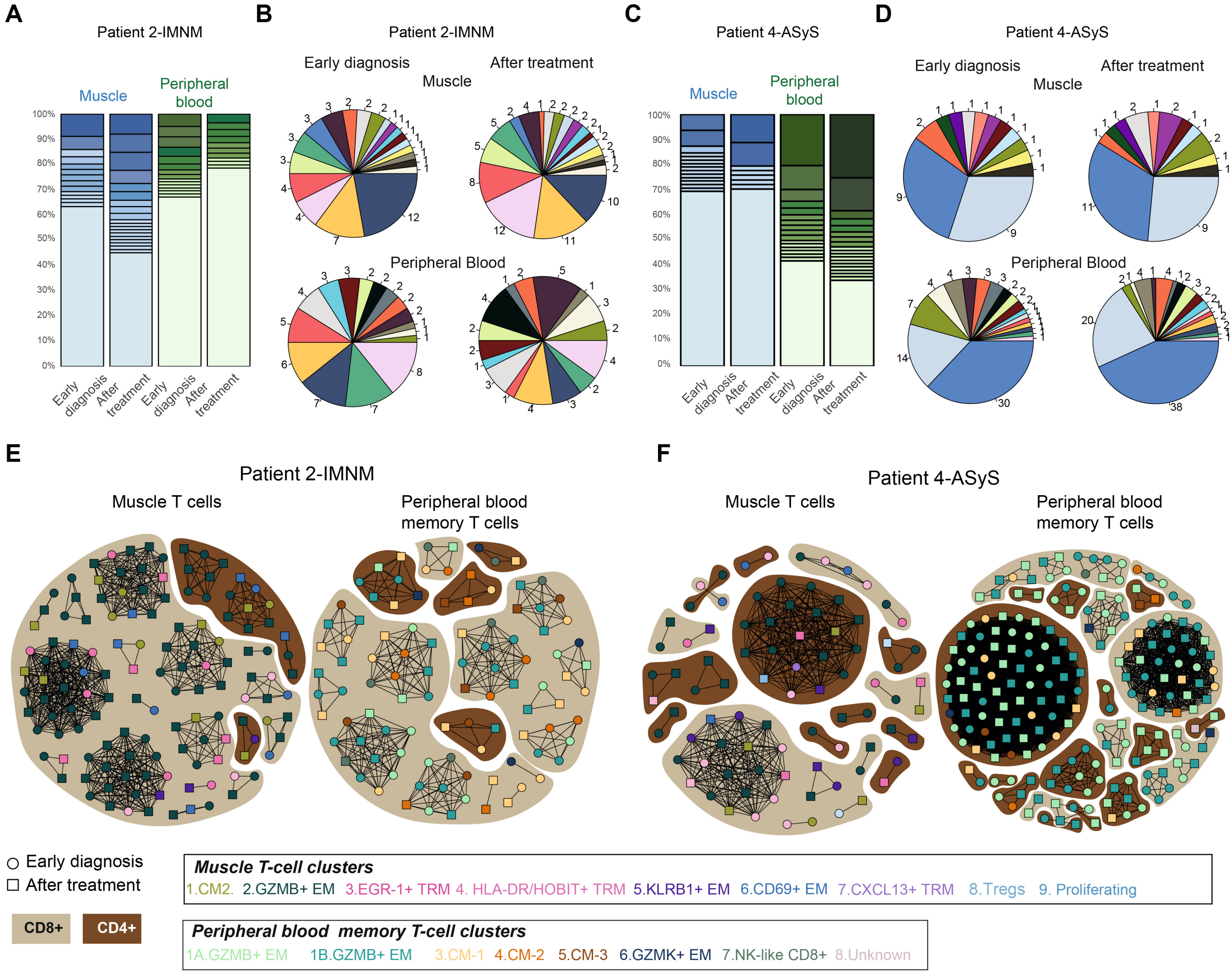

Figure 5. T-cell clones persist after treatment in two patients with idiopathic inflammatory myopathies.

A Stacked bar plots displaying the frequency of T-cell clones in patient 2 (IMNM) at early diagnosis and after treatment in muscle and peripheral blood (PB).

B Pie charts showing the shared CDR3 sequences (expanded or unique) between early diagnosis (left panel) and after treatment (right panel) in muscle (upper panel) or PB (lower panel) in patient 2 (IMNM). The same color shows identical CDR3 sequences. Numbers indicate number of cells per CDR3 sequence.

C Stacked bar plots displaying the frequency of T-cell clones in patient 4 (ASyS) at early diagnosis and after treatment in muscle and PB.

D Pie charts showing shared CDR3 sequences (expanded or unique) between early diagnosis (left panel) and after treatment (right panel) in muscle (upper panel) or PB (lower panel) in patient 4 (ASyS). The same color shows identical CDR3 sequence. Numbers indicate number of cells per clone.

E Network plots displaying shared and/or expanded CDR3 sequences in patient 2 (IMNM), in muscle (left) and PB (right) at early diagnosis (circle) and after immunosuppressive treatment (square).

F Network plots displaying shared and/or expanded CDR3 sequences in patient 4 (ASyS), in muscle (left) and PB (right) at early diagnosis (circle) and after immunosuppressive treatment (square).

Data information: (A, C) Stacked bar plots where lightest color shows unique sequences; the expanded clones are colored from light to dark shades, from less expanded to more expanded clones. (E-F) The color of the shape corresponds to the T-cell cluster as indicated.
Dark brown indicates $CD4^+$ T cells, light brown indicates $CD8^+$ T cells.
The connecting line between at least two cells indicates sharing of CDR3 sequences. CM: central memory; EM: effector memory; TRM: tissue resident memory. IMNM: Immune-Mediated Necrotizing Myopathy; DM: DermatoMyositis; ASyS: AntiSYnthetase Syndrome; IBM: Inclusion Body Myositis; IIM: Idiopathic Inflammatory Myopathies.
Source data are available online for this figure.

(*IFI44L*, *IFI6*) in muscle T cells in the patient with ASyS and to some extent in the patient with DM but not in the other IIM patients (Fig 6A, Dataset EV11). Cytotoxicity-related genes (*FGFBP2*, *FCGR3A* encoding CD16) were identified in IMNM patients 1 and 2 and the undefined myopathy patient 7. In addition, muscle T cells from the patient 7 with undefined myopathy expressed increased levels of *SELL*, *TNFRSF25* and *LEF1* suggestive of an enrichment for recirculating central memory T cells as seen in Fig 1F. Muscle T cells from

IBM patients differentiated from the others by a stronger expression of genes related with T-cell tissue residency (*ITGAE*, *XCL1*, *CXCR6*, *ZNF683*), whereas in the patient with DM, muscle T cells presented an increased expression of *CXCL13*, *HLA-DRB5* and *HLA-DRB1* possibly reflecting the presence of peripheral helper T cells (Kobayashi *et al*, 2013; Rao *et al*, 2017; Argyriou *et al*, 2022). The expression of

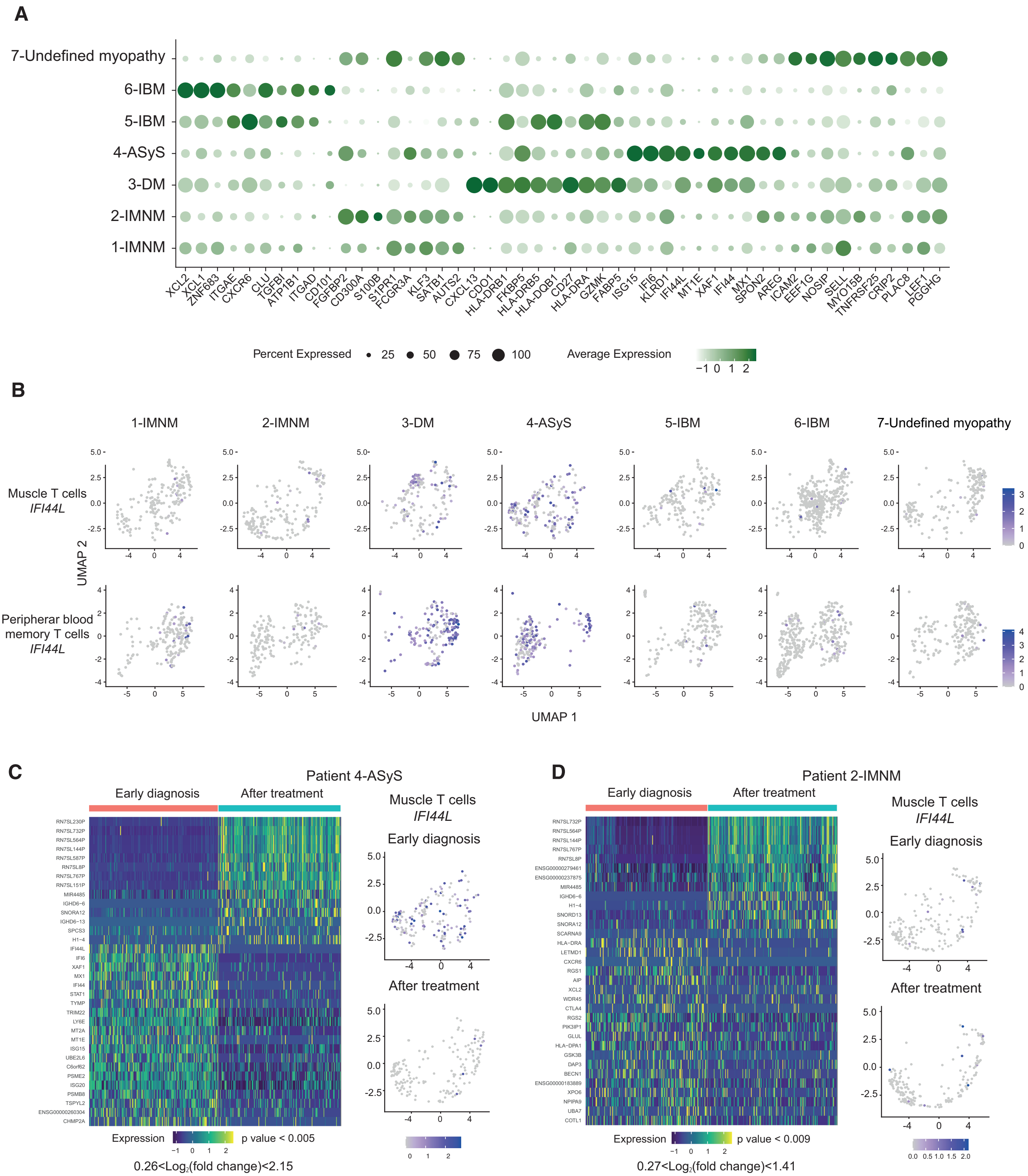

Figure 6.

© 2023 The Authors

Figure 6. Differentially expressed genes in distinct idiopathic inflammatory myopathies subgroups.

A Dot plot displaying genes selected from the top 10 differentially expressed genes per subgroup of disease in muscle T cells. Circle size indicates the percentage of cells expressing, color intensity indicates average expression.

B Feature plots of *IFI44L* expression levels in each patient at early diagnosis in muscle T cells (upper panel) and peripheral blood memory T cells (lower panel).

C Left panel: Heatmap showing the normalized and scaled expression of the 10 most upregulated genes and the 10 most downregulated genes (based on average log2FC) in T cells when comparing gene expression at early diagnosis versus gene expression after treatment in the muscle of patient 4 (ASyS) ($0.26 < \log_2$ fold change < 2.15, P value < 0.005). Right panel: UMAP feature plot displaying *IFI44L* gene expression at early diagnosis (upper) and after treatment (lower) in muscle T cells.

D Left panel: Heatmap showing the normalized and scaled expression of the 10 most upregulated genes and the 10 most downregulated genes (based on average log2FC) in T cells when comparing gene expression at early diagnosis versus gene expression after treatment in the muscle of patient 2 (IMNM) ($0.27 < \log_2$ fold change < 1.41, P value < 0.009). Right panel: UMAP feature plot displaying *IFI44L* gene expression at early diagnosis (upper) and after treatment (lower) in muscle T cells.

Data information: IMNM: Immune-Mediated Necrotizing Myopathy; DM: DermatoMyositis; ASyS: AntiSYnthetase Syndrome; IBM: Inclusion Body Myositis; IIM: Idiopathic Inflammatory Myopathies.

killer cell lectin-like receptor G1 (KLRG1) has previously been identified on cytotoxic T cells in IBM (Greenberg *et al*, 2019). Indeed, *KLRG1* was detected on GZMB$^+$ effector T cells presenting cytotoxic features (*GZMB*, *PRF1*, *ADGRG1*) but also to a lesser extent on T_{RM} cells (Fig EV1B). The expression of *KLRG1* was not restricted to IBM but was also detected in T cells in the muscle of the other IIM subgroups (Fig EV1D). We observed that only PB and muscle T cells from patient 3 (DM) and patient 4 (ASyS) had an increased type 1 interferon profile at early stages of the disease in comparison with the other IIM subgroups (Fig 6B) or to memory T cells from PB of healthy donors (Fig EV3H and I). Interestingly, muscle T cells from patient 4 (ASyS) presented a downregulation of type 1 interferon related genes (*IFI44L*, *IF6*, *IFI44*) after treatment (Fig 6C, Dataset EV12) which was not observed in patient 2 (IMNM; Fig 6D, Dataset EV13). These results point to some distinct signatures in muscle T cells from IIM subgroups.

Discussion

By performing single-cell sequencing of infiltrating T cells in muscle from patients with IIM, we identified effector, tissue resident, Tregs and proliferating T cells. Our study unraveled the presence of four T-cell clusters expressing genes associated with tissue resident memory. Most of the clonally expanded muscle T cells were identified within the GZMB$^+$ effector and T_{RM} cells. Identical clones were detected in PB and muscle T cells in repeated biopsies from two patients after 9 months of treatment suggesting resistance to immunosuppressive treatment. Our study provides a unique unbiased transcriptomic and TCR landscape of muscle T cells in IIM which might be used to design new treatment strategies and possible prognostic markers.

The presence of immune cell infiltrates is one of the important signs to support the diagnosis and classification of IIM (Dorph *et al*, 2001) and T cells are proposed to play an active role in muscle fiber damage (Goebels *et al*, 1996; Sugiura *et al*, 1999; Pandya *et al*, 2010; Greenberg *et al*, 2019). Single-cell sequencing offers the possibility to deeply investigate T-cell effector functions in the context of their clonality. We observed that, despite clinical heterogeneity, most expanded muscle T-cell clones were present among CD8$^+$ T cells. Indeed, even though CD4$^+$ T cells were detected in all biopsies, only the patient with ASyS presented with clonally expanded muscle cytotoxic CD4$^+$ T cells, which indicates differences in molecular pathways between IIM subgroups. Infiltrates of highly cytotoxic CD8$^+$ T cells have been described in muscle biopsies of IBM patient for decades (Arahata & Engel, 1984, 1986; Pandya *et al*, 2010; Greenberg *et al*, 2019) and their resistance to treatment may explain the lack of therapeutic response in these patients. In line with these data, we identified a cluster of GZMB$^+$ effector T cells in the muscle of all patients. We confirmed *KLRG1* expression on GZMB$^+$ effector T cells as previously described (Greenberg *et al*, 2019) but this expression was not restricted to the IBM subset. Nevertheless, in the two IBM patients included in this study, clonally expanded CD8$^+$ T cells displayed a tissue resident memory phenotype including the expression of *HOBIT*, *CXCR6*, *XCL1*, and *XCL2*. We also detected *GZMK* in three T_{RM} subsets confirming a previous report describing GZMK in T_{RM} CD8$^+$ cells in the synovium of RA patients (Jonsson *et al*, 2022). In blood, NK-like CD8$^+$ T cells with high cytotoxic features were detected in all patients at different frequencies. These cells shared TCR sequences with GZMB$^+$ effector T cells suggesting that they represent different states of differentiation. NK-like CD8$^+$ T cells have been described in PB in autoimmune diseases such as lupus (Basu *et al*, 2009) and celiac disease (Meresse *et al*, 2006) but also in severe acute respiratory syndrome coronavirus 2 (Li *et al*, 2022) where they are proposed to be the human equivalent of the immunoregulatory Ly49$^+$CD8$^+$ T cells (Li *et al*, 2022). Sharing of TCR sequences between effector/cytotoxic T cells and T_{RM} cells suggests that these cell types might also be developmentally connected. The persistence of clones despite conventional immunosuppressive treatment could be interpreted as a failure to achieve remission and could explain the development of flares after treatment tapering or suspension. These tissue resident features might explain chronicity and difficulties in treating patients with IBM.

Biopsies from patients with IMNM are usually characterized by more limited presence or even complete absence of T-cell infiltrates (Merlonghi *et al*, 2022). However, CD3$^+$ T cells densities in muscle biopsies from anti-SRP$^+$ and anti-HMGCR$^+$ patients were shown to be the same as in anti-Jo1$^+$ patients (Allenbach *et al*, 2018), highlighting the possibility to further study these cells from muscle biopsies. Here, we could detect clonally expanded effector GZMB$^+$ CD8$^+$ T cells in the muscle of the two IMNM patients. These clones were shared with GZMB$^+$ effector T cells in PB suggesting circulation between PB and muscle. After treatment, no immune cell infiltrates were identified in the muscle biopsies of patients with IMNM and ASyS by histopathology, but muscle T cells could still be single-cell sorted and sequenced. Single-cell sorting and sequencing is

therefore a sensitive method to detect phenotypical changes in muscle immune cell infiltrates. T-cell clonality analysis in two patients after 9 months with conventional immunosuppressive treatment revealed the persistence of T-cell clones which were also detected in PB, despite clinical improvement. We detected a decrease in the expression of type 1 IFN-related genes in the ASyS patient after treatment, which was associated with clinical response, indicating a possible role of type I IFN pathway in this subset of IIM. In a patient with DM, a *CXCL13* signature was detected which might indicate the presence of peripheral helper T cells as described in RA (Kobayashi *et al*, 2013; Rao *et al*, 2017; Argyriou *et al*, 2022). We identified shared and patient-specific signatures that suggest the involvement of different molecular mechanisms in IIM subgroups.

Regulatory T cells were identified in the skeletal muscle of mice following acute injury (Burzyn *et al*, 2013) and in a model of muscular dystrophy (Villalta *et al*, 2014). In these models, Tregs are proposed to promote muscle repair through the growth factor amphiregulin (Burzyn *et al*, 2013). FOXP3$^+$ Tregs have also been found in muscle biopsies of patients with IIM where they potentially could neutralize CD8$^+$ cytolytic effector functions (Waschbisch *et al*, 2010). Here, we identified *FOXP3*$^+$ Treg cells expressing *TIGIT*, *TNFRSF9* and *ICOS* but also a tissue resident memory signature suggesting their maintenance within the tissue. *FOXP3*$^+$ Tregs were detected in all patients and did not present signs of clonal expansion possibly due to the small number of cells detected. Our data show that in the muscle of patients with IIM, *FOXP3*$^+$ Tregs are outnumbered by effector T cells probably making Tregs unable to control local inflammation.

One limitation of this study is the sample size due to the rarity of IIM. Hence, the transcriptomic signature observed in the different IIM subgroups needs to be confirmed in further studies. Single-cell sorting and sequencing of muscle infiltrating T cells could be performed in seven out of the 15 patients with suspicion of myositis. However, the sometimes patchy distribution of inflammatory infiltrates in the muscle tissue, the lack of immune infiltrates, or a predominance of other immune cells such as macrophages might account for the fact that we did not detect T cells by flow cytometry in muscle biopsies from all patients with IIM. Here, we have chosen the Smart-seq2 sequencing technology because it has a good transcriptome coverage (30–40%) and allows the recovery of TCRαβ sequences from the same dataset (Nguyen *et al*, 2018). A disadvantage of this method is the limited number of cells which can be single-cell sequenced. Another limitation of this study is the lack of comparison with muscle-infiltrating T cells from healthy donor's biopsies. Such experiments, although technically challenging given the low numbers of T cells infiltrating the healthy muscle tissues (Rubenstein *et al*, 2020) will inform about possible differences in the profile of T_{RM} cells at the steady state compared to IIM.

In conclusion, our data demonstrate the feasibility and sensitivity of using single-cell sequencing to explore the T-cell infiltrate diversity in muscle tissue from patients with IIM, even in cases where few immune cell infiltrates are detected by histopathology. We identified receptors associated with homing and tissue-residency. Expanded T-cell clones were found within effector and tissue resident T-cell clusters and persisted after conventional immunosuppressive treatment. The comparison with memory T cells in PB revealed distinct T-cell phenotypes in the muscle tissue, emphasizing the importance of investigating the muscle tissue in these diseases. Our study provides an immunoprofiling T-cell map to understand the pathogenesis and to facilitate the development of novel therapies in myositis.

Materials and Methods

Patients and healthy controls

Muscle biopsies and PB from 15 patients with clinical signs of muscle weakness were collected at time of diagnosis at the Karolinska Hospital Rheumatology Clinic (Dataset EV1). Classification of IIM was done according to the 2017 European League Against Rheumatism/American College of Rheumatology classification criteria (Lundberg *et al*, 2017). Out of 15 patients, one turned out to have an undefined myopathy, and another patient was diagnosed with Becker muscular dystrophy based on whole exome sequencing. No sample size calculation was made. In six patients with IIM and one patient with undefined myopathy (median age = 62, 43% female), clear immune cell infiltrates in muscle tissue were confirmed by flow cytometry and histopathology. T cells were subsequently single-cell sorted from the fresh muscle biopsy and from corresponding PB of these seven patients (Dataset EV1 and Appendix Fig S1). In three healthy donors (median age = 59, 100% female), memory T cells were single-cell sorted from PB (Fig EV3A–E). The local research ethics committee at Karolinska University Hospital (Dnr 2020-06900) and Stockholm region (Dnr 2010/935-31/1) approved the study. All participants signed an informed consent and the experiments were conducted according to the principles set out in the World Medical Association Declaration of Helsinki and the Department of Health and Human Services Belmont Report.

Muscle biopsy

Muscle biopsies were obtained with a "semi-open" muscle biopsy technique (Henriksson, 1979). After local anesthesia, an alligator forceps (Weil-Blakesley's conchotome) was used to collect muscle biopsies by an experienced rheumatologist from either vastus lateral or tibialis anterior muscle (Dataset EV1). Muscle biopsies underwent routine histochemical and immunohistochemical stains for inflammatory cells and were distinguished from non-autoimmune myopathies by an experienced muscle pathologist.

Autoantibody testing

Autoantibodies against Jo1, PL12, PL7, OJ, EJ, Mi2, MDA5, NXP2, TIF1γ, SAE1, SRP, HMGCR, PMScl, Ro52, and U1RNP were analyzed using one or more of the following: (i) immunoprecipitation, (ii) Euroline myositis panel 4 by Euroimmun, Lübeck, Germany, and/or (iii) enzyme-linked immunosorbent assays (ELISA; Dataset EV1).

Single-cell processing

Fresh muscle biopsies were digested with liberase (Roche, 0.25 mg/ml) or collagenase A (Sigma Aldrich, 4 mg/ml) and DNAse I (Sigma Aldrich, 0.5 mg/ml) during 60 min at 37°C and stained with near

 © 2023 The Authors

infra-red viability dye (Invitrogen, Catalog #L10119) and fluorescently coupled antibodies (gating strategy in Appendix Fig S2). Anti-CD45 and anti-CD3 antibodies (Appendix Table S1) were used to enrich for T cells within the muscle tissue and to minimize sorting of cells/debris which could bind not specifically to anti-CD3 antibodies. Of note, the anti-CD45 monoclonal antibody (clone HI30, BD biosciences) binds to the 180 (CD45RO), 190, 205, 220 (CD45RA) kDa protein isoforms of CD45. One hundred and ninety-two $CD45^{+}CD3^{+}$ cells were single-cell sorted using an influx sorter (BD Biosciences). One hundred and ninety corresponding $CD3^{+}$ memory T cells (by excluding naive $CCR7^{+}CD45RA^{+}$ T cells) from PB were sorted in the same plate to minimize batch effects. We sorted memory T cells from PB to maximize the possibility to identify expanded T cell clones and to reduce sequencing costs. For patient 6, we could sort 382 $CD45^{+}CD3^{+}$ T cells from the muscle and 382 $CD3^{+}$ memory T cells from PB. To evaluate the effect of collagenase on gene expression (Fig EV3F), 1.10^{6} PBMC were incubated with collagenase A (Sigma Aldrich, 4 mg/ml) and DNAse I (Sigma Aldrich, 0.5 mg/ml) during 60 min at 37°C using the same protocol as for muscle biopsies. Single T cells from IIM (muscle and PB) were submitted to Smart-seq2 sequencing. Single T cells from healthy donors were run at a different time point and submitted to Smart-seq3 sequencing (Fig EV3A–E).

Smart-seq2/3 single-cell RNA library preparation and sequencing

For IIM patients, single-cell RNA-sequencing libraries were generated with the Smart-seq2 protocol based on the original publication (Picelli *et al*, 2013) at the Eukaryotic Single-cell Genomics facility at Science for Life Laboratory in Stockholm, Sweden. T cells were single sorted in individual wells of 384 well-plates containing 2.3 µl lysis buffer, which were provided by the Eukaryotic Single-Cell Genomics (ESCG) Facility at SciLifeLab, Stockholm. Index cell sorting was done with a BD Biosciences Influx cell sorter within a 20-min range time at the Flow cytometry Core, Center for Molecular Medicine (CMM, Karolinska Institutet (KI), Stockholm). The plate was spun down and kept on dry ice until its temporary storage at −80°C. Each well contained 2.3 µl of lysis buffer (1.15 µl of 0.4% Triton X-100 (Sigma-Aldrich), 0.4 µl of dNTPs mix (25 mM, Thermo Fischer Scientific), 0.05 µl of Smart-dT_{30}VN (100 µM, IDT DNA Technology), 0.05 µl of RNAse inhibitor (40 U/µl, Takara), 0.0025 µl of ERCC (1:40,000 dil, Ambion), 0.6475 µl of water, Thermo Fischer Scientific). The plates were then transferred to the ESCG Facility for library preparation and sequencing, following the next steps. The plates were incubated at 95°C for 3 min to lyse the cells. cDNA synthesis and preamplification steps were then performed. The plates were spun down, and 2.7 µl of reverse transcription (RT) mix 1 µl of SSRT II buffer (5×, Thermo Fischer Scientific), 0.25 µl of DTT (100 mM, Thermo Fischer Scientific), 1 µl of Betaine (5 M, Sigma), 0.04 µl of $MgCl_2$ (1 M, Ambion), 0.125 µl of RNAse inhibitor (40 U/µl, Takara), 0.25 µl of SSRT II (200 U/µl, Thermo Fischer Scientific), 0.05 µl of LNA-TSO (100 µM, Qiagen / Exiqon) were added to each well. The plates were spun down and submitted to the RT program in a thermocycler (90 min at 42°C, 15 min at 70°C and 4°C). Then, 7.5 µl of KAPA PCR mix (6.25 µl of KAPA HiFi HS RM (2×, Roche), 0.1 µl of IS PCR primers (10 µM, IDT DNA Technology), 1.15 µl of water, Thermo Fischer Scientific) were added to each well and incubated for PCR amplification (3 min at 98°C; 23 cycles: 30 s at 98°C, 20 s at 67°C; 6 min at 72°C; 5 min at 72°C; 4°C). The next steps included cDNA purification, quality control, and normalization. First, the bead-mix was prepared: 1 ml of Seramag Speedbeads (GE Healthcare #45152105050250) was washed in 1 ml of TE (Tris & EDTA) pH8, three times, and resuspended in 1 ml TE. Then, the beads were mixed with 49 ml of freshly prepared bead buffer (NaCl 1 M, Tris–HCl pH8.0 0.01 M, EDTA 0.001 M, PEG 8000 17%, Tween 20 0.01%, NaAz 0.05%). The cDNA was purified by adding 9 µl of beads suspension per well (0.7:1 ratio), mixed well, and incubated for 5 min. The plates were transferred to a magnet during 4 min to allow the beads to settle, and then the supernatant was removed and discarded. The plates were removed from the magnet, and the beads were resuspended in 14 µl of nuclease-free water and incubated for 2 min. The plates were transferred to a magnet for 2 min before transferring the supernatant to a new plate. Eleven random wells per plate were analyzed using the large fragment High Sensitivity DNA kit (Agilent) to evaluate the amounts of cDNA or traces of RNA degradation. This information was used to calculate the average DNA concentration of each plate. Nuclease-free water was added to reach a DNA concentration of 200 pg/µl per well. Then, the library was performed. 0.5 µl of DNA from each well was transferred to a new plate. 1.5 µl of tagmentation mix (1 µl of Tagment DNA buffer "TD" 2×, 0.5 µl of tagmentation enzyme-Amplicon Tagment mix -Illumina Nextera XT kit-) were added to each well. The oligos used in this step were identical to the Smart-seq2 protocol[52]. Smart-dT30VN: 5′-AAGCAGTGGTATCAACGCAGAGTACTTTTTTTTTTTTTTTTTTTTTTTTTTTTTTVN-3′; LNA-TSO: 5′-AAGCAGTGGTATCAACGCAGAGTACrGrG+G-3′; IS PCR primers: 5′-AAGCAGTGGTATCAACGCAGAGT-3′. The plates were spun down and incubated for 8 min at 55°C. Then, 0.5 µl of neutralization buffer from the Nextera XT Kit was added to each well to stop the tagmentation and the plates were incubated at room temperature for 5 min. One microliter of indexes (N5xx + N7xx, Nextera XT kit. Illumina index primers i5 standard: 3′-CTGCGACGGCTGCTxxxxxxxxCACATCTAGAGCCACCAGCGGCATAGTAA-5′; i7 standard: 5′-CAAGCAGAAGACGGCATACGAGATxxxxxxxxGTCTCGTGGGCTCGG-3′, where x = barcode for each sample) and 1.5 µl of Nextera XT PCR Mastermix were added to each well to run the enrichment PCR program on a thermocycler (3 min at 72°C; 30 s at 95°C; 15 cycles: 10 s at 95°C, 30 s at 55°C, 30 s at 72°C; 5 min at 72°C;10°C). Then, library purification and quality control were performed. The content of each well was pooled in a tube. The bead-mix was prepared as previously: 1 ml of Seramag Speedbeads (GE healthcare #45152105050250) was washed in 1 ml of TE (Tris & EDTA) pH8, three times, and resuspended in 1 ml TE. The beads were mixed with 49 ml of freshly prepared bead buffer (NaCl 1 M, Tris–HCl pH8.0 0.01 M, EDTA 0.001 M, PEG 8000 17%, Tween 20 0.01%, NaAz 0.05%). Part of the pooled library was purified using a 1:1 ratio of beads, performing two ethanol (80% w/v ethanol) washes and eluting in nuclease-free water. Finally, the library pool was quantified using a qubit HS DNA kit, and a sample ran on a Bioanalyzer to check size distribution. Libraries were sequenced on an Illumina Nova seq 6000 (2*100 bp) at NGI Stockholm sequencing core facilities, Science for Life Laboratory in Stockholm, Sweden. For peripheral memory T cells from healthy donors, single-cell RNA-sequencing libraries were generated with the Smart-seq3 protocol, based on the original publication (Hagemann-Jensen *et al*, 2020;

© 2023 The Authors

and available online: https://www.protocols.io/view/smart-seq3-protocol-36wgq5rjxgk5/v3?step=17) at the Eukaryotic Single-cell Genomics facility at Science for Life Laboratory in Stockholm, Sweden. As for the Smart-seq2 protocol, memory T cells were single sorted in individual wells of 384 well-plates containing 3 μl lysis buffer (see details in the link), provided by the ESCG Facility at SciLifeLab, Stockholm. Index cell sorting was done with a BD Biosciences Influx cell sorter within a 20-min range time at the Flow cytometry Core, CMM, KI. The plate was spun down and kept on dry ice until its temporary storage at −80°C. The plates were then transferred to the ESCG Facility for library preparation and sequencing following the steps described in https://www.protocols.io/view/smart-seq3-protocol-36wgq5rjxgk5/v3?step=2, with the exception that in step 6 (cDNA amplification), the elongation time was 6 min instead of 4 min. The number of amplification cycles was 21. The library quantification was performed in a plate reader with picogreen. Libraries were sequenced on an Illumina Nova seq 6000 (2*100 bp) at NGI Stockholm sequencing core facilities, Science for Life Laboratory in Stockholm, Sweden (Appendix Fig S3 and Fig EV3A).

Data processing and quality control steps

Appendix Fig S3 summarizes the data analysis workflow. After Smart-seq2 sequencing, binary base-call files (BCL) were converted to fastq files and demultiplexed (to obtain pairs of fastq files from the pair-sequencing per cell) using the bcl2fastq v2.20 software from Illumina at the Eukaryotic Single-Cell Genomics (ESCG) facility. The indexes of the Smart-seq2 reads were trimmed using Trim-Galore v0.4.4. Reads were mapped to the human genome assembly hg38 plus exon-exon junctions from gencode v21 (--sjdbGTFfile) using STAR v2.5.4b. The gene expression was then calculated in Reads Per Kilobase of transcript, per Million mapped reads (RPKM) based on the RefSeq gene annotation (from the UCSC genome browser 10 Sep 2017) using the rpkmforgenes.py script (13 Mar 2015 version; https://gist.github.com/radaniba/4170501) with settings -minqual 255 -rmnameoverlap -fulltranscript. A size factor was calculated using the R package scran (Lun *et al*, 2016) v1.8.2, with a clustering size of 30 cells: cells expressing very few genes (with a negative size factor) were excluded. For Smart-seq3 sequencing, the gene expression matrix in raw counts was then generated using the zUMIs (Parekh *et al*, 2018) by the ESCG facility (Fig EV3A). The quality control and the biological interpretation analyses were performed in R (v4.2.1) with the Seurat package v4.3.0 (Satija *et al*, 2015). Cells expressing (i) less than 1,000 genes, (ii) more than 12,000 genes, (iii) <600,000 RNA molecules, or (iv) more than 8,000,000 RNA molecules were filtered out. Cells with mitochondrial genes representing more than 35% of the whole cell genome were excluded. To remove non-T cells, cells expressing low levels of *CD3E*, *CD3D*, or *CD3G* (< *5RPKM*) were excluded. Cells expressing *CD14*, *CD19*, *CD22* or *CD300E* (> 5 RPKM) and *HBA1* & *HBA2* (> 2,500) were filtered out. *Alpha*, *beta*, *gamma*, and *delta* T-cell receptor genes were filtered out to not introduce a bias in the clustering. After all filtering steps, the quality of the transcriptomics data per donor was visualized by plotting (i) the total number of genes, (ii) the number of transcripts (ii), the percentage of mitochondrial genes (iii) per donor and per compartment (Figs EV1A and EV2A). To reduce the technical noise while improving the detection of immune-related genes, we excluded ribosomal genes from the analysis. Feature counts for each cell were divided by the total counts for that cell and multiplied by a scale factor of 10,000, then normalized with a natural logarithm (NormalizeData function from Seurat with default parameters). Principal component analysis was performed for both PB and muscle separately, and the elbow plots were used to select the number of principal components included in the subsequent cluster analysis. Using these principal components, we integrated the data by correcting for sequencing batch effects using harmony package (Korsunsky *et al*, 2019; version 0.1.0). The data was analyzed by groups based on tissue and treatment status

Cluster analyses

Clusters were identified using the first 8 (muscle) and 7 (peripheral blood) principal components with the Louvain algorithm, implemented in the FindClusters function from Seurat Packages. Clustering was visualized using uniform manifold (UMAP) method. The differential gene expression was calculated between a given cluster and the rest of the cells using *a Wilcoxon Rank Sum* test implemented in the FindAllMarkers function from Seurat package, to explore genes that contribute to the cluster formation. Clusters were named manually based on known differentially expressed genes. For the heatmaps and dot plots, the top 5–10 differentially expressed genes based on average log2FC values were displayed. Gene set enrichment analysis (GSEA) was done using fgsea package (v1.24.0) from Bioconductor. Tissue resident $CD8^+$ T-cell signature and non-tissue resident $CD8^+$ T-cell signature gene sets for GSEA analysis were obtained from available datasets in the Gene Expression Omnibus (GEO) with the identifier GSE94964 (Kumar *et al*, 2017).

T-cell receptor analysis

T-cell receptor sequences were assembled using TraCeR v0.5.1 (Stubbington *et al*, 2016). A clone was defined by at least two cells sharing the same productive CDR3 amino acid sequence from both TCRα and TCRβ chains. Cells expressing two *alpha* and two *beta* chains were considered doublets and removed from the analysis. TCR V(D)J sequences were added into the single-cell Seurat object as metadata. The network analysis (Figs 4 and 5) was performed using Python 3.9 and the visualization using the pyNetworkPlot package (https://github.com/BioinfoVisualization/pyNetworkPlot).

Immunofluorescence and immunohistochemistry stainings

Muscle biopsies were sectioned and embedded on slides at the histocore facility, Karolinska Institutet (KI). Stainings were performed on muscle tissue from patients 3 (DM), 4 (ASyS), 6 (IBM; Dataset EV1) and 16 (IBM; Appendix Table S2) using monoclonal mouse anti-human CD3 (clone SK7, BD Biosciences, 10 ng/ml), polyclonal rabbit anti-human HOBIT (ThermoFischer, Catalog # PA5-54902, 6 ng/ml), mouse IgG1 isotype control (DAKO, Catalog # X0931, 10 ng/ml) and rabbit IgG1 isotype control (Invitrogen, Catalog # 02-6102, 6 ng/ml; Appendix Table S1). After overnight incubation at room temperature, slides were incubated with Alexa Fluor 488-conjugated goat anti-mouse (Invitrogen, Catalog # A-11029, 1:400), Alexa Fluor 647-conjugated goat anti-rabbit (Cell signaling, Catalog #4414,

© 2023 The Authors

The paper explained

Problem
Idiopathic inflammatory myopathies (IIM) are rare autoimmune systemic diseases that primarily affect the skeletal muscle. Importantly, T-cell infiltrates are often detected in the muscle of patients with IIM where they are suspected to contribute to tissue damage. However, the mechanisms involved in T-cell infiltration and persistence in the muscle during disease pathogenesis are still unclear. Current treatment approaches are limited, and patients often relapse. A deeper understanding of the pathogenic mechanisms leading to T-cell accumulation in the muscle is needed to envision the development of novel treatment approaches for patients with IIM.

Results
This study aims to map the immune profile of muscle-infiltrating T cells in patients with IIM using single-cell RNA sequencing. We identified effector, tissue resident, regulatory and proliferating T cells in the muscle of IIM patients. Moreover, T-cell receptor sequencing revealed expanded muscle T cells in the effector memory and tissue resident memory compartments, suggesting their maintenance in the tissue. Importantly, after conventional treatments, T-cell clones persisted in the muscle of patients where they might contribute to relapses.

Impact
This study shows that T cells in skeletal muscle of patients with IIM display tissue resident memory features suggesting their maintenance within the tissue and their probable contribution to relapses. The immunoprofiling map of muscle-infiltrating T cells can be used to understand the mechanisms leading to tissue damage and to identify novel therapeutic targets.

1:400) and Cy3-conjugated donkey anti-rabbit (Jackson Immunoresearch, Catalog # 711-165-152, 1:400) secondary antibodies and Hoechst 33342 DNA dye (ThermoFischer, Catalog #3570, 1:40,000). The muscle sarcolemma was stained using an Alexa Fluor 647-conjugated rabbit anti-human dystrophin antibody (Abcam, Catalog # ab282171, 1:100) during 1 h at room temperature. Images were acquired using LSM 880 confocal microscope without Airyscan. Images were processed and analyzed with the Fiji software. For the quantification of HOBIT$^+$ T cells (Fig 3G), sections were prepared and analyzed by two different persons in a blinded manner. During image acquisition, microscope fields were selected based on the presence of CD3$^+$ infiltrates. Cells were considered positive when a nuclear HOBIT staining overlapping with a Hoechst staining and surrounded by a CD3 staining was detected. Immunohistochemistry stainings were performed on 2% formaldehyde fixed muscle tissue using a standard avidin-biotin-peroxidase complex technique with a protocol adapted from (Tang *et al*, 2020). Briefly, fixed sections were washed with 0.1% saponin in 1× PBS (PBS-Sap) for 10 min, and then, a blocking step was performed with 1% H_2O_2 for 1 h at room temperature. After a washing step with PBS-Sap, endogenous biotin was blocked using an avidin-biotin blocking kit (Vector Laboratories, catalog # SP-2001). Sections were incubated overnight at room temperature with a polyclonal rabbit anti-human HOBIT antibody (ThermoFischer, Catalog # PA5-54902, 6 ng/ml) or rabbit IgG1 isotype control (Invitrogen, Catalog # 02-6102, 6 ng/ml) diluted in 3% normal human serum PBS-Sap. Next day, the sections were washed with PBS-Sap and blocked with 3% normal goat serum for 15 min. After blocking, biotinylated rabbit anti-goat IgG secondary antibody (H + L; Vector Laboratories, catalog # BA-5000-1.5) diluted in 1% normal goat serum, 3% normal human serum in PBS-Sap was incubated for 30 min. Sections were then washed with PBS-Sap and incubated with ABC Elite ABC-HRP kit (Vector Laboratories, catalog #PK-6102). Reactions were developed using a Peroxidase Substrate Kit (Vector Laboratories catalog #SK-4100) containing 3,3′-diaminobenzidine, with Mayer's hematoxylin counterstaining. Slides were mounted using PERTEX (Histolab, catalog# 00840-05). Images were acquired using a Leica Reichert Polyvar 2 light microscope (10× objective).

Data availability

Single-cell data access requests may be submitted to the Science for Life Laboratory Data Centre (datacentre@scilifelab.se) at https://doi.org/10.17044/scilifelab.21361029. All scripts used for data analysis are available on GitHub: https://github.com/scReumaKI/Myositis_scPipeline_2022.

Expanded View for this article is available online.

Acknowledgements
We thank the patients and the medical staff including research nurse Helene Sandlund at the Rheumatology Clinic of Karolinska University Hospital. Gloria Rostvall, Eva Lindroos, Julia Boström, and Susana Hernandez Machado are acknowledged for organizing the sampling, storage, and administration of biomaterial. We thank our patient research partner Anneli Dihkan for fruitful discussions. We thank Michael JT Stubbington for advice on TraCeR. We thank the scientific staff at the Flow Cytometry Core facility at the Center for Molecular Medicine at KI. We thank the scientific staff at the Eukaryotic Single-Cell Genomics (ESCG, Scilife, Stockholm). This study was founded by Olle Enqvist (KC, 222-0028), Dr. Margaretha Nilssons (KC), the Börje Dahlin (KC), the Åke Wiberg (LMDG, KC, M18-0167), the Nanna Svartz (KC, 2022-00466, LMDG, 2020-00377, BH, 2022-00470), the Ulla and Gustaf af Ugglas (KC, 2018-02627, LMDG, 2020-02395) foundations, the Swedish Rheumatism Association (KC, R-981415, LMDG, R-981752, IL), King Gustaf V 80 Year Foundation (KC, FAI-2022-0866, LMDG, FAI-2021-0837, IL), the KI Rheumatology fond (BH, 2022-02853), the Karolinska Institute (KC, 2022-01597), the Swedish Research Council (IL, 2020-01378), Region Stockholm (ALF project, IL, FoUI-955-086), Heart and lung foundation (IL, 20220127) and the Anna Hedin donation (IL). The authors acknowledge support from Science for Life Laboratory, the Knut and Alice Wallenberg Foundation, the National Genomics Infrastructure funded by the Swedish Research Council, and Uppsala Multidisciplinary Center for Advanced Computational Science for assistance with massively parallel sequencing and access to the UPPMAX computational infrastructure. Part of the computations and data handling were enabled by resources provided by the Swedish National Infrastructure for Computing (SNIC) at SNIC CENTRE partially funded by the Swedish Research Council through grant agreement no. 2018-05973.

Author contributions
Alexandra Argyriou: Conceptualization; data curation; formal analysis; validation; investigation; visualization; methodology; writing – original draft; writing – review and editing. **Begum Horuluoglu:** Conceptualization; data curation; formal analysis; funding acquisition; validation; investigation; visualization; methodology; writing – original draft; writing – review and

© 2023 The Authors

editing. **Angeles Shunashy Galindo-Feria:** Conceptualization; resources; data curation; investigation; visualization; methodology; writing – original draft. **Juan Sebastian Diaz-Boada:** Data curation; software; formal analysis; visualization. **Merel Sijbranda:** Validation; investigation. **Antonella Notarnicola:** Resources. **Lara Dani:** Resources. **Annika van Vollenhoven:** Investigation; methodology. **Daniel Ramsköld:** Software; methodology; writing – review and editing. **Inger Nennesmo:** Investigation. **Maryam Dastmalchi:** Resources; investigation. **Ingrid E Lundberg:** Resources; supervision; funding acquisition; writing – original draft; writing – review and editing. **Lina-Marcela Diaz-Gallo:** Conceptualization; data curation; formal analysis; supervision; funding acquisition; investigation; visualization; methodology; writing – original draft; project administration; writing – review and editing. **Karine Chemin:** Conceptualization; formal analysis; supervision; funding acquisition; investigation; visualization; methodology; writing – original draft; project administration; writing – review and editing.

Disclosure and competing interests statement

The authors declare that they have no conflict of interest.

References

Allenbach Y, Arouche-Delaperche L, Preusse C, Radbruch H, Butler-Browne G, Champtiaux N, Mariampillai K, Rigolet A, Hufnagl P, Zerbe N *et al* (2018) Necrosis in anti-SRP(+) and anti-HMGCR(+)myopathies: role of autoantibodies and complement. *Neurology* 90: e507 – e517

Arahata K, Engel AG (1984) Monoclonal antibody analysis of mononuclear cells in myopathies. I: quantitation of subsets according to diagnosis and sites of accumulation and demonstration and counts of muscle fibers invaded by T cells. *Ann Neurol* 16: 193 – 208

Arahata K, Engel AG (1986) Monoclonal antibody analysis of mononuclear cells in myopathies. III: immunoelectron microscopy aspects of cell-mediated muscle fiber injury. *Ann Neurol* 19: 112 – 125

Argyriou A, Wadsworth MH 2nd, Lendvai A, Christensen SM, Hensvold AH, Gerstner C, van Vollenhoven A, Kravarik K, Winkler A, Malmstrom V *et al* (2022) Single cell sequencing identifies clonally expanded synovial CD4(+) TPH cells expressing GPR56 in rheumatoid arthritis. *Nat Commun* 13: 4046

Barsotti S, Lundberg IE (2018) Current treatment for myositis. *Curr Treatm Opt Rheumatol* 4: 299 – 315

Basu D, Liu Y, Wu A, Yarlagadda S, Gorelik GJ, Kaplan MJ, Hewagama A, Hinderer RC, Strickland FM, Richardson BC (2009) Stimulatory and inhibitory killer Ig-like receptor molecules are expressed and functional on lupus T cells. *J Immunol* 183: 3481 – 3487

Bohan A, Peter JB (1975) Polymyositis and dermatomyositis (first of two parts). *N Engl J Med* 292: 344 – 347

Bottcher JP, Beyer M, Meissner F, Abdullah Z, Sander J, Hochst B, Eickhoff S, Rieckmann JC, Russo C, Bauer T *et al* (2015) Functional classification of memory CD8(+) T cells by CX3CR1 expression. *Nat Commun* 6: 8306

Burzyn D, Kuswanto W, Kolodin D, Shadrach JL, Cerletti M, Jang Y, Sefik E, Tan TG, Wagers AJ, Benoist C *et al* (2013) A special population of regulatory T cells potentiates muscle repair. *Cell* 155: 1282 – 1295

Cortez VS, Cervantes-Barragan L, Song C, Gilfillan S, McDonald KG, Tussiwand R, Edelson BT, Murakami Y, Murphy KM, Newberry RD *et al* (2014) CRTAM controls residency of gut $CD4^+CD8^+$ T cells in the steady state and maintenance of gut $CD4^+$ Th17 during parasitic infection. *J Exp Med* 211: 623 – 633

Dalakas MC, Sivakumar K (1996) The immunopathologic and inflammatory differences between dermatomyositis, polymyositis and sporadic inclusion body myositis. *Curr Opin Neurol* 9: 235 – 239

Dellabona P, Padovan E, Casorati G, Brockhaus M, Lanzavecchia A (1994) An invariant V alpha 24-J alpha Q/V beta 11 T cell receptor is expressed in all individuals by clonally expanded $CD4^-8^-$ T cells. *J Exp Med* 180: 1171 – 1176

Dobloug GC, Svensson J, Lundberg IE, Holmqvist M (2018) Mortality in idiopathic inflammatory myopathy: results from a Swedish nationwide population-based cohort study. *Ann Rheum Dis* 77: 40 – 47

Dorph C, Nennesmo I, Lundberg IE (2001) Percutaneous conchotome muscle biopsy. A useful diagnostic and assessment tool. *J Rheumatol* 28: 1591 – 1599

Furst DE, Amato AA, Iorga SR, Gajria K, Fernandes AW (2012) Epidemiology of adult idiopathic inflammatory myopathies in a U.S. managed care plan. *Muscle Nerve* 45: 676 – 683

Goebels N, Michaelis D, Engelhardt M, Huber S, Bender A, Pongratz D, Johnson MA, Wekerle H, Tschopp J, Jenne D *et al* (1996) Differential expression of perforin in muscle-infiltrating T cells in polymyositis and dermatomyositis. *J Clin Invest* 97: 2905 – 2910

Greenberg SA, Pinkus JL, Kong SW, Baecher-Allan C, Amato AA, Dorfman DM (2019) Highly differentiated cytotoxic T cells in inclusion body myositis. *Brain* 142: 2590 – 2604

Hagemann-Jensen M, Ziegenhain C, Chen P, Ramskold D, Hendriks GJ, Larsson AJM, Faridani OR, Sandberg R (2020) Single-cell RNA counting at allele and isoform resolution using Smart-seq3. *Nat Biotechnol* 38: 708 – 714

Henriksson KG (1979) "Semi-open" muscle biopsy technique. A simple outpatient procedure. *Acta Neurol Scand* 59: 317 – 323

Jonsson AH, Zhang F, Dunlap G, Gomez-Rivas E, Watts GFM, Faust HJ, Rupani KV, Mears JR, Meednu N, Wang R *et al* (2022) Granzyme K^+ CD8 T cells form a core population in inflamed human tissue. *Sci Transl Med* 14: eabo0686

Kobayashi S, Murata K, Shibuya H, Morita M, Ishikawa M, Furu M, Ito H, Ito J, Matsuda S, Watanabe T *et al* (2013) A distinct human $CD4^+$ T cell subset that secretes CXCL13 in rheumatoid synovium. *Arthritis Rheum* 65: 3063 – 3072

Korsunsky I, Millard N, Fan J, Slowikowski K, Zhang F, Wei K, Baglaenko Y, Brenner M, Loh PR, Raychaudhuri S (2019) Fast, sensitive and accurate integration of single-cell data with Harmony. *Nat Methods* 16: 1289 – 1296

Kumar BV, Ma W, Miron M, Granot T, Guyer RS, Carpenter DJ, Senda T, Sun X, Ho SH, Lerner H *et al* (2017) Human tissue-resident memory T cells are defined by core transcriptional and functional signatures in lymphoid and mucosal sites. *Cell Rep* 20: 2921 – 2934

Li J, Zaslavsky M, Su Y, Guo J, Sikora MJ, van Unen V, Christophersen A, Chiou SH, Chen L, Li J *et al* (2022) KIR(+)CD8(+) T cells suppress pathogenic T cells and are active in autoimmune diseases and COVID-19. *Science* 376: eabi9591

Love LA, Leff RL, Fraser DD, Targoff IN, Dalakas M, Plotz PH, Miller FW (1991) A new approach to the classification of idiopathic inflammatory myopathy: myositis-specific autoantibodies define useful homogeneous patient groups. *Medicine (Baltimore)* 70: 360 – 374

Lun AT, McCarthy DJ, Marioni JC (2016) A step-by-step workflow for low-level analysis of single-cell RNA-seq data with Bioconductor. *F1000Res* 5: 2122

Lundberg IE, Tjarnlund A, Bottai M, Werth VP, Pilkington C, Visser M, Alfredsson L, Amato AA, Barohn RJ, Liang MH *et al* (2017) 2017 European League Against Rheumatism/American College of Rheumatology classification criteria for adult and juvenile idiopathic inflammatory myopathies and their major subgroups. *Ann Rheum Dis* 76: 1955 – 1964

© 2023 The Authors

Lundberg IE, de Visser M, Werth VP (2018) Classification of myositis. *Nat Rev Rheumatol* 14: 269–278
Mackay LK, Minnich M, Kragten NA, Liao Y, Nota B, Seillet C, Zaid A, Man K, Preston S, Freestone D *et al* (2016) Hobit and Blimp1 instruct a universal transcriptional program of tissue residency in lymphocytes. *Science* 352: 459–463
Malmstrom V, Venalis P, Albrecht I (2012) T cells in myositis. *Arthritis Res Ther* 14: 230
Medsger TA Jr, Dawson WN Jr, Masi AT (1970) The epidemiology of polymyositis. *Am J Med* 48: 715–723
Meresse B, Curran SA, Ciszewski C, Orbelyan G, Setty M, Bhagat G, Lee L, Tretiakova M, Semrad C, Kistner E *et al* (2006) Reprogramming of CTLs into natural killer-like cells in celiac disease. *J Exp Med* 203: 1343–1355
Merlonghi G, Antonini G, Garibaldi M (2022) Immune-mediated necrotizing myopathy (IMNM): a myopathological challenge. *Autoimmun Rev* 21: 102993
Miller FW, Chen W, O'Hanlon TP, Cooper RG, Vencovsky J, Rider LG, Danko K, Wedderburn LR, Lundberg IE, Pachman LM *et al* (2015) Genome-wide association study identifies HLA 8.1 ancestral haplotype alleles as major genetic risk factors for myositis phenotypes. *Genes Immun* 16: 470–480
Nguyen QH, Pervolarakis N, Nee K, Kessenbrock K (2018) Experimental considerations for single-cell RNA sequencing approaches. *Front Cell Dev Biol* 6: 108
Pandya JM, Fasth AE, Zong M, Arnardottir S, Dani L, Lindroos E, Malmstrom V, Lundberg IE (2010) Expanded T cell receptor Vbeta-restricted T cells from patients with sporadic inclusion body myositis are proinflammatory and cytotoxic CD28null T cells. *Arthritis Rheum* 62: 3457–3466
Parekh S, Ziegenhain C, Vieth B, Enard W, Hellmann I (2018) zUMIs – a fast and flexible pipeline to process RNA sequencing data with UMIs. *Gigascience* 7: giy059
Parga-Vidal L, Behr FM, Kragten NAM, Nota B, Wesselink TH, Kavazovic I, Covill LE, Schuller MBP, Bryceson YT, Wensveen FM *et al* (2021) Hobit identifies tissue-resident memory T cell precursors that are regulated by Eomes. *Sci Immunol* 6: eabg3533
Peng YM, van de Garde MD, Cheng KF, Baars PA, Remmerswaal EB, van Lier RA, Mackay CR, Lin HH, Hamann J (2011) Specific expression of GPR56 by human cytotoxic lymphocytes. *J Leukoc Biol* 90: 735–740
Picelli S, Bjorklund AK, Faridani OR, Sagasser S, Winberg G, Sandberg R (2013) Smart-seq2 for sensitive full-length transcriptome profiling in single cells. *Nat Methods* 10: 1096–1098
Rao DA, Gurish MF, Marshall JL, Slowikowski K, Fonseka CY, Liu Y, Donlin LT, Henderson LA, Wei K, Mizoguchi F *et al* (2017) Pathologically expanded peripheral T helper cell subset drives B cells in rheumatoid arthritis. *Nature* 542: 110–114
Rothwell S, Cooper RG, Lundberg IE, Miller FW, Gregersen PK, Bowes J, Vencovsky J, Danko K, Limaye V, Selva-O'Callaghan A *et al* (2016) Dense genotyping of immune-related loci in idiopathic inflammatory myopathies confirms HLA alleles as the strongest genetic risk factor and suggests different genetic background for major clinical subgroups. *Ann Rheum Dis* 75: 1558–1566
Rothwell S, Chinoy H, Lamb JA, Miller FW, Rider LG, Wedderburn LR, McHugh NJ, Mammen AL, Betteridge ZE, Tansley SL *et al* (2019) Focused HLA analysis in Caucasians with myositis identifies significant associations with autoantibody subgroups. *Ann Rheum Dis* 78: 996–1002
Rubenstein AB, Smith GR, Raue U, Begue G, Minchev K, Ruf-Zamojski F, Nair VD, Wang X, Zhou L, Zaslavsky E *et al* (2020) Single-cell transcriptional profiles in human skeletal muscle. *Sci Rep* 10: 229
Satija R, Farrell JA, Gennert D, Schier AF, Regev A (2015) Spatial reconstruction of single-cell gene expression data. *Nat Biotechnol* 33: 495–502
Skon CN, Lee JY, Anderson KG, Masopust D, Hogquist KA, Jameson SC (2013) Transcriptional downregulation of S1pr1 is required for the establishment of resident memory $CD8^+$ T cells. *Nat Immunol* 14: 1285–1293
Stubbington MJT, Lonnberg T, Proserpio V, Clare S, Speak AO, Dougan G, Teichmann SA (2016) T cell fate and clonality inference from single-cell transcriptomes. *Nat Methods* 13: 329–332
Sugiura T, Murakawa Y, Nagai A, Kondo M, Kobayashi S (1999) Fas and Fas ligand interaction induces apoptosis in inflammatory myopathies: $CD4^+$ T cells cause muscle cell injury directly in polymyositis. *Arthritis Rheum* 42: 291–298
Svensson J, Arkema EV, Lundberg IE, Holmqvist M (2017) Incidence and prevalence of idiopathic inflammatory myopathies in Sweden: a nationwide population-based study. *Rheumatology (Oxford)* 56: 802–810
Szabo PA, Levitin HM, Miron M, Snyder ME, Senda T, Yuan J, Cheng YL, Bush EC, Dogra P, Thapa P *et al* (2019) Single-cell transcriptomics of human T cells reveals tissue and activation signatures in health and disease. *Nat Commun* 10: 4706
Tang Q, Gheorghe KR, Zhang XM, Lindroos E, Alexanderson H, Wick C, Bruton M, Fernandes-Cerqueira C, Harris RA, Nennesmo I *et al* (2020) Features of repeated muscle biopsies and phenotypes of monocytes in paired blood samples and clinical long-term response to treatment in patients with idiopathic inflammatory myopathy: a pilot study. *Clin Exp Rheumatol* 38: 42–49
Treiner E, Duban L, Bahram S, Radosavljevic M, Wanner V, Tilloy F, Affaticati P, Gilfillan S, Lantz O (2003) Selection of evolutionarily conserved mucosal-associated invariant T cells by MR1. *Nature* 422: 164–169
Troyanov Y, Targoff IN, Tremblay JL, Goulet JR, Raymond Y, Senecal JL (2005) Novel classification of idiopathic inflammatory myopathies based on overlap syndrome features and autoantibodies: analysis of 100 French Canadian patients. *Medicine (Baltimore)* 84: 231–249
Uxa S, Castillo-Binder P, Kohler R, Stangner K, Muller GA, Engeland K (2021) Ki-67 gene expression. *Cell Death Differ* 28: 3357–3370
Villalta SA, Rosenthal W, Martinez L, Kaur A, Sparwasser T, Tidball JG, Margeta M, Spencer MJ, Bluestone JA (2014) Regulatory T cells suppress muscle inflammation and injury in muscular dystrophy. *Sci Transl Med* 6: 258ra142
Waschbisch A, Schwab N, Ruck T, Stenner MP, Wiendl H (2010) $FOXP3^+$ T regulatory cells in idiopathic inflammatory myopathies. *J Neuroimmunol* 225: 137–142
Wein AN, McMaster SR, Takamura S, Dunbar PR, Cartwright EK, Hayward SL, McManus DT, Shimaoka T, Ueha S, Tsukui T *et al* (2019) CXCR6 regulates localization of tissue-resident memory CD8 T cells to the airways. *J Exp Med* 216: 2748–2762
Whiteside SK, Snook JP, Williams MA, Weis JJ (2018) Bystander T cells: a balancing act of friends and foes. *Trends Immunol* 39: 1021–1035
Zemmour D, Zilionis R, Kiner E, Klein AM, Mathis D, Benoist C (2018) Single-cell gene expression reveals a landscape of regulatory T cell phenotypes shaped by the TCR. *Nat Immunol* 19: 291–301

License: This is an open access article under the terms of the Creative Commons Attribution License, which permits use, distribution and reproduction in any medium, provided the original work is properly cited.

Expanded View Figures

Figure EV1. Smart-seq2 single-cell sequencing data from muscle T cells in patients with idiopathic inflammatory myopathies.

A Left panel: Violin plots showing the total number of genes captured, the number of transcripts and percentage of mitochondrial genes per patient. Right panel: UMAPs displaying before data integration and after data integration with Harmony package per sequencing batch (n = 6 IIM, n = 1 undefined myopathy).

B Violin plots showing the expression levels of selected differentially expressed genes per cluster in muscle T cells. Each color represents one T-cell cluster in the muscle tissue as indicated (n = 6 IIM, n = 1 undefined myopathy).

C Violin plot showing the expression level of *ITGA1* per patient in the four T_{RM} clusters. Each color represents one T_{RM} cluster as indicated (n = 6 IIM, n = 1 undefined myopathy).

D Violin plot showing the expression level of *KLRG1* in muscle T cells per patient.

Data information: CM: central memory; EM: effector memory, T_{RM}: tissue resident memory. IMNM: Immune-Mediated Necrotizing Myopathy; DM: DermatoMyositis; ASyS: AntiSYnthetase Syndrome; IBM: Inclusion Body Myositis; IIM: Idiopathic Inflammatory Myopathies (n = 6 IIM, n = 1 undefined myopathy).

© 2023 The Authors

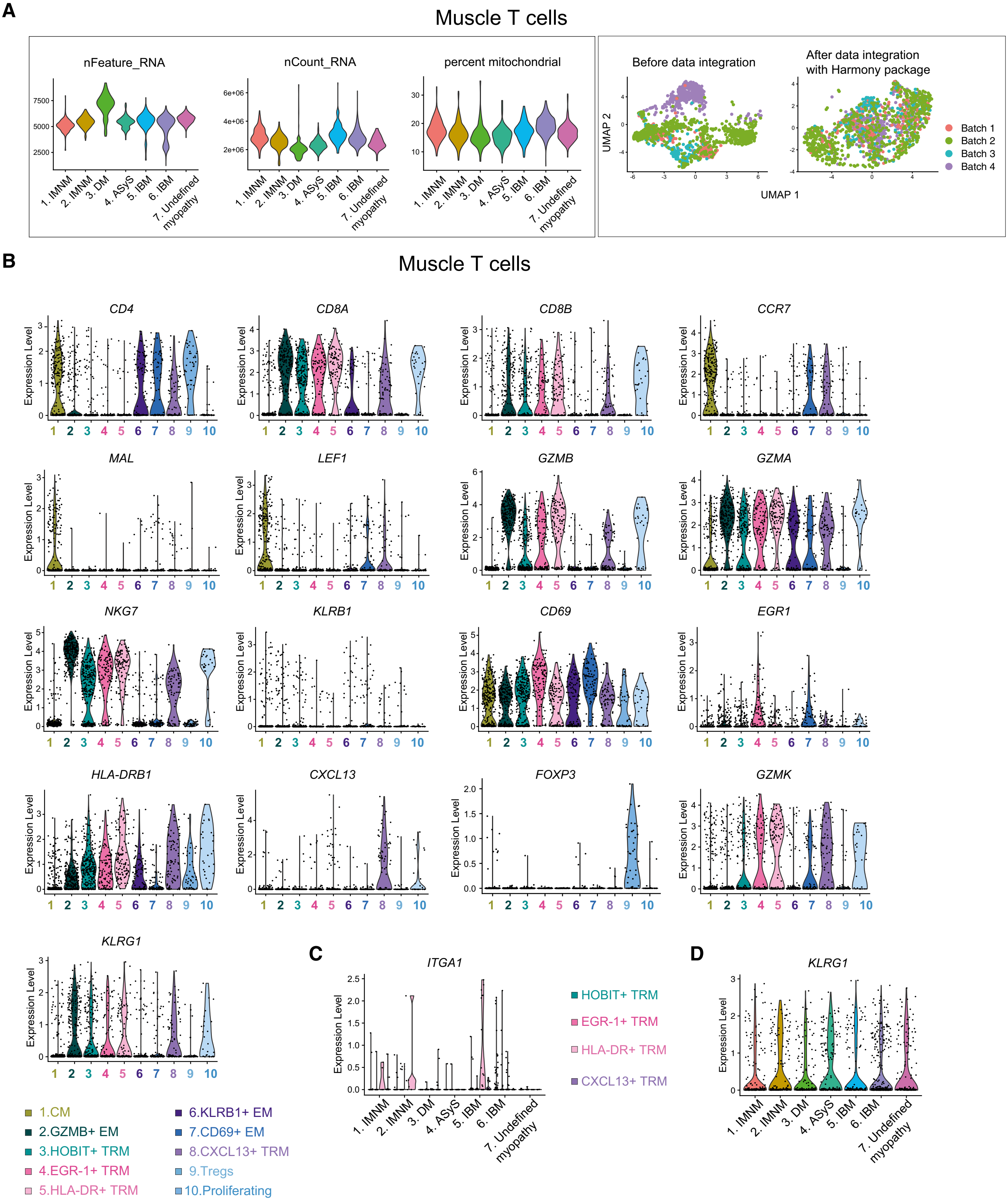
A
Muscle T cells
nFeature_RNA
nCount_RNA
percent mitochondrial
1. IMNM
2. IMNM
3. DM
4. ASyS
5. IBM
6. IBM
7. Undefined myopathy
Before data integration
After data integration with Harmony package
UMAP 2
UMAP 1
Batch 1
Batch 2
Batch 3
Batch 4
B
Muscle T cells
CD4
CD8A
CD8B
CCR7
MAL
LEF1
GZMB
GZMA
NKG7
KLRB1
CD69
EGR1
HLA-DRB1
CXCL13
FOXP3
GZMK
KLRG1
Expression Level
C
ITGA1
HOBIT+ TRM
EGR-1+ TRM
HLA-DR+ TRM
CXCL13+ TRM
D
KLRG1
1.CM
2.GZMB+ EM
3.HOBIT+ TRM
4.EGR-1+ TRM
5.HLA-DR+ TRM
6.KLRB1+ EM
7.CD69+ EM
8.CXCL13+ TRM
9.Tregs
10.Proliferating

Figure EV1.

Figure EV2. Smart-seq2 single-cell sequencing data from peripheral blood memory T cells in patients with idiopathic inflammatory myopathies.

A Left panel: Violin plots showing the total number of genes captured, the number of transcripts and percentage of mitochondrial genes per patient. Right panel: UMAPs displaying before data integration and after data integration with Harmony package per sequencing batch (n = 6 IIM, n = 1 undefined myopathy).
B Violin plots showing the expression level of selected genes in peripheral blood memory T cells from patients with IIM (n = 6 IIM, n = 1 undefined myopathy).
C UMAP feature plots displaying the normalized expression of *KLRC1*, *KLRC2*, *FCGR3A* and *IKZF2* genes.
D UMAP feature plot showing cells using *TRAV1-2* and *TRAV10* genes.
E Flow cytometry dot plots showing the frequency of $CD3^{+}CD8^{+}$ T cells in peripheral blood (PB) of patients with DM (left) and IMNM (right) patients.
F Frequency of $CD3^{+}CD8^{+}$ T cells in PB of patients with IIM.

Data information: CM: central memory; EM: effector memory. IMNM: Immune-Mediated Necrotizing Myopathy; DM: DermatoMyositis; ASyS: AntiSYnthetase Syndrome; IBM: Inclusion Body Myositis; IIM: Idiopathic Inflammatory Myopathies.

 © 2023 The Authors

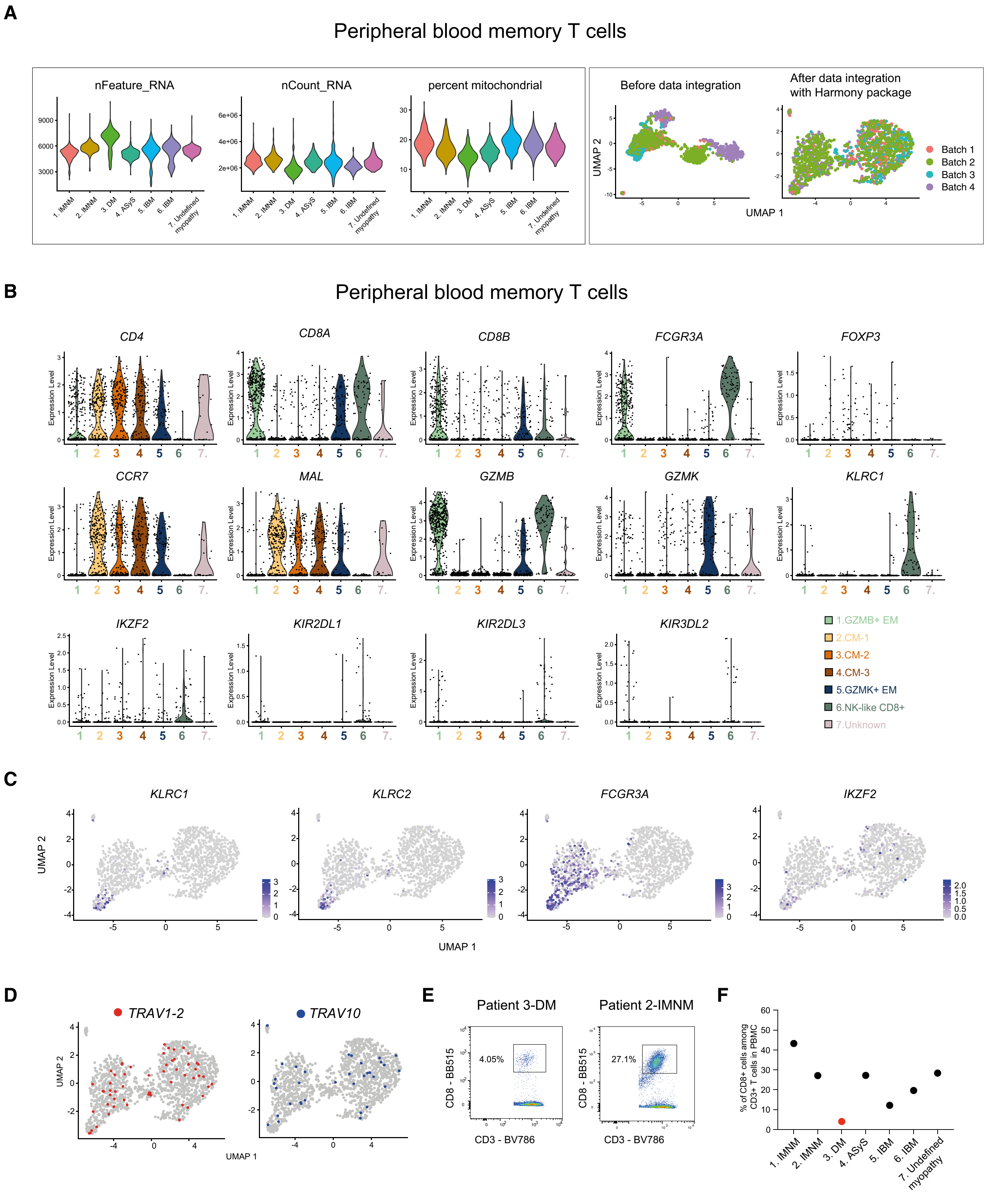
A
Peripheral blood memory T cells
nFeature_RNA
nCount_RNA
percent mitochondrial
1. IMNM
2. IMNM
3. DM
4. ASyS
5. IBM
6. IBM
7. Undefined myopathy
Before data integration
After data integration with Harmony package
UMAP 2
UMAP 1
Batch 1
Batch 2
Batch 3
Batch 4
B
Peripheral blood memory T cells
CD4
CD8A
CD8B
FCGR3A
FOXP3
CCR7
MAL
GZMB
GZMK
KLRC1
IKZF2
KIR2DL1
KIR2DL3
KIR3DL2
Expression Level
1.GZMB+ EM
2.CM-1
3.CM-2
4.CM-3
5.GZMK+ EM
6.NK-like CD8+
7.Unknown
C
KLRC1
KLRC2
FCGR3A
IKZF2
UMAP 2
UMAP 1
D
TRAV1-2
TRAV10
UMAP 2
UMAP 1
E
Patient 3-DM
Patient 2-IMNM
4.05%
27.1%
CD8 - BB515
CD3 - BV786
F
% of CD8+ cells among CD3+ T cells in PBMC
1. IMNM
2. IMNM
3. DM
4. ASyS
5. IBM
6. IBM
7. Undefined myopathy

Figure EV2.

Figure EV3. Single-cell sequencing data from healthy donors and patients with idiopathic inflammatory myopathies.

A Workflow for Smart-seq3 sequencing.
B UMAP displaying eight memory T-cell clusters identified in peripheral blood (PB) of healthy donors (n = 439 cells, n = 3 donors).
C UMAP feature plots displaying the normalized expression of *CD4*, *CD8A*, *CD8B*, *IKZF2*, *KLRC1* and *KLRC2*.
D Heatmap showing the normalized and scaled expression of the top 10 differentially expressed genes per cluster (Wilcoxon Rank Sum, P value < 0.009; $\log_2$ fold change > 0.25).
E Violin plots showing the expression level of *TRAV1-2* and *TRAV10* genes per cluster in PB memory T cells from healthy donors (n = 3).
F Heatmap showing the normalized and scaled expression of the top 25 upregulated and top 25 downregulated genes sorted by average log2FC values (Wilcoxon Rank Sum, P value $< 10^{-15}$, $-2.6 < \log_2$ fold change < 4.2).
G Heatmap showing the normalized and scaled expression of the top 25 upregulated and top 25 downregulated genes in muscle T cells versus PB memory T cells from patients with IIM after filtering out genes identified in Dataset EV5 (Wilcoxon Rank Sum, P value $< 8 \times 10^{-5}$, $-0.25 < \log_2$ fold change < 2.3).
H Dotplots showing selected genes in PB of patients with IIM (n = 7).
I Dotplots showing selected genes in PB of healthy donors (n = 3).

Data information: CM: central memory; EM: effector memory. IMNM: Immune-Mediated Necrotizing Myopathy; DM: DermatoMyositis; ASyS: AntiSYnthetase Syndrome; IBM: Inclusion Body Myositis; IIM: Idiopathic Inflammatory Myopathies; HD: healthy donors.

© 2023 The Authors

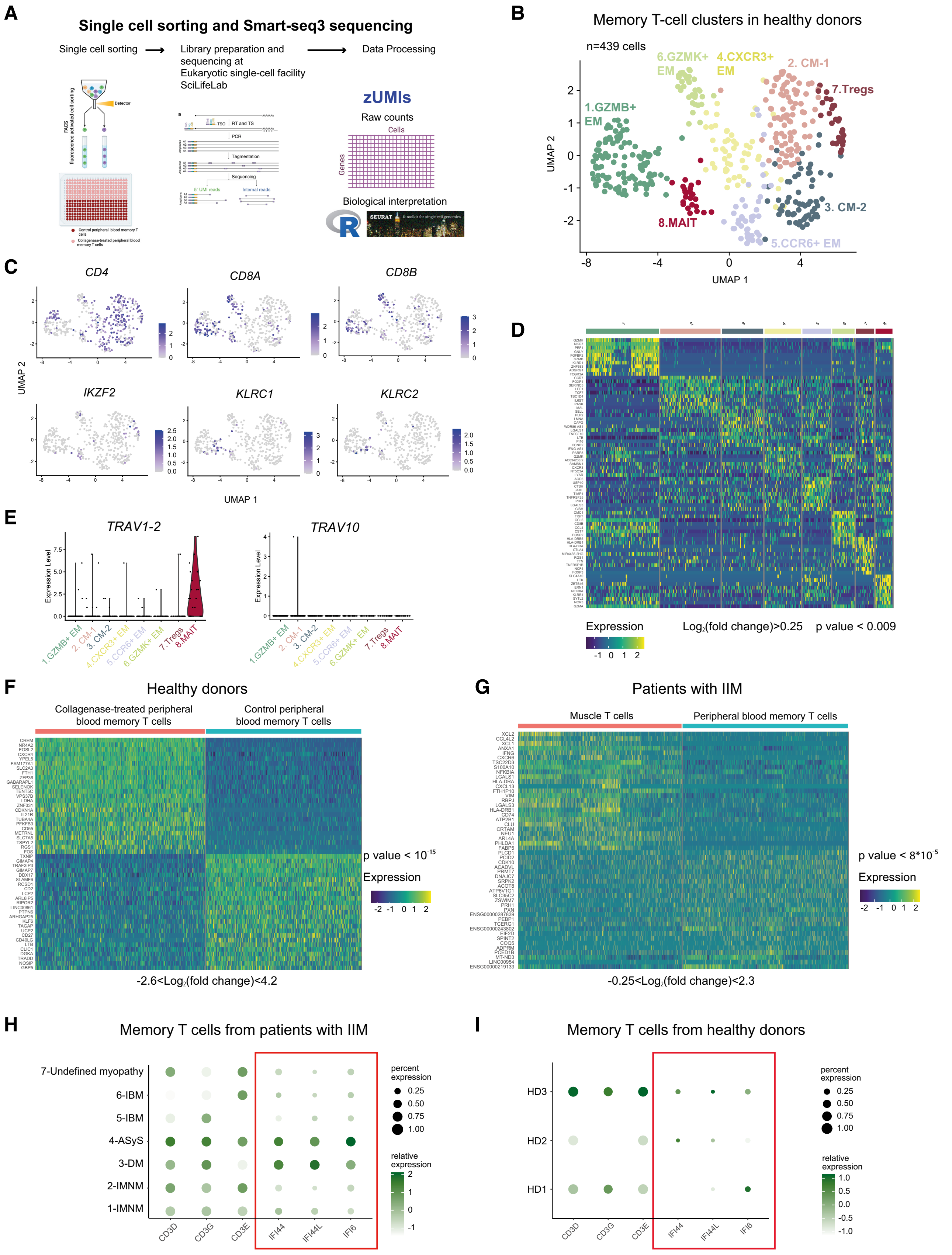
A
Single cell sorting and Smart-seq3 sequencing
Single cell sorting
Library preparation and sequencing at Eukaryotic single-cell facility SciLifeLab
Data Processing
zUMIs
Raw counts
Cells
Genes
Biological interpretation
Control peripheral blood memory T cells
Collagenase-treated peripheral blood memory T cells
B
Memory T-cell clusters in healthy donors
n=439 cells
1.GZMB+ EM
2. CM-1
3. CM-2
4.CXCR3+ EM
5.CCR6+ EM
6.GZMK+ EM
7.Tregs
8.MAIT
UMAP 1
UMAP 2
C
CD4
CD8A
CD8B
IKZF2
KLRC1
KLRC2
UMAP 1
UMAP 2
D
Expression
Log2(fold change)>0.25
p value < 0.009
E
TRAV1-2
TRAV10
Expression Level
F
Healthy donors
Collagenase-treated peripheral blood memory T cells
Control peripheral blood memory T cells
p value < 10-15
Expression
-2.6<Log2(fold change)<4.2
G
Patients with IIM
Muscle T cells
Peripheral blood memory T cells
p value < 8*10-5
Expression
-0.25<Log2(fold change)<2.3
H
Memory T cells from patients with IIM
7-Undefined myopathy
6-IBM
5-IBM
4-ASyS
3-DM
2-IMNM
1-IMNM
CD3D
CD3G
CD3E
IFI44
IFI44L
IFI6
percent expression
relative expression
I
Memory T cells from healthy donors
HD3
HD2
HD1
CD3D
CD3G
CD3E
IFI44
IFI44L
IFI6
percent expression
relative expression

Figure EV3.

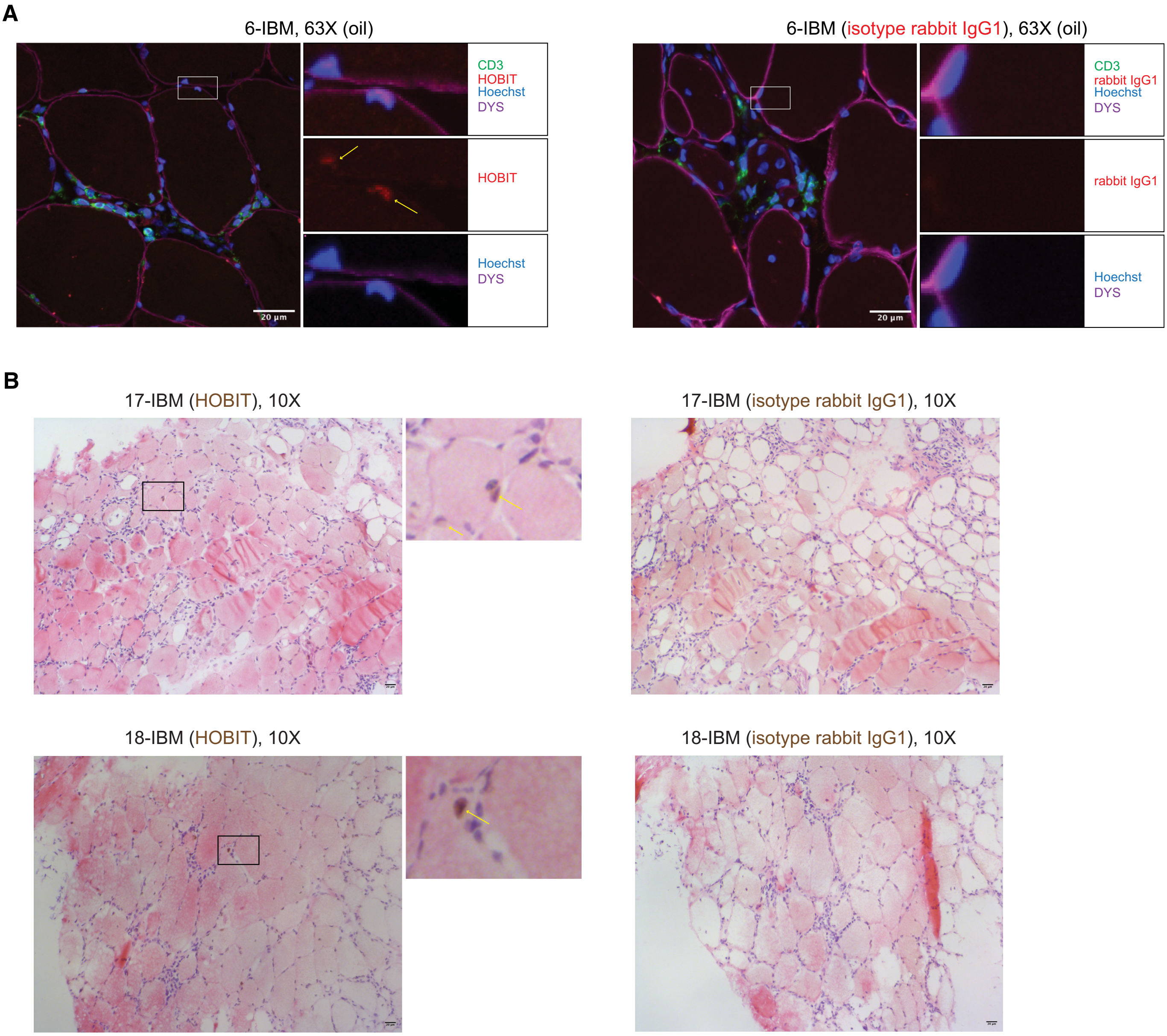

Figure EV4. Immunofluorescence and immunohistochemistry stainings showing HOBIT expression in muscle fibers of biopsies from patients with inclusion body myositis.

A Representative immunofluorescence stainings of HOBIT (red), CD3 (green), dystrophin (purple) and Hoechst 33342 (blue) performed on muscle tissue from patient 6 (IBM) (left panel) and isotype rabbit IgG1 staining (red) (right panel). Images were acquired using a LSM 880 confocal without Airyscan microscope (63× oil objective). Scale bar = 20 µm. The yellow arrows show HOBIT expression in muscle nuclei.

B Representative immunohistochemistry stainings of HOBIT (brown, left panels), and isotype rabbit IgG (right panels) with hematoxylin (purple) and eosin (red) stainings performed on muscle tissue of two patients with IBM. Images were acquired using a Leica Reichert Polyvar 2 light microscope (10× objective). Scale bar = 20 µm. The yellow arrows show HOBIT expression in muscle nuclei. IBM: Inclusion Body Myositis.

© 2023 The Authors

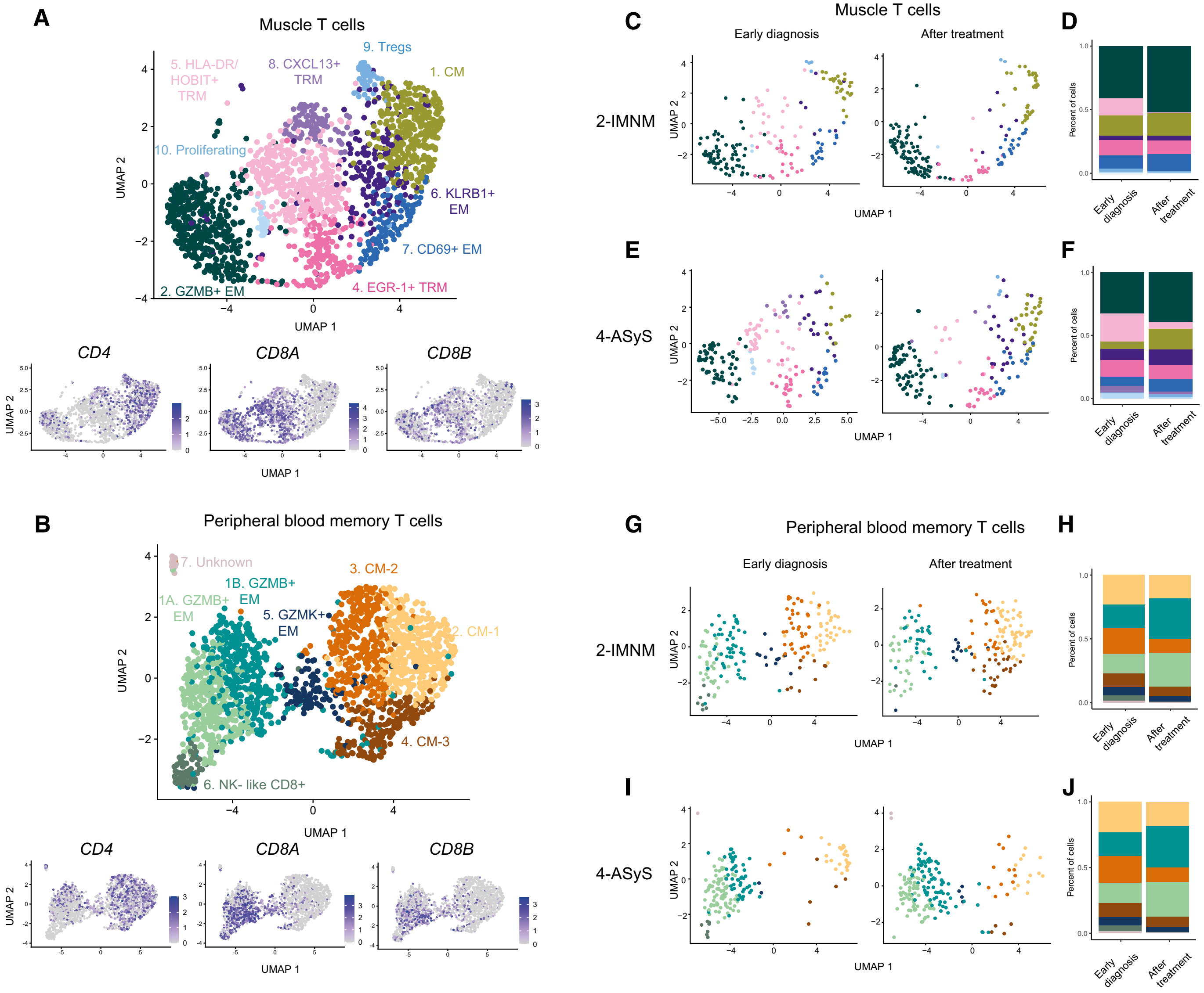

Figure EV5. Smart-seq2 single-cell data from muscle T cell and peripheral blood memory T cells from patients at early diagnosis and after treatment.

A Upper Panel: UMAP displaying nine T-cell clusters in the muscle of patients with idiopathic inflammatory myopathies (IIM) at early diagnosis ($n = 7$) and after treatment ($n = 2$) ($n = 1747$ cells). Lower panel: UMAP feature plots displaying the normalized expression of *CD4*, *CD8A*, and *CD8B*.

B Upper panel: UMAP displaying eight T-cell clusters in peripheral blood (PB) memory T cells of patients with IIM at early diagnosis ($n = 7$) and after treatment ($n = 2$) ($n = 1788$ cells). Lower panel: UMAP feature plots displaying the normalized expression of *CD4*, *CD8A* and *CD8B*.

C UMAP displaying nine T-cell clusters split by treatment status in muscle of patient 2 (IMNM).

D Stacked bar plots of T-cell cluster composition in early diagnosis and after treatment in muscle of patient 2 (IMNM).

E UMAP displaying nine T-cell clusters split by treatment status in muscle of patient 4 (ASyS).

F Stacked bar plots of T-cell cluster composition in early diagnosis and after treatment in muscle of patient 4 (ASyS).

G UMAP displaying eight T-cell clusters split by treatment status in PB of patient 2 (IMNM).

H Stacked bar plots of T-cell cluster composition in early diagnosis and after treatment in PB of patient 2 (IMNM).

I UMAP displaying eight T-cell clusters split by treatment status in PB of patient 4 (ASyS).

J Stacked bar plots of T-cell cluster composition in early diagnosis and after treatment in PB of patient 4 (ASyS).

Data information: IMNM: Immune-Mediated Necrotizing Myopathy; ASyS: AntiSYnthetase Syndrome; IIM: Idiopathic Inflammatory Myopathies.

Single-cell profiling of muscle-infiltrating T cells in idiopathic inflammatory myopathies

Alexandra Argyriou[1,2,6], Begum Horuluoglu[1,2,6], Angeles Shunashy Galindo-Feria[1,2,6], Juan Sebastian Diaz-Boada[1,2], Merel Sijbranda[1,2], Antonella Notarnicola[1,2,3], Lara Dani[1,3], Annika van Vollenhoven[1,2], Daniel Ramsköld[4], Inger Nennesmo[5], Maryam Dastmalchi[1,2,3], Ingrid E. Lundberg[1,2,3], Lina-Marcela Diaz-Gallo[1,2,7] and Karine Chemin[1,2,7]

[1]Division of Rheumatology, Department of Medicine, Solna, Karolinska Institutet, Stockholm, Sweden

[2]Center for Molecular Medicine, Karolinska Institutet, Stockholm, Sweden

[3]Department of Gastro, Dermatology and Rheumatology, Karolinska University Hospital, Stockholm, Sweden

[4]Department of Cell and Molecular Biology, Karolinska Institutet, Stockholm, Sweden

[5]Department of Pathology, Karolinska University Hospital, Stockholm, Sweden.

[6]co-first authors contributed equally to this manuscript, and each has the right to list themselves first in author order on their CVs

[7]equal senior contribution

Correspondence to:

Karine Chemin (karine.chemin@ki.se)

The authors declare that they have no conflict of interest.

TABLE OF CONTENT

Appendix Figure S1: Immune infiltrates in muscle biopsies from 6 patients with Idiopathic inflammatory myopathies and one patient with undefined myopathy

Appendix Figure S2: Flow cytometry gating strategy

Appendix Figure S3: Workflow representing the Smart-seq2 single-cell sequencing methodology

Appendix Table S1: Reagents

Appendix Table S2: Characteristics of additional patients with IIM from whom muscle biopsies were used for immunofluorescence and immunohistochemistry stainings

Appendix Figure S1

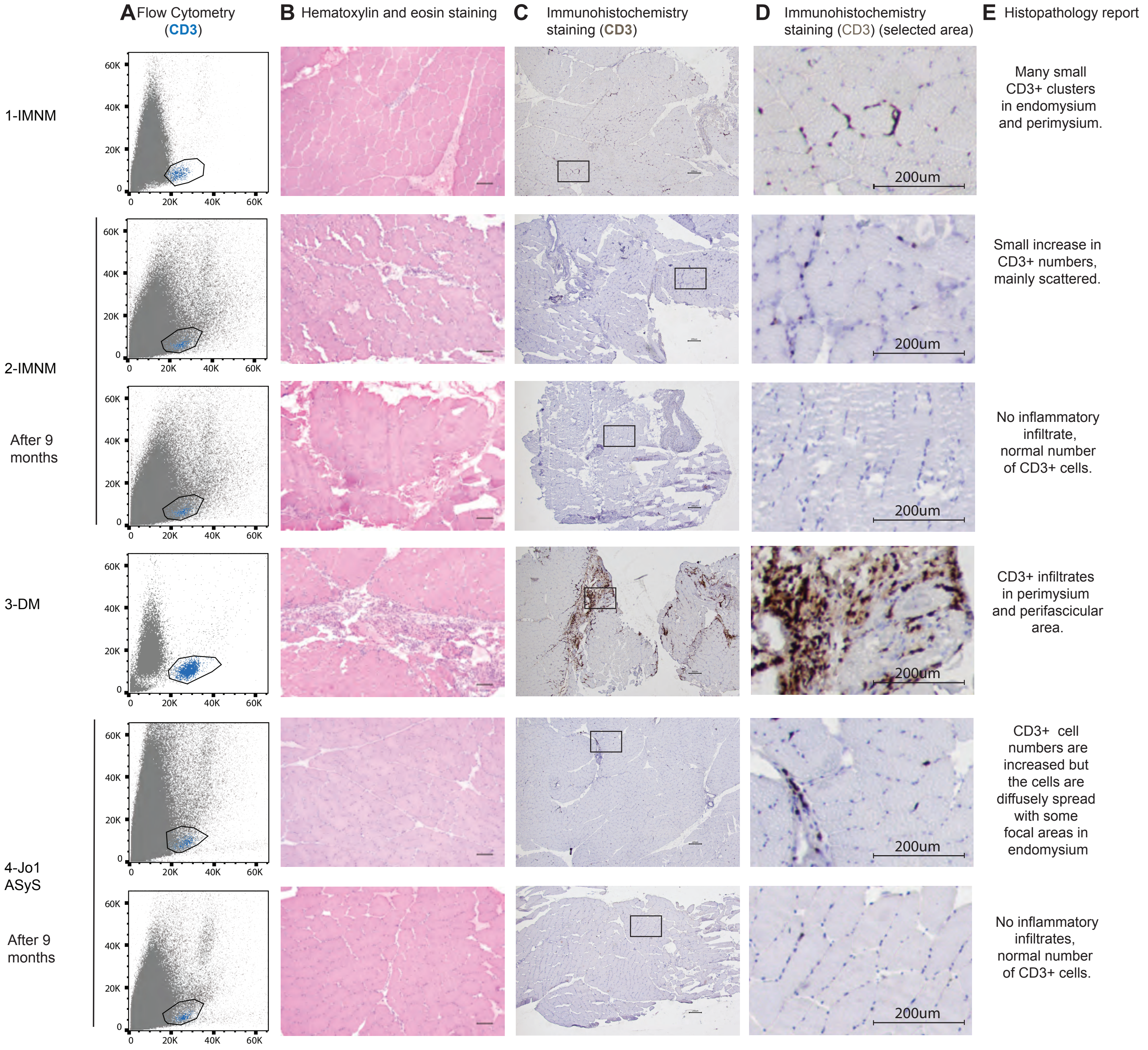

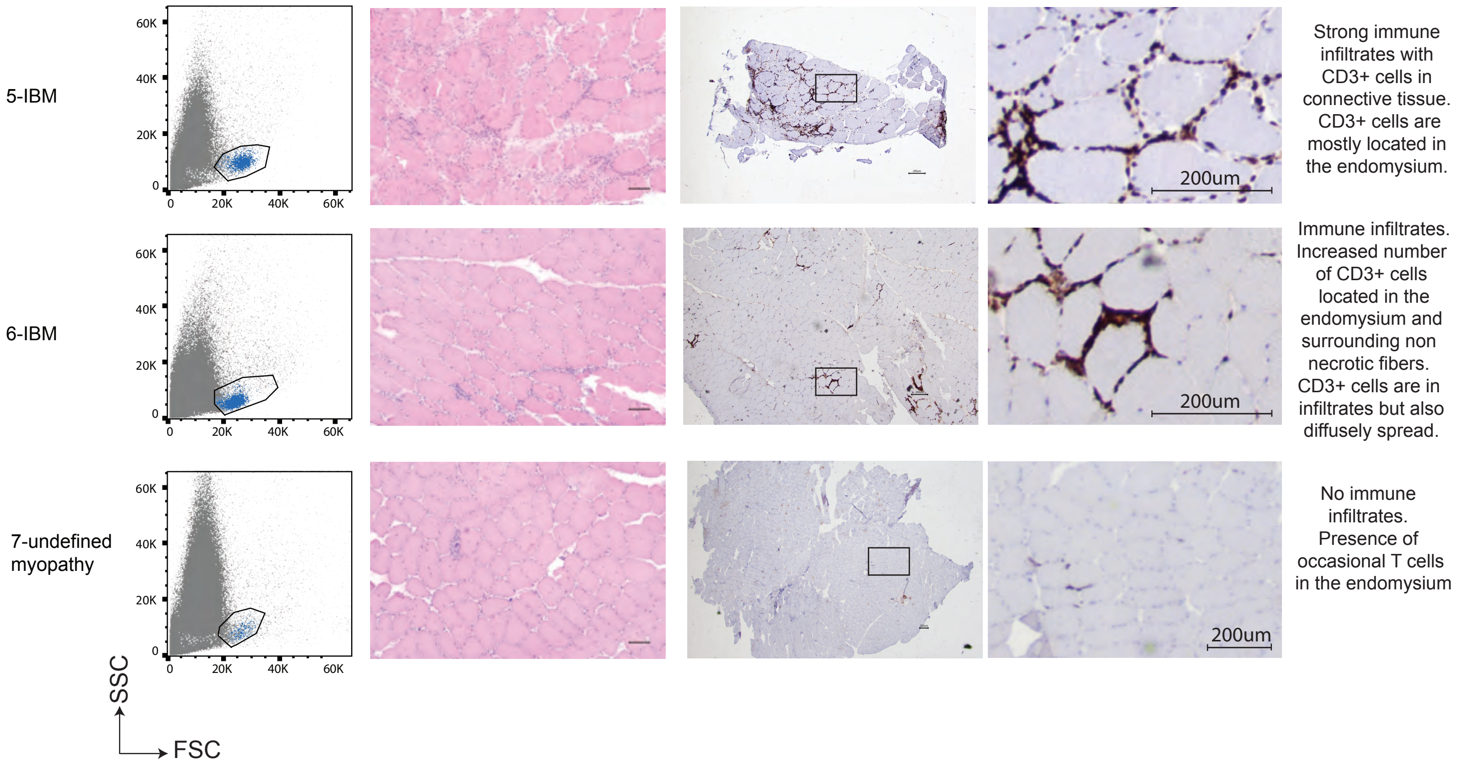

Appendix Figure S1. Immune infiltrates in muscle biopsies from six patients with myositis (1-6) and one patient with undefined myopathy (7). A) Flow cytometry plot showing CD45+CD3+ lymphocyte infiltrates (in blue) in muscle biopsies. **B)** Hematoxylin and eosin staining of muscle biopsies, scale bar=100um. **C)** Immunohistochemistry staining showing CD3+ lymphocytes (in brown), scale bar=200um **D)** selected square area from **C)** show examples of T cell infiltrates. **E)** Histopathology report. SSC: side scatter, FSC: forward side scatter. IMNM:Immune-Mediated Necrotizing Myopathy; DM: DermatoMyositis; ASyS:AntiSYnthetase Syndrome; IBM:Inclusion Body Myositis.

Appendix Figure S2

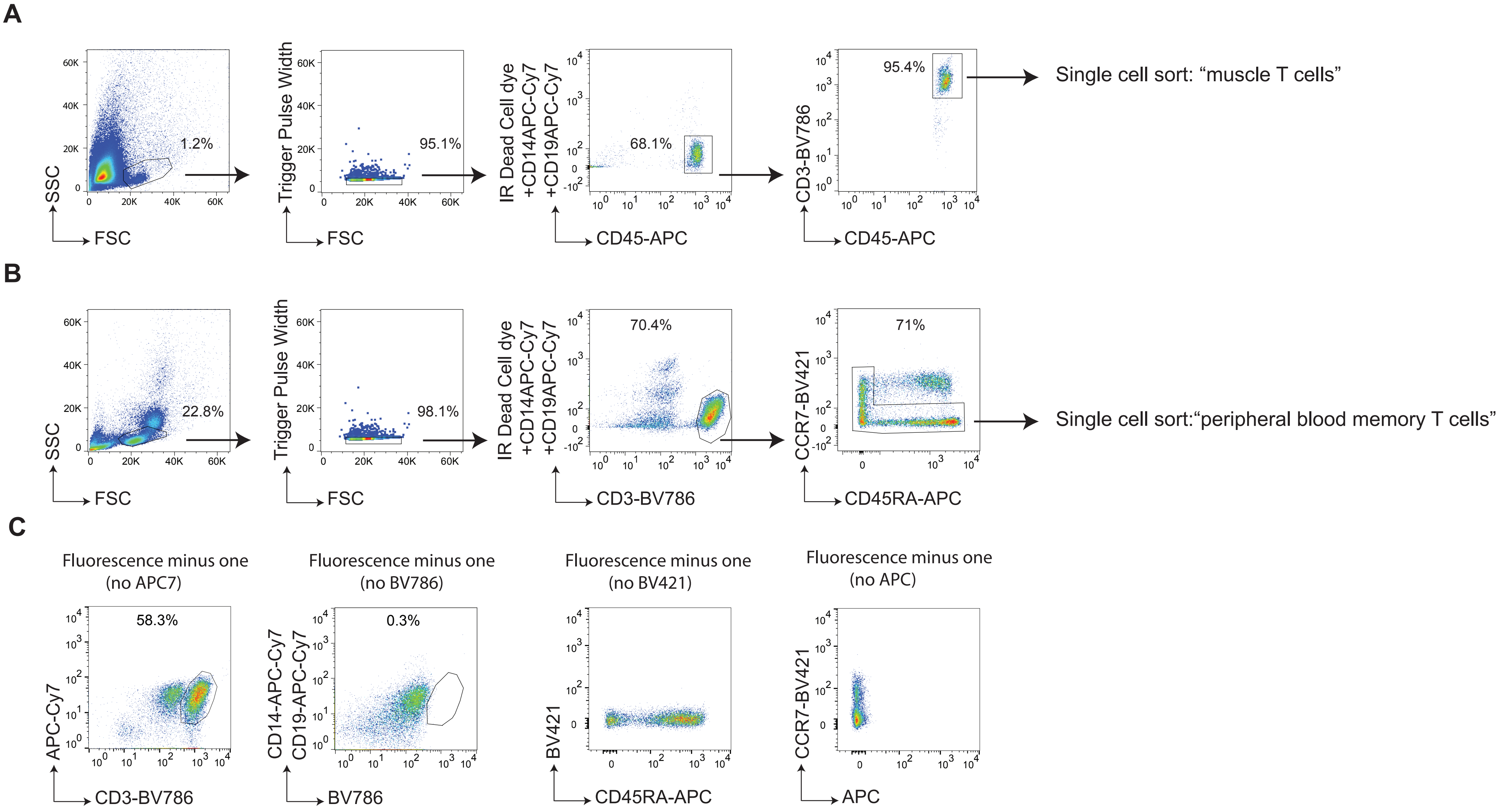

Appendix Figure S2. A) Flow cytometry gating strategy for single cell sorting of T cells from muscle biopsy included lymphocyte gating (based on forward and side scatter) followed by doublets, CD14+, CD19+ and dead cells exclusion.CD45+ CD3+ T cells were then further selected. Representative patient 6-IBM depicted. **B**) Flow cytometry gating strategy for single cell sorting of memory T cells from peripheral blood included lymphocyte gating (based on forward and side scatter) followed by doublets, CD14+,CD19+ and dead cells exclusion. CD3+ and memory T cells were then further selected (by excluding CCR7+ CD45RA+ cells). Representative patient 6-IBM depicted. **C**) Fluoroscence minus one controls performed on peripheral blood from an healthy donor.

Appendix Figure S3

Single cell sorting, Smart-seq2 RNA single cell sequencing and analysis overview

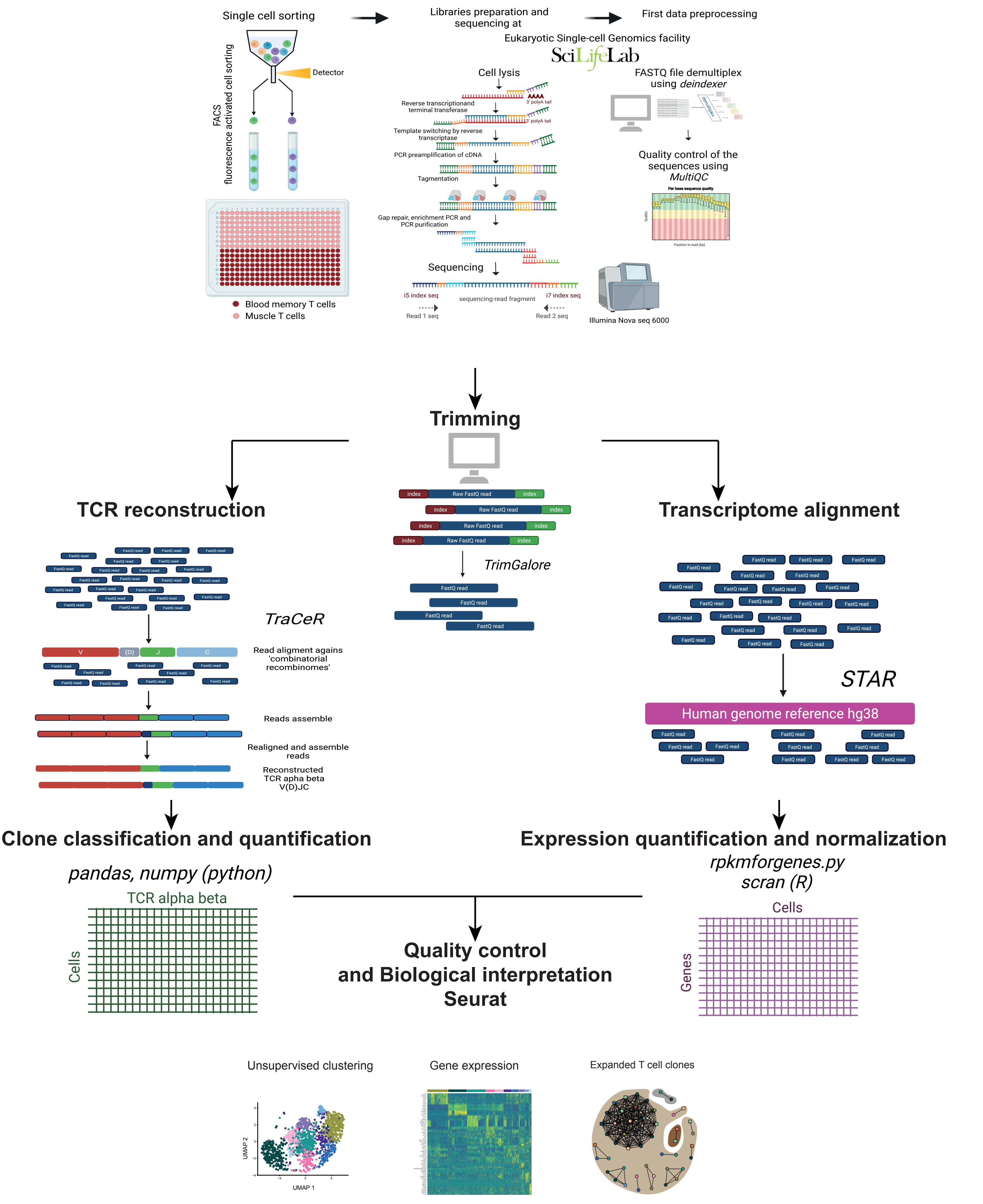

Appendix Figure 3. Workflow showing the methodology used for the RNA single-cell sequencing. The procedure starts with single-cell sorting, library preparation, and smart-seq2 single cell RNA sequencing, followed by data processing and analysis representation. The analysis contains two branches, one for the T cell receptor (TCR) reconstruction and the other for the transcriptome analyses. These branches converged in the biological interpretation using python and Seurat in R.

Appendix Table S1. Reagents

Flow cytometry single cell sorting antibodies				
**Antibody**	**Fluorochrome**	**Clone**	**Source**	**Reference**
CD3	BV786	SK7	BD Bioscience	563800
CD4	APC	RPAT4	BD Bioscience	561841
CD4	BV421	RPAT4	BD Bioscience	562424
CD8	BB515	RPAT8	BD Bioscience	564526
CCR7	BV421	G043H7	Biolegend	353208
CD45RA	PECY7	L48	BD Bioscience	337186
CD45	APC	HI30	BD Bioscience	561864
CD14	APC-H7	MΦP9	BD Bioscience	560180
CD19	APC-H7	SJ25C1	BD Bioscience	560177
gd	PE	REA 591	Miltenyi Biotec	130-114-038

Confocal antibodies				
**Antibody**	**Fluorochrome**	**Clone**	**Source**	**Reference**
mouse anti-human CD3	unconjugated	SK7	BD Bioscience	347340
rabbit anti-human HOBIT	unconjugated	polyclonal	ThermoFisher	PA5-54902
rabbit anti-human Dystrophin	Alexa Fluor 647	monoclonal	abcam	ab282171
mouse IgG1	unconjugated	monoclonal	DAKO	X0931
rabbit IgG	unconjugated	polyclonal	Invitrogen	02-6102
goat anti-mouse	Alexa Fluor 488	polyclonal	ThermoFisher	A-11029
goat anti-rabbit	Alexa Fluor 647	polyclonal	Cell Signaling	4414
donkey anti-rabbit	Cy3	polyclonal	Jackson Immunoresearch	711-165-152

Dyes

Name	Source	Reference
Hoeschst 333342	ThermoFischer	3570
Near-IR Dead Cell Stain Kit	Invitrogen	L10119

Appendix Table S2. Characteristics of additional patients used for immunofluorescence and immunohistochemistry stainings

Patient number	Diagnosis	Age interval at onset	Disease duration at time of biopsy (months)	Manual Muscle Testing (MMT8) (a)	Extramuscular global assessment (VAS) (b)	PhyGA (VAS) (c)	Health Assessment Questionnaire (HAQ)(d)	Health-related quality of life EQ-5D-5L (e)	Creatinine Kinase (CK, microcat/l)	Extra-muscular manifestations	Myositis-specific autoantibodies (MSA)	Antinuclear Antibodies (ANA)	Treatment at time of biopsy	Histology report	Flow cytometry immune infiltrate (f)	Biopsy location
16	IBM	70-75	120	66	32		NA	0.552	5.6	Dysphagia	negative	Negative	Methotrexate	-MHCI in several fibers -CD3 and CD8 in necrotic fibers	NA	Anterior tibialis (right)
17	IBM	65-70	24	45	20		NA	NA	6.6		SSA/SSB+	ANA+	Methotrexate	NA	NA	Vastus lateralis (left)
18	IBM	60-65	24	68	NA		NA	NA	5.4		NA	NA	Azathioprine	-MHCI staining -CD3 cell infiltration	NA	Vastus lateralis (right)
