# Supplementary material for: Single‐cell profiling of muscle‐infiltrating T cells in idiopathic inflammatory myopathies
Source: EMBO Mol Med. 2023 Jul 31;15(10):e17240. doi: 10.15252/emmm.202217240 (PMC10565639; doi:10.15252/emmm.202217240)
Supplement: Supplementary file 22 [file EMMM-15-e17240-s016.pdf]

EMBO Molecular Medicine

Single-cell profiling of muscle-infiltrating T cells in idiopathic inflammatory myopathies

Alexandra Argyriou, Begum Horuluoglu, Angeles Galindo-Feria, Juan Diaz-Boada, Merel Sijbranda, Antonella Notarnicola, Lara Dani, Annika van Vollenhoven, Daniel Ramsköld, Inger Nennesmo, Maryam Dastmalchi, Ingrid Lundberg, Lina Marcela Diaz-Gallo, and Karine Chemin

DOI: 10.15252/emmm.202217240

Corresponding authors: Karine Chemin (karine.chemin@ki.se) , Lina Marcela Diaz-Gallo (lina.diaz@ki.se)

Review Timeline:

Submission Date:	29th Nov 22
Editorial Decision:	10th Jan 23
Revision Received:	12th May 23
Editorial Decision:	15th Jun 23
Revision Received:	4th Jul 23
Accepted:	10th Jul 23

Editor: Lise Roth

Transaction Report:

(Note: With the exception of the correction of typographical or spelling errors that could be a source of ambiguity, letters and reports are not edited. Depending on transfer agreements, referee reports obtained elsewhere may or may not be included in this compilation. Referee reports are anonymous unless the Referee chooses to sign their reports.)

10th Jan 2023

Dear Dr. Chemin,

Thank you for the submission of your manuscript to EMBO Molecular Medicine, and please accept my apologies for the delay in getting back to you during this busy time of the year. We have now received feedback from the three reviewers who agreed to evaluate your manuscript. As you will see from the reports below, the referees acknowledge the interest of the study and are overall supporting publication of your work pending appropriate revisions.

Addressing the reviewers' concerns in full will be necessary for further considering the manuscript in our journal, except for the addition of healthy controls (referee #3). Indeed, we realize this might be difficult (if not impossible) and would rather welcome an adequate discussion on this point.

EMBO Molecular Medicine encourages a single round of revision only and therefore, acceptance or rejection of the manuscript will depend on the completeness of your responses included in the next, final version of the manuscript. For this reason, and to save you from any frustrations in the end, I would strongly advise against returning an incomplete revision.

If you would like to discuss further the points raised by the referees, I am available to do so via email or video. Let me know if you are interested in this option.

Revised manuscripts should be submitted within three months of a request for revision; they will otherwise be treated as new submissions, except under exceptional circumstances in which a short extension is obtained from the editor.

When submitting your revised manuscript, please carefully review the instructions that follow below. We perform an initial quality control of all revised manuscripts before re-review; failure to include requested items will delay the evaluation of your revision.

We require:

1) A .docx formatted version of the manuscript text (including legends for main figures, EV figures and tables). Please make sure that the changes are highlighted to be clearly visible.

2) Individual production quality figure files as .eps, .tif, .jpg (one file per figure). For guidance, download the 'Figure Guide PDF' (https://www.embopress.org/page/journal/17574684/authorguide#figureformat).

3) At EMBO Press we ask authors to provide source data for the main figures. Our source data coordinator will contact you to discuss which figure panels we would need source data for and will also provide you with helpful tips on how to upload and organize the files.

4) A .docx formatted letter INCLUDING the reviewers' reports and your detailed point-by-point responses to their comments. As part of the EMBO Press transparent editorial process, the point-by-point response is part of the Review Process File (RPF), which will be published alongside your paper.

5) A complete author checklist, which you can download from our author guidelines (https://www.embopress.org/page/journal/17574684/authorguide#submissionofrevisions). Please insert information in the checklist that is also reflected in the manuscript. The completed author checklist will also be part of the RPF.

6) It is mandatory to include a 'Data Availability' section after the Materials and Methods. Before submitting your revision, primary datasets produced in this study need to be deposited in an appropriate public database, and the accession numbers and database listed under 'Data Availability'. Please remember to provide a reviewer password if the datasets are not yet public (see https://www.embopress.org/page/journal/17574684/authorguide#dataavailability).

In case you have no data that requires deposition in a public database, please state so in this section. Note that the Data Availability Section is restricted to new primary data that are part of this study.

7) For data quantification: please specify the name of the statistical test used to generate error bars and P values, the number (n) of independent experiments (specify technical or biological replicates) underlying each data point and the test used to calculate p-values in each figure legend. The figure legends should contain a basic description of n, P and the test applied. Graphs must include a description of the bars and the error bars (s.d., s.e.m.). Please provide exact p values.

8) Our journal encourages inclusion of *data citations in the reference list* to directly cite datasets that were re-used and obtained from public databases. Data citations in the article text are distinct from normal bibliographical citations and should

directly link to the database records from which the data can be accessed. In the main text, data citations are formatted as follows: "Data ref: Smith et al, 2001" or "Data ref: NCBI Sequence Read Archive PRJNA342805, 2017". In the Reference list, data citations must be labeled with "[DATASET]". A data reference must provide the database name, accession number/identifiers and a resolvable link to the landing page from which the data can be accessed at the end of the reference. Further instructions are available at .

9) We replaced Supplementary Information with Expanded View (EV) Figures and Tables that are collapsible/expandable online. A maximum of 5 EV Figures can be typeset. EV Figures should be cited as 'Figure EV1, Figure EV2" etc... in the text and their respective legends should be included in the main text after the legends of regular figures.
- For the figures that you do NOT wish to display as Expanded View figures, they should be bundled together with their legends in a single PDF file called *Appendix*, which should start with a short Table of Content. Appendix figures should be referred to in the main text as: "Appendix Figure S1, Appendix Figure S2" etc.
- Additional Tables/Datasets should be labeled and referred to as Table EV1, Dataset EV1, etc. Legends have to be provided in a separate tab in case of .xls files. Alternatively, the legend can be supplied as a separate text file (README) and zipped together with the Table/Dataset file.
See detailed instructions here:

10) The paper explained: EMBO Molecular Medicine articles are accompanied by a summary of the articles to emphasize the major findings in the paper and their medical implications for the non-specialist reader. Please provide a draft summary of your article highlighting
- the medical issue you are addressing,
- the results obtained and
- their clinical impact.
This may be edited to ensure that readers understand the significance and context of the research. Please refer to any of our published articles for an example.

11) For more information: There is space at the end of each article to list relevant web links for further consultation by our readers. Could you identify some relevant ones and provide such information as well? Some examples are patient associations, relevant databases, OMIM/proteins/genes links, author's websites, etc...

12) Author contributions: CRediT has replaced the traditional author contributions section because it offers a systematic machine readable author contributions format that allows for more effective research assessment. Please remove the Authors Contributions from the manuscript and use the free text boxes beneath each contributing author's name in our system to add specific details on the author's contribution. More information is available in our guide to authors.

13) Disclosure statement and competing interests: We updated our journal's competing interests policy in January 2022 and request authors to consider both actual and perceived competing interests. Please review the policy https://www.embopress.org/competing-interests and update your competing interests if necessary.

14) Every published paper now includes a 'Synopsis' to further enhance discoverability. Synopses are displayed on the journal webpage and are freely accessible to all readers. They include a short stand first (maximum of 300 characters, including space) as well as 2-5 one-sentences bullet points that summarizes the paper. Please write the bullet points to summarize the key NEW findings. They should be designed to be complementary to the abstract - i.e. not repeat the same text. We encourage inclusion of key acronyms and quantitative information (maximum of 30 words / bullet point). Please use the passive voice. Please attach these in a separate file or send them by email, we will incorporate them accordingly.

Please also suggest a striking image or visual abstract to illustrate your article as a PNG file 550 px wide x 300-600 px high.

15) As part of the EMBO Publications transparent editorial process initiative (see our Editorial at http://embomolmed.embopress.org/content/2/9/329), EMBO Molecular Medicine will publish online a Review Process File (RPF) to accompany accepted manuscripts.
In the event of acceptance, this file will be published in conjunction with your paper and will include the anonymous referee reports, your point-by-point response and all pertinent correspondence relating to the manuscript. Let us know whether you agree with the publication of the RPF and as here, if you want to remove or not any figures from it prior to publication.
Please note that the Authors checklist will be published at the end of the RPF.

EMBO Molecular Medicine has a "scooping protection" policy, whereby similar findings that are published by others during review or revision are not a criterion for rejection. Should you decide to submit a revised version, I do ask that you get in touch after three months if you have not completed it, to update us on the status.

I look forward to receiving your revised manuscript.

Yours sincerely,

Lise Roth

Lise Roth, PhD
Senior Editor
EMBO Molecular Medicine

EMBO Press participates in many Publish and Read agreements that allow authors to publish Open Access with reduced/no publication charges. Check your eligibility: https://authorservices.wiley.com/author-resources/Journal-Authors/open-access/affiliation-policies-payments/index.html

***** Reviewer's comments *****

Referee #1 (Comments on Novelty/Model System for Author):

In this paper entitled "Single cell profiling of muscle infiltrating T cells in idiopathic inflammatory myopathies", the authors conducted single cell sequencing on T cells infiltrating into muscles and memory T cells in peripheral blood obtained from patients with different subgroups of idiopathic inflammatory myopathies (IIM). They identified distinct T cell signature in muscle-infiltrating T cells such as CXCR4. According to unsupervised clustering, they identified 14 T cell subsets with distinct gene expressions and assumingly different function, including the population expressing tissue resident memory (TRM) receptors infiltrating dominantly in muscles. They showed the presence of They detected clonally expanded T cells in muscle tissue and peripheral blood and showed different T cell subsets in muscles and peripheral blood shared the CDR3 sequencing, implying their interaction or differentiation link. They also showed the expanded T cell clones persisted even after the treatment.
The data are novel and informative and should be of great interest to the researchers in this area.
The manuscript is well written, and results are clearly presented, but there are some points that require more careful examination.

Referee #1 (Remarks for Author):

In this paper entitled "Single cell profiling of muscle infiltrating T cells in idiopathic inflammatory myopathies", the authors conducted single cell sequencing on T cells infiltrating into muscles and memory T cells in peripheral blood obtained from patients with different subgroups of idiopathic inflammatory myopathies (IIM). They identified distinct T cell signature in muscle-infiltrating T cells such as CXCR4. According to unsupervised clustering, they identified 14 T cell subsets with distinct gene expressions and assumingly different function, including the population expressing tissue resident memory (TRM) receptors infiltrating dominantly in muscles. They detected clonally expanded T cells in muscle tissue and peripheral blood and showed different T cell subsets in muscles and peripheral blood shared the CDR3 sequencing, implying their interaction or differentiation link. They also showed the expanded T cell clones persisted even after the treatment.
The data are novel and informative and should be of great interest to the researchers in this area.
The manuscript is well written, and results are clearly presented, but there are some points that require more careful examination. The authors could significantly strengthen the manuscript by addressing the following concerns.

Comments;
#1. As indicated by the fact that the authors could not detect enough T cells from half of the participants, T cells are not always abundantly fond in the muscles in IIM and have not been indicated to be crucial effectors in some of the subtypes of IIM. The reviewer wonders how much the impact of T cells on the pathogenesis of each IIM subset. The results obtained in this research might be limited to the subgroups of patients who has abundant infiltration of T cells in their muscles. It might be good to specify how many of T cells analyzed were derived from each patient and to show the localization of T cells in the muscles derived especially from IMNM patients.

#2. In figure 3d, it is difficult to see if there are CD3+HOBIT+ cells in "6-IBM" patient. In addition, to confirm that the HOBIT+ CD3- nuclei are those of muscle cells, the reviewer thinks it is necessary to identify the nuclei are within their sarcolemma. The authors stated that they did not detect HOBIT-positive T cells in patient 3 and 4, but the reviewer could see CD3+HOBIT+ cells in "3-DM" pictures in Figure 3d.

Referee #2 (Remarks for Author):

The manuscript by Argyriou and colleagues describes the interesting and medically important profiling of T cells in patients afflicted with idiopathic inflammatory myopathy (IMM). In this work, the authors find that T cells isolated from patient muscle exhibit distinct transcriptional profiles than peripheral T cells found in the blood, and that muscle T cell signatures vary between IMM and other myopathies, as well as different forms of IMM can be distinguished by T cell gene expression profiles. The single cell transcriptomics performed to acquire these data are sound, well-described, and appear to conform to the field's quality control and bioinformatics standard practices. Overall, this work has the potential to provide new and powerful insights into the involvement of T cells in IMM.

This reviewer has only one major concern. The muscle and peripheral T cell clusters show profound gene expression differences, however, they are also treated very differently prior to analysis. The possibility that the digestion protocols to isolate T cells from muscle biopsies are the cause of these vast differences should be ruled out prior to publication of these data. Do PB T cells show the same or similar profiles if treated with identical procedures?

Minor concern- There are several typographical and grammatical errors in this initial manuscript. A thorough proofreading is recommended prior to a revision resubmission.

Referee #3 (Comments on Novelty/Model System for Author):

The current major limitation is the lack of adequate controls. From healthy controls for comparisons across the study and also lack of technical controls.

Referee #3 (Remarks for Author):

This study aims to further our understanding of idiopathic inflammatory myopathies focusing on the role of T-cells. The overall idea is of great importance as it aims to shed light on an important clinical need. Clonal expansion analysis is a very interesting approach and analysis of patients at early diagnosis and after treatment is very informative. Nevertheless, there are several areas of concern.
T-cell selection
The choice of markers for tissue T cells and PB memory T-cells need to be explained and justified better. How will the use of different markers between tissue and PB affects the results?
Was the tissue perfused before isolating the T-cells? If not there is an expectation that at least some T-cells will have a common signatures between tissue and PB as the tissue is irrorated by blood.
CD45RA is typically expressed on naïve T-cells, what is the aim of including CD3+ CD45RA+ cells are included?
Indeed, the gating strategy for PB memory T-cells is unusual especially when using an L-shaped gate that includes a multitude of subpopulations such as CD45RA+ /CCR7-, CD45RA-/CCR7+, CD45RA-/CCR7- cells. Further, the gating shown in Fig1A is misleading compared to the gating strategy used in Suppl Fig 2 which also includes CD3.
What isoforms are recognised by the CD45 antibody used for sorting the muscle-resident T cells?

Lack of healthy controls
Comparison of muscle T-cells from IIM patients with additional controls is needed: ideally with T-cells from healthy muscle samples. Although these cells might be rare publicly available datasets suggests they are not impossible to capture (DOI:10.1101/2022.05.24.493094; DOI:10.1186/s13395-020-00236-3; DOI: 10.1038/s41598-019-57110-6; DOI: 10.1038/s41586-020-2797-4). This could be done with single cell transcriptomics approaches or at least for specific targets at the tissue level. The same is valid for circulating T-cells, it would be useful to compare these to healthy controls. The use of controls is crucial to verify that the changes claimed across different IIM types are effectively a common feature of IMMs. Unfortunately based on the low n numbers stratification of different IMMs cannot be performed at this stage but that is understandable. Of note even the validation expt (e.g. Fig 3d are missing healthy controls).

Single-cell transcriptomics analysis
QC steps need to be explained in more detail.
How was the integration performed? Also, please provide evidence of how well data integrated.
Please clarify what this means: 'Ribosomal reads were excluded from the data since they are often technical artifacts or housekeeping transcription activity'. Also, the percentage of ribosomal genes can be used as QC metric, so excluding them does not seem a useful step.
Line 387, do you mean mitochondrial genes?
How was DEG analysis performed and what statistical methods were used?
Fig1d is puzzling, there should be more genes upregulated in memory T cells compared to muscle T cells.
Fig. 2e is hard to follow and match colours. Adding bars on top of the pairs would be clearer.

Others
The authors state that 'using this strategy, we recovered most T cells present in the muscle biopsy', how was this verified?
Negative controls such as FMOs and isotype controls should be shown for FACS experiments.
Fig3d, negative controls such as isotype controls (assuming this panel shows IF) should be shown. Healthy controls should also be included and quantification should be performed.
Methods need to be expanded such as describing the SMARt-seq2 protocol.
Fig3 d, legend should explain what the image represent, is this immunofluorescence staining?

Referee #1 (Comments on Novelty/Model System for Author):

In this paper entitled "Single cell profiling of muscle infiltrating T cells in idiopathic inflammatory myopathies", the authors conducted single cell sequencing on T cells infiltrating into muscles and memory T cells in peripheral blood obtained from patients with different subgroups of idiopathic inflammatory myopathies (IIM). They identified distinct T cell signature in muscle-infiltrating T cells such as CXCR4. According to unsupervised clustering, they identified 14 T cell subsets with distinct gene expressions and assumingly different function, including the population expressing tissue resident memory (TRM) receptors infiltrating dominantly in muscles. They showed the presence of They detected clonally expanded T cells in muscle tissue and peripheral blood and showed different T cell subsets in muscles and peripheral blood shared the CDR3 sequencing, implying their interaction or differentiation link. They also showed the expanded T cell clones persisted even after the treatment.
The data are novel and informative and should be of great interest to the researchers in this area.
The manuscript is well written, and results are clearly presented, but there are some points that require more careful examination.
We thank reviewer 1 for expressing interest in our study and for the constructive feedback.

Comments:
#1. As indicated by the fact that the authors could not detect enough T cells from half of the participants, T cells are not always abundantly fond in the muscles in IIM and have not been indicated to be crucial effectors in some of the subtypes of IIM. The reviewer wonders how much the impact of T cells on the pathogenesis of each IIM subset. The results obtained in this research might be limited to the subgroups of patients who has abundant infiltration of T cells in their muscles. It might be good to specify how many of T cells analyzed were derived from each patient and to show the localization of T cells in the muscles derived especially from IMNM patients.

We thank the reviewer for this comment. Indeed, we observed a correlation between the presence of T-cell infiltrates observed by immunohistochemistry and by flow cytometry (Table EV1). We now provide a summary table indicating the number of T cells which were sorted and analysed for both transcriptomics and CDR3 sequences (Data Set EV9). Indeed, this technology is very well suited for analyzing tissues with abundant immune infiltrates, but it showed to be powerful on the opposite circumstances; we were able to detect T cells from two IMNM cases with scarce infiltrates and in 2 biopsies taken after treatment where immune infiltrates were less apparent by immunohistochemistry (Appendix Figure S1 and Table EV1).
These limitations are now discussed in the Discussion section, page 14, Line 289-292: "*However, the sometimes patchy distribution of inflammatory infiltrates in the muscle tissue, the lack of immune infiltrates, or a predominance of other immune cells such as macrophages might account for the fact that we did not detect T cells by flow cytometry in muscle biopsies from all patients with IIM.*"

We agree that it is particularly intriguing that we could sort T cells from biopsies from patients with IMNM, which are usually characterized by few immune infiltrates[1]. However, CD3+ T cells densities in muscle biopsies from anti-SRP+ and anti-HMGCR+ patients were shown to be the same as in anti-Jo1+ patients[2] in a previous publication, highlighting the possibility to study these cells from muscle biopsies. In our study, small CD3+ clusters were observed in the endomysium and perimysium in one patient (patient 1, IMNM) (**Figure A, below**). In the second patient (patient 2, IMNM), an increased number of CD3+ T cells was detected in the perimysium, but they were mainly scattered. T cell localization in biopsies is now presented in Appendix Figure S1, and a report from an experienced pathologist is summarized in Table EV1. Importantly, in both IMNM patients, clonally expanded CD8+ T

cells were observed in the muscle (revised Figure 4) which, to our knowledge, has never been reported before. These clones persisted after immunosuppressive treatment in one tested patient (patient 2, IMNM) (revised Figure 5).

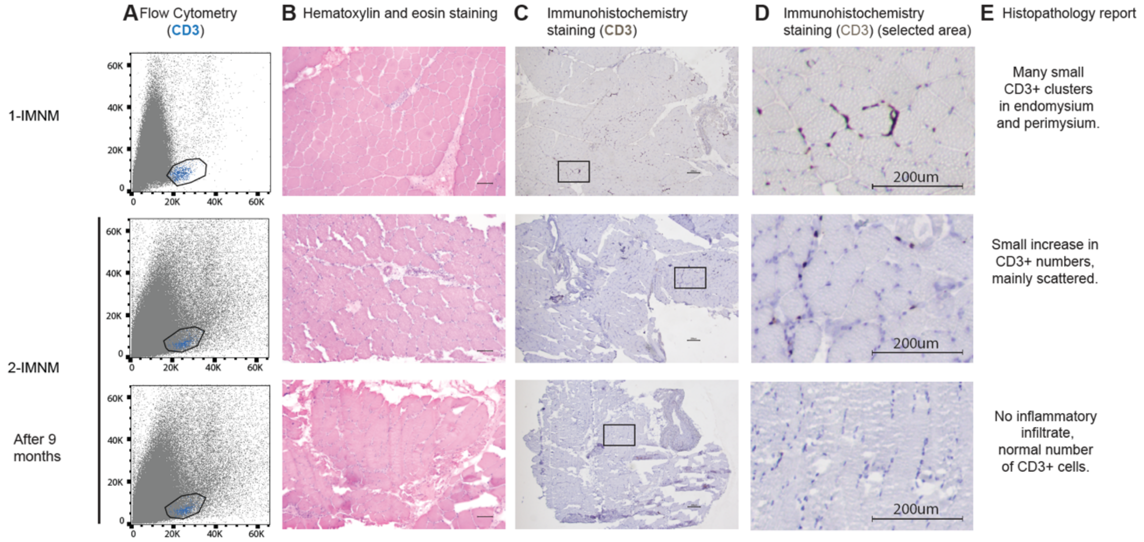

Figure A. **Immune infiltrates in muscle biopsies from two patients with IMNM: Immune-Mediated Necrotizing Myopathy (patient 1-IMNM: upper panel, patient 2-IMNM: middle panel, patient 2-IMNM after 9 months of immunosuppressive treatment: lower panel**). A) Flow cytometry dot plot showing CD45+CD3+ lymphocyte infiltrates (in blue) in muscle biopsies. B) Hematoxylin and eosin staining of muscle biopsies, scale bar=100um. C) Immunohistochemistry staining showing CD3+ lymphocytes (in brown), scale bar=200um. **D**) selected square area from C) showing examples of T-cell infiltrates. E) Histopathology report. SSC: side scatter, FSC: forward side scatter.

#2. In figure 3d, it is difficult to see if there are CD3+HOBIT+ cells in "6-IBM" patient. In addition, to confirm that the HOBIT+ CD3- nuclei are those of muscle cells, the reviewer thinks it is necessary to identify the nuclei are within their sarcolemma.
The authors stated that they did not detect HOBIT-positive T cells in patient 3 and 4, but the reviewer could see CD3+HOBIT+ cells in "3-DM" pictures in Figure 3d.

We thank the reviewer for these comments. We have performed additional immunofluorescence stainings using anti-dystrophin antibodies to stain the sarcolemma (in purple in Figure 3E). We now also provide a larger magnification of a representative HOBIT+ CD3+ staining in a patient with IBM (patient 6) in Figure 3E, left panel (**Figure B, below**). We have also blindly quantified the number of HOBIT+ T cells in muscle sections available from patients 6(IBM), 3(DM), 4(ASyS) and an additional IBM patient (patient 16). We indeed observe HOBIT+ T cells in DM and ASyS but to a lesser extent than in patients with IBM. We observed that 57.4% of T cells were HOBIT+ in muscle biopsies of IBM (patient 6) and 24.6% in IBM (patient 16) whereas this percentage reached 9.8% and 21.7% in DM and ASyS patients, respectively. HOBIT+ T cells were identified as a positive HOBIT staining colocalizing with a nuclear Hoechst staining surrounded by a CD3 staining as described in the method section page 17, Line 365-366.
We have edited the text in the result section on page 8, Lines 126-129, as follows: "*At the protein level, we confirmed the presence of HOBIT-positive T cells among muscle infiltrating T cells using confocal microscopy in patient 6 (IBM) and to a lesser extent in patient 3 (DM) and 4 (ASyS) (Fig. 3E-G)*."

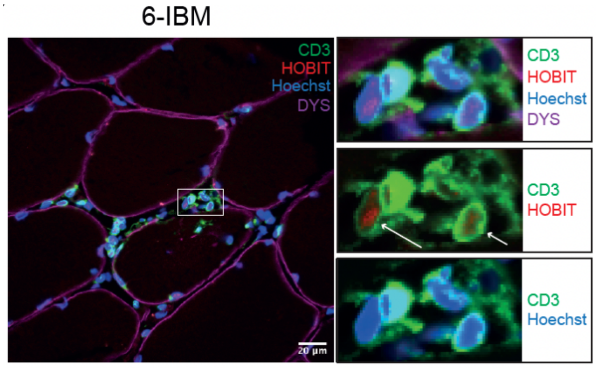

Figure B) Representative immunofluorescence staining of HOBIT+ T cells. HOBIT (red), CD3 (green), dystrophin (purple) and Hoechst 33342 (blue) stainings on a muscle biopsy from patient 6 (IBM). DYS: dystrophin, scale bar 20µm. White arrows indicate HOBIT staining.

We agree that the HOBIT staining in muscle fibers is intriguing. We now show additional representative immunofluorescence and immunohistochemistry HOBIT stainings in muscle cells (Figure EV4 and **Figure C, below)** that demonstrate positive HOBIT staining in nuclei underneath the sarcolemma. Although this is a new finding, it falls beyond the scope of this article and was not further investigated.

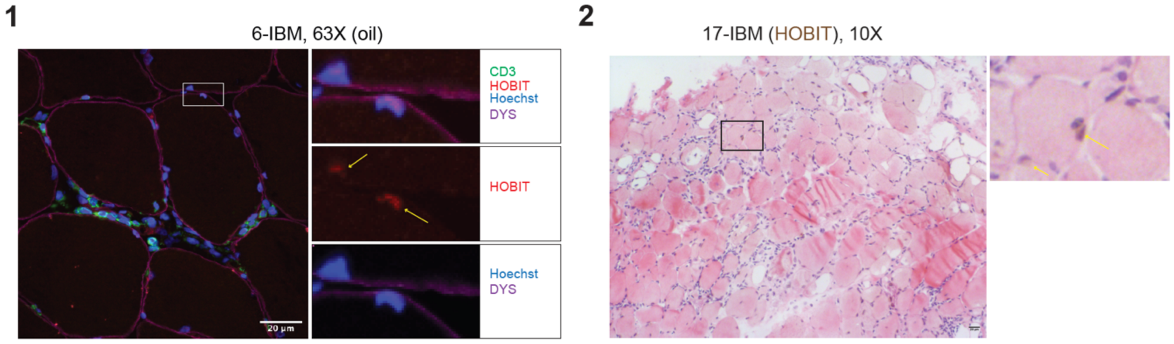

Figure C) Representative staining of HOBIT staining in muscle cells. **1)** Immunofluorescence staining showing HOBIT (red), CD3 (green), dystrophin (purple) and Hoechst 33342 (blue) stainings performed on a muscle biopsy from patient 6 (IBM). DYS: dystrophin, scale bar 20µm. **2)** Immunohistochemistry staining showing HOBIT expression (in brown), scale bar 20µm. 1-2) Yellow arrows indicate HOBIT staining.

Referee #2 (Remarks for Author):

The manuscript by Argyriou and colleagues describes the interesting and medically important profiling of T cells in patients afflicted with idiopathic inflammatory myopathy (IMM). In this work, the authors find that T cells isolated from patient muscle exhibit distinct transcriptional profiles than peripheral T cells found in the blood, and that muscle T cell signatures vary between IMM and other myopathies, as well as different forms of IMM can be distinguished by T cell gene expression profiles. The single cell transcriptomics performed to acquire these data are sound, well-described, and appear to conform to the field's quality control and bioinformatics standard practices. Overall, this work has the potential to provide new and powerful insights into the involvement of T cells in IMM.

We thank reviewer 2 for highlighting the relevance of our study.

#1. This reviewer has only one major concern. The muscle and peripheral T cell clusters show profound gene expression differences, however, they are also treated very differently prior to analysis. The possibility that the digestion protocols to isolate T cells from muscle biopsies are the cause of these vast differences should be ruled out prior to publication of these data. Do PB T cells show the same or similar profiles if treated with identical procedures?

We thank the reviewer for this important comment and question. We agree that the digestion protocol could contribute to some of the transcriptomics differences between the two tissues. Therefore, to address this question, we treated peripheral blood mononuclear cells from healthy donors (n=3) using the same digestion protocol we used for the muscle tissue. We then sorted memory CD3+ T cells and performed Smart-seq3 single cell sequencing (Figure EV3). When comparing the two datasets (non-digested versus digested), we detected significant differences that could account for the observed transcriptomic differences between muscle and PB T cells (**see Figure D below** and Figure EV3).

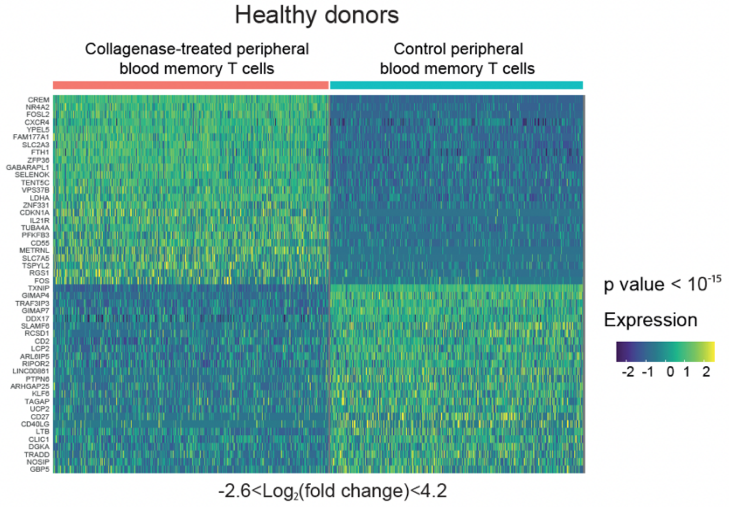

Figure D. Gene expression changes in peripheral blood memory T cells after collagenase treatment. Heatmap showing the normalized and scaled expression of the top 50 differentially upregulated and downregulated genes in healthy control PB memory T cells treated with collagenase pool of n=3 donors.

We have therefore performed a new cluster analysis on muscle and PB T cells independently (revised Figures 1 and 2, and **Figure E below**), confirming that the previously described T-cell subsets are still detected in muscle and blood. Importantly, these new analyses do not affect the previous findings regarding the identification of (i) tissue-resident memory T cells, (ii) expanded T-cell clones in muscle and blood, and (iii) the persistence of T-cell clones after immunosuppressive treatment.

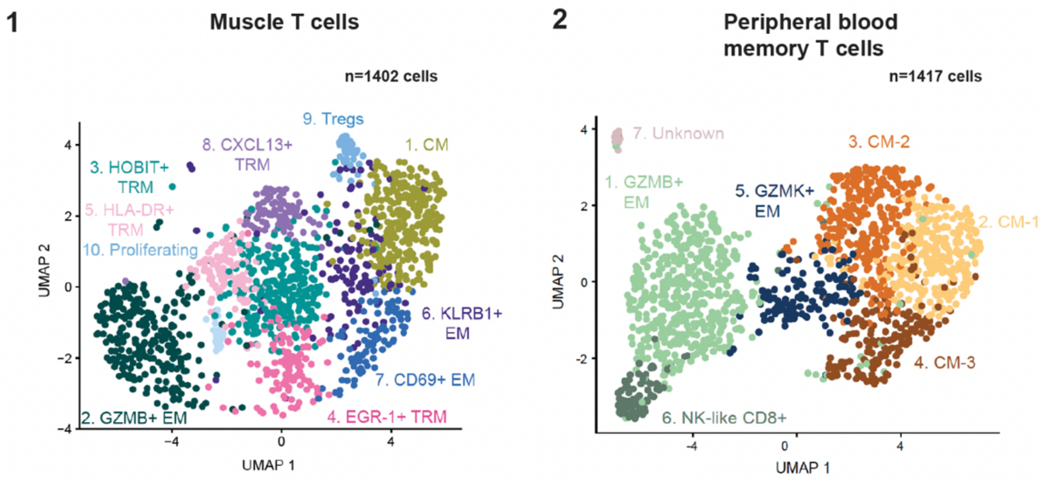

Figure E. T-cell clusters identified in the muscle and peripheral blood of patients with Idiopathic Inflammatory Myopathies (IIM). 1) UMAP displaying 10 T-cell clusters in the muscle of patients with IIM (n=1402 cells). **2**) UMAP displaying 7 T-cell clusters identified in peripheral blood (PB) of patients with IIM (n=1417 cells).

To further compare muscle and blood T cell transcriptomic signatures, we filtered out the genes which expression was affected (negatively or positively) by the collagenase treatment (Fig EV3 G) and **Figure F below**). Using this strategy, we detected a tissue resident memory T-cell signature with *XCL1, XCL2, CXCR6, CRTAM,* and *CXCL13 expression* in T cells in muscle biopsies.

This data is now described in the result section page 7, lines 110-114: "*We then compared DEG between muscle T cells and PB memory T cells from patients with IIM, after removal of all the genes affected by enzymatic extraction (Fig EV3G). In the top 50 upregulated genes after the filtering, XCL1, XCL2[22], CXCR6[19], CRTAM[20], and HOBIT[21,22] associated with TRM T-cell formation in different tissues, were detected in muscle T cells (Fig EV3G and Data Set EV7).*"

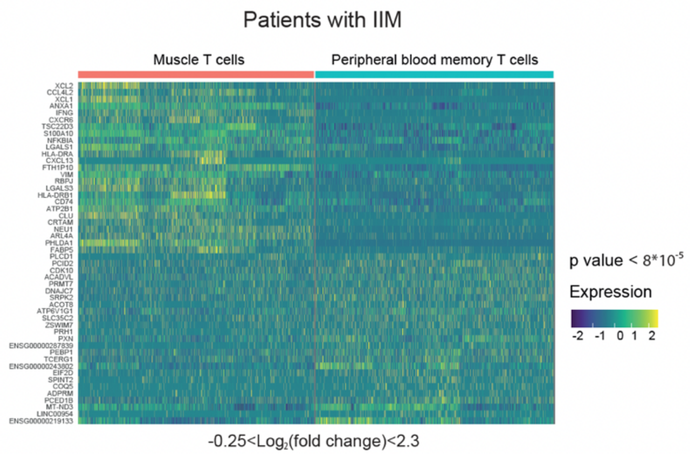

Figure F. Gene signatures in muscle T cells versus peripheral blood memory T cells in patients with idiopathic inflammatory myopathies. Heatmap showing the normalized and scaled expression of the top 50 differentially upregulated and downregulated genes in muscle T cells versus PB memory T cells patients with IIM after filtering out genes shown in Data Set EV6.

#2. Minor concern- There are several typographical and grammatical errors in this initial manuscript. A thorough proofreading is recommended prior to a revision resubmission.

We apologize for these errors. The revised manuscript has been thoroughly assessed by all authors including native north American speaker Annika van Vollenhoven.

Referee #3 (Comments on Novelty/Model System for Author):

The current major limitation is the lack of adequate controls. From healthy controls for comparisons across the study and also lack of technical controls.

Referee #3 (Remarks for Author):

This study aims to further our understanding of idiopathic inflammatory myopathies focusing on the role of T-cells. The overall idea is of great importance as it aims to shed light on an important clinical need. Clonal expansion analysis is a very interesting approach and analysis of patients at early diagnosis and after treatment is very informative. Nevertheless, there are several areas of concern.
We thank reviewer 3 for highlighting the importance of our study.

#1. T-cell selection
The choice of markers for tissue T cells and PB memory T-cells need to be explained and justified better. How will the use of different markers between tissue and PB affects the results?
We thank the reviewer for this comment. CD45 is a tyrosine phosphatase exclusively expressed on hematopoietic cells[3]. The combined use of anti-CD45 and anti-CD3 antibodies allows the enrichment of T cells from the muscle tissue and minimizes the sorting of cells/debris, which could bind not specifically to anti-CD3 antibodies. A similar strategy has been used for instance, to sort CD56+ NK cells from human tissues[4]. We have added one sentence to explain this strategy in the method section on page 17, lines 374-376: "*Anti-CD45 and anti-CD3 antibodies were used to enrich for T cells within the muscle tissue and to minimize sorting of cells/debris which could bind not specifically to anti-CD3 antibodies.*" This strategy was efficient since 95% of the sorted cells were CD3+ T cells based on their gene expression (Data Set EV9). We did not use anti-CD45 antibodies for sorting blood CD3+ T cells because all blood leukocytes are CD45-positive[3] (see **Figure G below**).

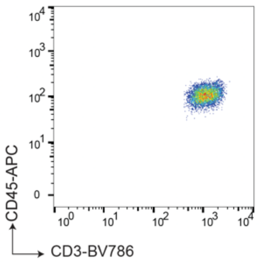

Figure G. Flow cytometry dot plot showing CD45 expression on CD3+ T cells (after gating for lymphocytes/singlets and excluding dead cells)

We chose to sort PB CD3+ memory T cells (by excluding naïve CD45RA+CCR7+ T cells) to maximize the possibility to identify expanded T cell clones and to reduce sequencing costs. This information is available in the material section, page 18, Lines 379-380: "*We sorted memory T cells from PB to maximize the possibility to identify expanded T-cell clones and to reduce sequencing costs.*"

We have now independently clustered muscle and PB T cells (see comment 1 from reviewer 2). We still identify tissue resident memory T cells in the muscle (revised Figure 1 and Figure 3) and expanded T-cell clones in both compartments. By removing naïve T cells from the blood in our gating strategy, we certainly enrich for expanded T-cell clones. In all figures displaying the peripheral blood compartment, we have highlighted that T cells originate from "peripheral blood memory T cells".

#2. Was the tissue perfused before isolating the T-cells? If not there is an expectation that at least some T-cells will have a common signatures between tissue and PB as the tissue is irrorated by blood.

To not affect T cells present in the muscle biopsy, we minimized extra manipulations such as tissue perfusion. We agree with reviewer 3 that we would expect to find few T cells originating from PB within the muscle biopsy. We reasoned that such T cells would have a similar transcriptome profile as T cells from blood, although their transcriptomic signature would be affected by the digestion protocol (see comment 1 from reviewer 2 and Figure EV3 F).

Clustering of PB and muscle T cells in the first submission revealed one cluster originating from muscle T cells with a transcriptome profile similar to blood T cells (cluster 12, see **Figure H.1 below**). When we compared the transcriptomic data from cluster 12 with its blood counterpart (cluster 4), we detected several upregulated genes (*CXCR4, CREM, DUSP2*, etc..), which were also detected in PB T cells from healthy donors treated with collagenase (Figure EV3 F) and **Figure H.2 below**). In the new clustering of muscle T cells **(Figure H.3 below)**, these cells fall within the central memory fraction (**Figure H.4 below**). Hence, it is probable that these few T cells, which correspond to the initial cluster 12 (i.e., same cell IDs) and are now embedded in the new cluster 1, might be blood T cells irrigating the muscle tissue. Still, they do not affect the clustering of muscle T cells. Since these cells represent a minor fraction of the sorted T cells and that we cannot confirm that they come from blood, they were not removed from the analysis.

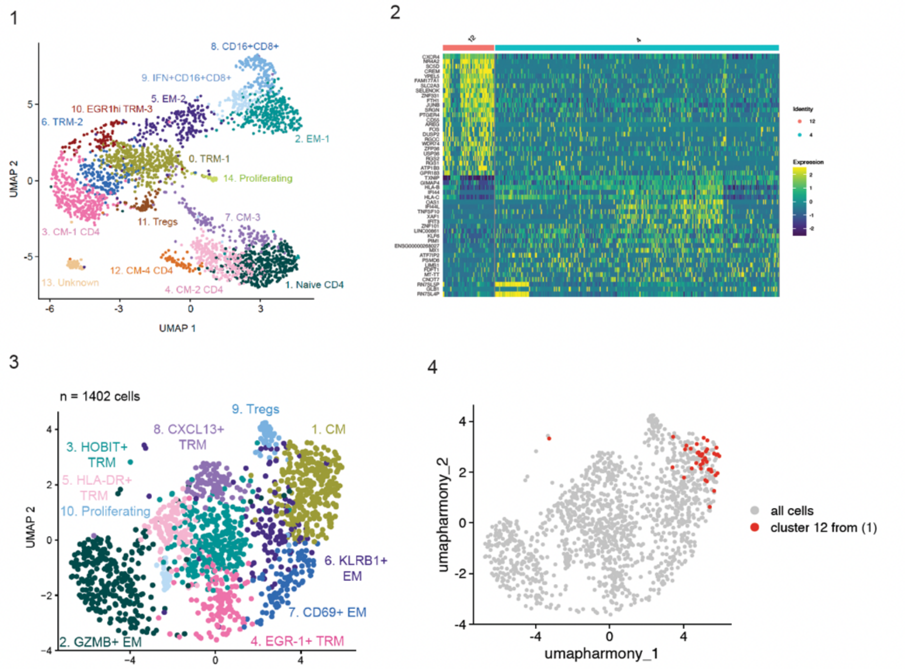

Figure H. Identification of peripheral blood (PB) T cells irrigating the muscle biopsy. **1**) UMAP clustering of peripheral blood memory T cells and muscle T cells from n=7 patients with IIM (first submission). **2**) Heatmap showing the differential expression of genes from cluster 12 (muscle) and cluster 4 (PB) (first submission). **3**) UMAP of muscle T cells from n=7 patients with IIM (revised manuscript). **4**) Cells from cluster 12 in panel 1) belong to the central memory muscle T cell cluster in panel 3) (revised manuscript). IIM: Idiopathic inflammatory myopathy.

#3. CD45RA is typically expressed on naïve T-cells, what is the aim of including CD3+ CD45RA+ cells are included? Indeed, the gating strategy for PB memory T-cells is unusual especially when using an L-shaped gate that includes a multitude of subpopulations such as CD45RA+ /CCR7-, CD45RA- /CCR7+, CD45RA-/CCR7- cells.
Indeed, CD45RA+ is expressed on naïve CD4+ T cells, and the combination of CCR7 and CD45RA antibodies can be used to delineate naïve (CCR7+CD45RA+), central memory (CCR7+CD45RA-), effector memory (CCR7-CD45RA-) and TEMRA (T effector memory re-expressing CD45RA) (CCR7-CD45RA+)[5]. As discussed above, our strategy to exclude CCR7+ CD45RA+ naïve T cells was done to maximize the possibility of detecting expanded T cell clones in PB while minimizing sequencing costs. Certainly, we detected expanded T cell clones in all patients (revised Figure 4).

Further, the gating shown in Fig1A is misleading compared to the gating strategy used in Suppl Fig 2 which also includes CD3.
We agree with the reviewer and we have modified Fig 1A to clarify the gating strategy.

What isoforms are recognised by the CD45 antibody used for sorting the muscle-resident T cells?
The anti-CD45 monoclonal antibody (clone HI30, BD biosciences) binds to the 180 (CD45RO), 190, 205, 220 (CD45RA) kDa protein isoforms of CD45. It will recognize all hematopoietic cells, including naïve (CD45RA) and memory (CD45RO) T cells. We did not include a CCR7/CD45RA staining for muscle T cells because we wanted to capture all possible CD3+ T cells present within the biopsy.

#4. Lack of healthy controls
Comparison of muscle T-cells from IIM patients with additional controls is needed: ideally with T-cells from healthy muscle samples. Although these cells might be rare publicly available datasets suggests they are not impossible to capture (DOI:10.1101/2022.05.24.493094; DOI:10.1186/s13395-020-00236-3; DOI: 10.1038/s41598-019-57110-6; DOI: 10.1038/s41586-020-2797-4). This could be done with single cell transcriptomics approaches or at least for specific targets at the tissue level. The same is valid for circulating T-cells, it would be useful to compare these to healthy controls. The use of controls is crucial to verify that the changes claimed across different IIM types are effectively a common feature of IMMs. Unfortunately based on the low n numbers stratification of different IMMs cannot be performed at this stage but that is understandable. Of note even the validation expt (e.g. Fig 3d are missing healthy controls).

We agree that comparing our datasets with muscle T cells from healthy donors would provide information on tissue resident memory T cells, but it is technically challenging. Muscle biopsies are performed during a surgical procedure, which in the case of our study, were coupled to muscle biopsies for diagnostic purposes. We agree that including biopsies from healthy donors would be important. Still, as these biopsies are small (less than 50 mg) and CD3 positive T cells are very scarce in biopsies from healthy individuals, it is unlikely that we would retrieve enough T cells for sorting. Moreover, in about half of the patients with IIM (Table EV1), in particular patients with dermatomyositis, we could not recover a clear CD45+CD3+ population suggesting that sorting T cells from healthy donors would be equally challenging and would require pooling data from a large number of healthy donors. Finally, we were concerned that blood T cells irrigating the tissue (see comment 2 from reviewer 3) might outnumber the number of few infiltrating T cells in healthy donor´s biopsies. Regardless of these caveats, we agree that such studies could inform about the presence of possible tissue-resident memory T cells in the muscle of healthy donors. However, we have included added data from PB T cells from healthy donors in the revised version of our manuscript

We thank the reviewer for referencing datasets where T cells have been isolated from healthy muscle tissues. We were particularly interested in the dataset described in De Micheli, A. J. et *al*[6]. However, when we assessed the expression level of *CD4, CD8A*, *CD8B* and *ZNF683* per healthy control, we realized that the different levels of transcriptome coverage between the 10X single cell sequencing and the Smart-seq2 platforms[7] make such data integration and comparison difficult (**Figure I below**). Additionally, little clinical data from these samples is available, making it unclear if these muscle biopsies are from "healthy individuals".
For all these reasons, we decided not to include this dataset in our article, but we acknowledge the need to further assess T cells in the muscle of healthy donors in the discussion section on page 14, lines 296-299: "*Another limitation of this study is the lack of comparison with muscle-infiltrating T cells from healthy donor's biopsies. Such experiments, although technically challenging given the low numbers of T cells infiltrating the healthy muscle tissues will inform about possible differences in the profile of TRM cells at the steady state compared to IIM*"

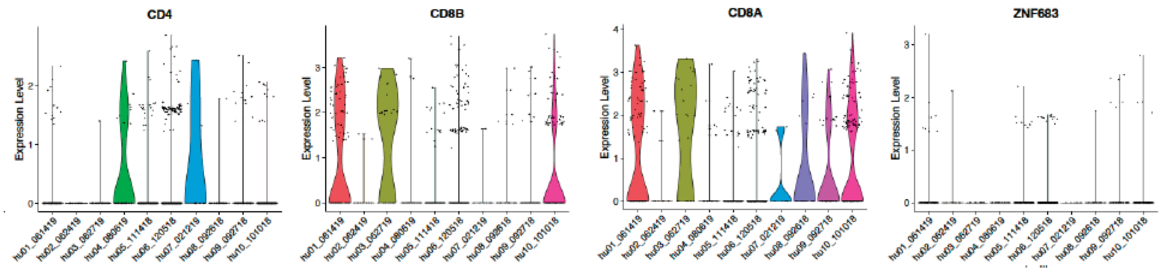

Figure I. T cells in muscle biopsies from healthy donors. CD4, CD8B, CD8A and ZNF683 gene expression levels in T cells from muscle biopsies of healthy donors (n=10 donors) from De Micheli, A. J. et *al*.[6]

To include additional healthy controls to our dataset, we have sorted and single cell sequenced memory T cells from the peripheral blood of three age-matched healthy donors using Smart-seq3 sequencing. Interestingly, a NK-like CD8+ T-cell cluster (revised Figure 2, cluster 6 and **Figure J below**) was detected in memory T cells from patients with myositis and was absent in healthy donors (Fig EV3 B).
The text was revised page 6, lines 96-98: "*The NK-like CD8 T-cell cluster expressing KLRC1, KLRC2, IKZF2 was not identified in Smart-seq3 single cell data of memory T cells from PB of healthy donors (Fig. EV3A-E, Data Set EV5)".*

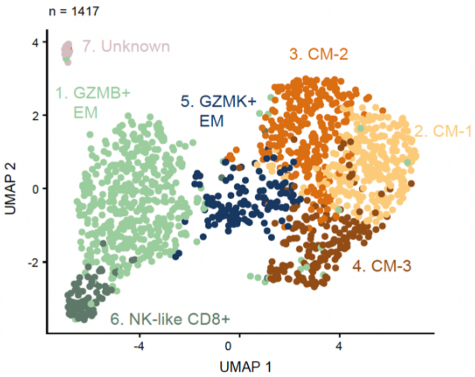

Figure J. T-cell clusters identified in peripheral blood of patients with IIM. UMAP displaying 7 T-cell clusters identified in peripheral blood (PB) of patients with IIM (n=1417 cells) including NK-like CD8+ T cells (cluster 6).

#5. Single-cell transcriptomics analysis
QC steps need to be explained in more detail.
We have edited the sections "data processing and quality control steps"; "cluster analyses"; "T-cell receptor analyses" pages 19-21 and have detailed each QC step.

How was the integration performed? Also, please provide evidence of how well data integrated.

We integrated the data by correcting for sequencing batch effects using the harmony package[8] (version 0.1.0) as described in the method section on page 20, Lines 431-432 as follows: “*Using these principal components, we integrated the data by correcting for sequencing batch effects using harmony package (version 0.1.0)”* and Appendix Figure S3 showing the single cell data analysis pipeline. We show data integration in Figures EV1 (right panels), and EV2 (right panels) in the revised version (**Figure K below).**

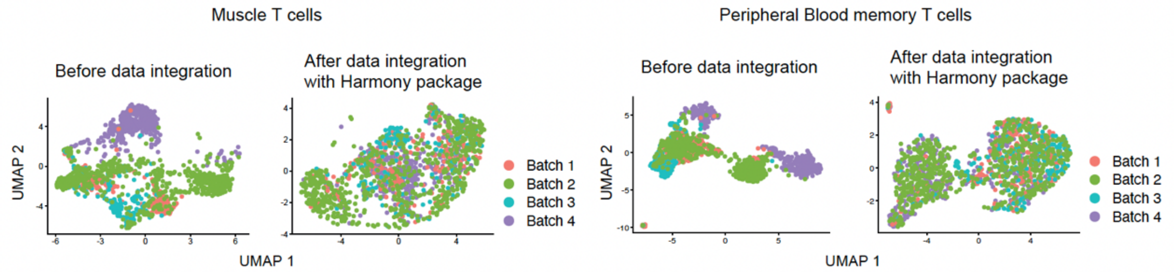

Figure K. Data integration using the Harmony package. UMAP displaying T-cell clusters in muscle (left) and peripheral blood (right) before and after integration using the harmony package. Sequencing batches are highlighted in different colors.

#6.

Please clarify what this means: 'Ribosomal reads were excluded from the data since they are often technical artifacts or housekeeping transcription activity'. Also, the percentage of ribosomal genes can be used as QC metric, so excluding them does not seem a useful step.

Thank you for this comment, and apologies for the confusing description. To clarify our strategy, we have edited the sentence on page 20, lines 425-426 as follows: *“To reduce the technical noise while improving the detection of immune-related genes, we excluded ribosomal genes from the analysis.*”

We estimated that removing ribosomal genes would help reduce the technical noise while improving the detection of immune-related genes. We have therefore excluded genes coding for ribosomal proteins (starting with RPS/RPL) as the expression of these proteins is linked to ribosomal function and the physiological state of the cells[9]. Understanding the contribution of ribosome biogenesis on the different T-cell populations is an important topic, but it is beyond the scope of this study.

Certainly, the percentage of ribosomal genes can be used as a QC metric, particularly in combination with the percentage of mitochondrial genes. **Figure L below** shows the percentage of mitochondrial genes and ribosomal protein genes indicating that we do not have low-quality cells that need to be excluded from further analyses.

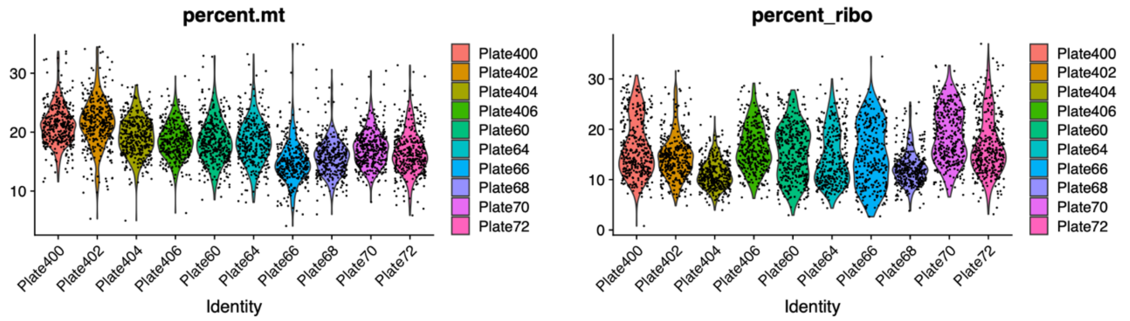

Figure L. Percentage of genes coding for mitochondrial genes (percent.mt) and ribosomal protein coding genes (percent_ribo) per sequencing batch.

#7.
Line 387, do you mean mitochondrial genes?
We apologize for this mistake. It was corrected to "*mitochondrial genes*" on page 19, Line 417.

#8.
How was DEG analysis performed and what statistical methods were used?
We apologize for this omission. This information is now available in the method section on page 20, lines 439-442: "*The differential gene expression was calculated between a given cluster and the rest of the cells using a Wilcoxon Rank Sum test implemented in the FindAllMarkers function from Seurat package, to explore genes that contribute to the cluster formation.*"

#9.
Fig1d is puzzling, there should be more genes upregulated in memory T cells compared to muscle T cells.

Due to a new independent clustering of muscle and PB T cells (see comment 1 from reviewer 2), this figure has been removed.

#10.
Fig. 2e is hard to follow and match colours. Adding bars on top of the pairs would be clearer.
Thank for this comment. Due to a change in the clustering strategy (see comment 1 from reviewer 2), this figure is no longer included in the revised manuscript.

Others
#11.
The authors state that 'using this strategy, we recovered most T cells present in the muscle biopsy', how was this verified?
We apologize if this sentence was unclear. We meant that we sorted all T cells that could be recovered from the biopsy. To avoid confusion, we have removed this sentence.

#12.
Negative controls such as FMOs and isotype controls should be shown for FACS experiments.
We are now including FMO controls in Appendix Figure S2.

#13.
Fig3d, negative controls such as isotype controls (assuming this panel shows IF) should be shown. Healthy controls should also be included and quantification should be performed.
Isotype controls which were initially presented in Supplementary Figure 6c are now included in main **Figure 3F**.
Immunofluorescence stainings were performed on available tissues from patients with IBM (6), DM (3), ASyS (4) and an additional IBM (16). Quantification of HOBIT+ T cells among CD3+ T cells in muscle biopsies was then performed (**Figure 3G**). We unfortunately do not have access to muscle biopsies from healthy donors as indicated in comment 4 from reviewer 3.

#14.
Methods need to be expanded such as describing the SMARt-seq2 protocol.
Smart-seq 2 and Smart-seq3 protocols are now described in detail in Appendix Supplementary Method.

#15.
Fig3 d, legend should explain what the image represent, is this immunofluorescence staining?
We updated the legend as follows: "*Fig 3E) Representative immunofluorescence stainings of HOBIT (red), CD3 (green), dystrophin (purple) and Hoechst 33342 (blue) performed on muscle tissue for patient 6 (IBM), patient 3 (DM) and 4 (ASyS). Images were acquired using a LSM 880 confocal microscope without Airyscan microscope (63x oil objective). Scale bar=20μm.*"

REFERENCES

1. Merlonghi, G., Antonini, G. & Garibaldi, M. Immune-mediated necrotizing myopathy (IMNM): A myopathological challenge. *Autoimmun Rev* **21**, 102993 (2022).
2. Allenbach, Y., *et al.* Necrosis in anti-SRP(+) and anti-HMGCR(+)myopathies: Role of autoantibodies and complement. *Neurology* **90**, e507-e517 (2018).
3. Trowbridge, I.S. & Thomas, M.L. CD45: an emerging role as a protein tyrosine phosphatase required for lymphocyte activation and development. *Annu Rev Immunol* **12**, 85-116 (1994).
4. Dogra, P., *et al.* Tissue Determinants of Human NK Cell Development, Function, and Residence. *Cell* **180**, 749-763 e713 (2020).
5. Sallusto, F., Lenig, D., Forster, R., Lipp, M. & Lanzavecchia, A. Two subsets of memory T lymphocytes with distinct homing potentials and effector functions. *Nature* **401**, 708-712 (1999).
6. De Micheli, A.J., Spector, J.A., Elemento, O. & Cosgrove, B.D. A reference single-cell transcriptomic atlas of human skeletal muscle tissue reveals bifurcated muscle stem cell populations. *Skelet Muscle* **10**, 19 (2020).
7. Nguyen, Q.H., Pervolarakis, N., Nee, K. & Kessenbrock, K. Experimental Considerations for Single-Cell RNA Sequencing Approaches. *Front Cell Dev Biol* **6**, 108 (2018).
8. Korsunsky, I., *et al.* Fast, sensitive and accurate integration of single-cell data with Harmony. *Nat Methods* **16**, 1289-1296 (2019).
9. Xue, S. & Barna, M. Specialized ribosomes: a new frontier in gene regulation and organismal biology. *Nat Rev Mol Cell Biol* **13**, 355-369 (2012).

15th Jun 2023

Dear Dr. Chemin,

Thank you for the submission of your revised manuscript to EMBO Molecular Medicine, and please accept my apologies for the delay in getting back to you as we were waiting for the last referee report. We have now received the reports from the 3 referees. As you will see, they are supportive of publication pending minor revisions, and we will therefore be able to accept your manuscript once the following points will be addressed:

1/ Referees' comments: please address the remaining concerns from referee #3 in writing.

2/ Main manuscript text:
- Please address the queries from our data editors in the related manuscript file ("Data edited MS file"), accept the previous changes and only keep in track changes mode any new modification.
- Please provide up to 5 keywords.
- Materials and methods:
o We note that part of your methods is included in the Appendix file. As we do not have size limitations for methods, we would encourage you to move the Appendix method to the main manuscript text.
o Patients and healthy controls: please include the full statement that informed consent was obtained from all subjects and that the experiments conformed to the principles set out in the WMA Declaration of Helsinki and the Department of Health and Human Services Belmont Report.
- Please correct the order of the different sections to: Material & Methods, Data Availability, Acknowledgments, References, Figure Legends, EV Figure Legends.
- Data Availability Section: Thank you for providing a link to access the datasets, please note that the data must be public before acceptance of the manuscript. Please merge the Code Availability section with the DAS.
- Conflict of interest: should be renamed to "Disclosure Statement and Competing Interests". We updated our journal's competing interests policy in January 2022 and request authors to consider both actual and perceived competing interests. Please review the policy https://www.embopress.org/competing-interests and update your competing interests if necessary.
- Acknowledgements/Funding: Please make sure the information provided in the system matches the acknowledgement section (currently, KI Rheumatology fond, 2020-01378, Science for Life Laboratory, the Knut and Alice Wallenberg Foundation, the National Genomics Infrastructure funded by the Swedish Research Council, Swedish National Infrastructure for Computing (SNIC), 2018-05973 are missing from the submission system).
- The references should be listed in alphabetical order, with 10 authors before et al. We note that you used data citation for regular journals article (instead of primary datasets) which is incorrect ("Data ref: Kumar et al 2017). Please simply remove the "Data ref" and [DATASET] tags and this reference as regular citations/references.

3/ Figures:
- Please provide exact p values in the figure legends (and EV figures), not a range (when applicable).
- Appendix Figure S2: please provide more details about original cell samples for A, B and C.
- In the main manuscript text, figures should be referenced to in chronological order: callout for Fig. 2F should come after Fig. 2D-E; callout for Fig. 5B should come before Fig. 5C; callouts for Fig. EV1C and Fig. EV1D should go right after callout for Fig. EV1B; callout for Fig. EV2E-F should come after Fig. EV2D; callout for Fig. EV3H-I should go after Fig. EV3G. Additionally, callouts are missing for Fig. EV4A,B.
- Table EV1 should be made a Dataset and the legend removed from the manuscript.
- Please separate EV datasets from Source Data: Source Data should be uploaded as 1 file per figure as per the Checklist provided by Hannah. Please contact us if you have any question regarding this aspect.

4/ Thank you for providing The paper explained. I introduced minor changes, please let me know if you agree or amend as you see fit. Please include the TPE in the main manuscript file.

Problem:
Idiopathic inflammatory myopathies (IIM) are rare autoimmune systemic diseases that primarily affect the skeletal muscle. Importantly, T-cell infiltrates are often detected in the muscle of patients with IIM where they are suspected to contribute to tissue damage. However, the mechanisms involved in T-cell infiltration and persistence in the muscle during disease pathogenesis are still unclear. Current treatment approaches are limited, and patients often relapse. A deeper understanding of the pathogenic mechanisms leading to T-cell accumulation in the muscle is needed to envision the development of novel treatment approaches for patients with IIM.

Results:
This study aims to map the immune profile of muscle-infiltrating T cells in patients with IIM using single-cell RNA sequencing. We identified effector, tissue resident, regulatory and proliferating T cells in the muscle of IIM patients. Moreover, T-cell receptor sequencing revealed expanded muscle T cells in the effector memory and tissue resident memory compartments, suggesting

their maintenance in the tissue. Importantly, after conventional treatments, T-cell clones persisted in the muscle of patients where they might contribute to relapses.

Impact:
This study shows that T cells in skeletal muscle of patients with IIM display tissue resident memory features suggesting their maintenance within the tissue and their probable contribution to relapses. The immunoprofiling map of muscle-infiltrating T cells can be used to understand the mechanisms leading to tissue damage and to identify novel therapeutic targets.

5/ Thank you for providing a synopsis. Please upload the text and image separately. The image should be a PNG/JPEG/TIFF file 550 px wide x 300-600 px high, and the text should remain legible.
I slightly modified the text to fit our format, please let me know if you agree with the following or amend as you fit:

T-cell infiltrates in muscle biopsies of patients with idiopathic inflammatory myopathies (IIM) have been described for decades. Using single-cell sequencing, we show that muscle-infiltrating T cells display tissue resident memory features and that they persist in muscle tissue over time.

• Muscle-infiltrating T cells are characterized by an effector, regulatory, proliferating or tissue resident memory (TRM) phenotype.

• Expanded T-cell clones with effector (Granzyme B) and tissue resident memory features (HOBIT, CXCR6) are identified in the muscle biopsies.

• Effector- and TRM clones persist in the muscle tissue after immunosuppressive treatment

• A type 1 interferon signature is detected in T cells in the muscle tissue of patients with dermatomyositis and anti-synthetase syndrome at early diagnosis.

6/ As part of the EMBO Publications transparent editorial process initiative (see our Editorial at http://embomolmed.embopress.org/content/2/9/329), EMBO Molecular Medicine will publish online a Review Process File (RPF) to accompany accepted manuscripts.
This file will be published in conjunction with your paper and will include the anonymous referee reports, your point-by-point response and all pertinent correspondence relating to the manuscript. Let us know whether you agree with the publication of the RPF and as here, if you want to remove or not any figures from it prior to publication.
Please note that the Authors checklist will be published at the end of the RPF.

I look forward to receiving your revised manuscript.

Yours sincerely,

Lise Roth

Lise Roth, PhD
Senior Editor
EMBO Molecular Medicine

The system will prompt you to fill in your funding and payment information. This will allow Wiley to send you a quote for the article processing charge (APC) in case of acceptance. This quote takes into account any reduction or fee waivers that you may be eligible for. Authors do not need to pay any fees before their manuscript is accepted and transferred to our publisher.

***** Reviewer's comments *****

Referee #1 (Remarks for Author):

The authors improved their manuscript according to the reviewer's comments.

Referee #2 (Comments on Novelty/Model System for Author):

In my opinion, this will be a very impactful paper that will be of interest to clinicians and basic scientists involved in the study of inflammatory muscle diseases.

Referee #2 (Remarks for Author):

The authors did a superb job addressing this reviewer's concerns and, in my opinion, the concerns raised by the other reviewers. The new experimental data to control for potential procedural artifacts should be commended. Also, the thorough editing of this revision makes the report more clear to read.

Referee #3 (Remarks for Author):

This study aims to further our understanding of idiopathic inflammatory myopathies focusing on the role of T-cells. The overall idea is of great importance as it aims to shed light on an important clinical need. Clonal expansion analysis is a very interesting approach and analysis of patients at early stage. The authors have improved the manuscript.
The authors have explained better their rationale for the choice of markers, and this should be included clearly in the text.
The authors state that likely blood-derived T cells found in muscle 'were not removed from the analysis'. The reviewer agrees not to remove cells from the analysis and suggests to include Fig H4 in the revised manuscript as supplementary data.
It would have still been better to use the same antibodies as experimental design, changes due to antibody binding are unlikely but by using both this doubt would have been eliminated.
It is important to include the details about the isoforms of antibodies used in the text. Although the authors replied satisfactorily to the question it is unclear if they added this info in the manuscript.

Referee #1 (Remarks for Author):

The authors improved their manuscript according to the reviewer's comments.
We thank reviewer 1 for the time spent to evaluate our study.

Referee #2 (Comments on Novelty/Model System for Author):

In my opinion, this will be a very impactful paper that will be of interest to clinicians and basic scientists involved in the study of inflammatory muscle diseases.

Referee #2 (Remarks for Author):

The authors did a superb job addressing this reviewer's concerns and, in my opinion, the concerns raised by the other reviewers. The new experimental data to control for potential procedural artifacts should be commended. Also, the thorough editing of this revision makes the report more clear to read.
We thank reviewer 2 for the time spent to evaluate our study and for the positive comments regarding our revision work.

Referee #3 (Remarks for Author):

This study aims to further our understanding of idiopathic inflammatory myopathies focusing on the role of T-cells. The overall idea is of great importance as it aims to shed light on an important clinical need. Clonal expansion analysis is a very interesting approach and analysis of patients at early stage.
The authors have improved the manuscript.
The authors have explained better their rationale for the choice of markers, and this should be included clearly in the text.

We thank reviewer 3 for the time spent to evaluate our study and for acknowledging the clinical importance of our study.
We have indeed included the rationale for the choice of antibodies for the cell sorting strategy in the material and methods section, page 19-20, as follows: "
"Anti-CD45 and anti-CD3 antibodies were used to enrich for T cells within the muscle tissue and to minimize sorting of cells/debris which could bind not specifically to anti-CD3 antibodies" and
"We sorted memory T cells from PB to maximize the possibility to identify expanded T cell clones and to reduce sequencing costs"

The authors state that likely blood-derived T cells found in muscle 'were not removed from the analysis'. The reviewer agrees not to remove cells from the analysis and suggests to include Fig H4 in the revised manuscript as supplementary data.
Although we think it is an interesting point to identify blood T cells irrigating the muscle, it falls outside the scope of the current study, and we are hesitant to include this data as supplementary data.

To be able to fully explain the rationale for this analysis, it would require reintegrating the clustering from the first submitted version (PB+ muscle T cells) and to describe these clusters in detail. We are

afraid that it would confuse the reader. We have therefore decided not to include this piece of data in the revised manuscript.

It would have still been better to use the same antibodies as experimental design, changes due to antibody binding are unlikely but by using both this doubt would have been eliminated.
It is important to include the details about the isoforms of antibodies used in the text. Although the authors replied satisfactorily to the question it is unclear if they added this info in the manuscript.

We have included this information in the following sentence page 20: “Of note, the anti-CD45 monoclonal antibody (clone HI30, BD biosciences) binds to the 180 (CD45RO), 190, 205, 220 (CD45RA) kDa protein isoforms of CD45.”

10th Jul 2023

Dear Dr. Chemin,

Thank you for sending the revised files. I am pleased to inform you that your manuscript is accepted for publication and is now being sent to our publisher to be included in the next available issue of EMBO Molecular Medicine!

Please read below for additional IMPORTANT information regarding your article, its publication and the production process.

Congratulations on your interesting work!

With kind regards,

Lise Roth

Lise Roth, Ph.D
Senior Editor
EMBO Molecular Medicine

Follow us on Twitter @EmboMolMed
Sign up for eTOCs at embopress.org/alertsfeeds

*** *** *** IMPORTANT INFORMATION *** *** ***

SPEED OF PUBLICATION
The journal aims for rapid publication of papers, using using the advance online publication "Early View" to expedite the process: A properly copy-edited and formatted version will be published as "Early View" after the proofs have been corrected. Please help the Editors and publisher avoid delays by providing e-mail address(es), telephone and fax numbers at which author(s) can be contacted.

Should you be planning a Press Release on your article, please get in contact with embomolmed@wiley.com as early as possible, in order to coordinate publication and release dates.

LICENSE AND PAYMENT:

All articles published in EMBO Molecular Medicine are fully open access: immediately and freely available to read, download and share.

EMBO Molecular Medicine charges an article processing charge (APC) to cover the publication costs. You, as the corresponding author for this manuscript, should have already received a quote with the article processing fee separately. Please let us know in case this quote has not been received.

Once your article is at Wiley for editorial production you will receive an email from Wiley's Author Services system, which will ask you to log in and will present you with the publication license form for completion. Within the same system the publication fee can be paid by credit card, an invoice, pro forma invoice or purchase order can be requested.

Payment of the publication charge and the signed Open Access Agreement form must be received before the article can be published online.

PROOFS

You will receive the proofs by e-mail approximately 2 weeks after all relevant files have been sent o our Production Office. Please return them within 48 hours and if there should be any problems, please contact the production office at embopressproduction@wiley.com.

Please inform us if there is likely to be any difficulty in reaching you at the above address at that time. Failure to meet our

deadlines may result in a delay of publication.

All further communications concerning your paper proofs should quote reference number EMM-2022-17240-V3 and be directed to the production office at embopressproduction@wiley.com.

EMBO Press Author Checklist

Corresponding Author Name: Karine Chemin	
Journal Submitted to: Embo Molecular medicine	
Manuscript Number: EMM-2022-17240	

USEFUL LINKS FOR COMPLETING THIS FORM
The EMBO Journal - Author Guidelines
EMBO Reports - Author Guidelines
Molecular Systems Biology - Author Guidelines
EMBO Molecular Medicine - Author Guidelines

Reporting Checklist for Life Science Articles (updated January
This checklist is adapted from Materials Design Analysis Reporting (MDAR) Checklist for Authors. MDAR establishes a minimum set of requirements in transparent reporting in the life sciences (see Statement of Task: 10.31222/osf.io/9sm4x). Please follow the journal's guidelines in preparing your
Please note that a copy of this checklist will be published alongside your article.

Abridged guidelines for figures
1. Data
The data shown in figures should satisfy the following conditions:
- the data were obtained and processed according to the field's best practice and are presented to reflect the results of the experiments in an accurate and unbiased manner.
- ideally, figure panels should include only measurements that are directly comparable to each other and obtained with the same assay.
- plots include clearly labeled error bars for independent experiments and sample sizes. Unless justified, error bars should not be shown for technical
- if n<5, the individual data points from each experiment should be plotted. Any statistical test employed should be justified.
- Source Data should be included to report the data underlying figures according to the guidelines set out in the authorship guidelines on Data

2. Captions
Each figure caption should contain the following information, for each panel where they are relevant:
- a specification of the experimental system investigated (eg cell line, species name).
- the assay(s) and method(s) used to carry out the reported observations and measurements.
- an explicit mention of the biological and chemical entity(ies) that are being measured.
- an explicit mention of the biological and chemical entity(ies) that are altered/varied/perturbed in a controlled manner.
- the exact sample size (n) for each experimental group/condition, given as a number, not a range;
- a description of the sample collection allowing the reader to understand whether the samples represent technical or biological replicates (including how many animals, litters, cultures, etc.).
- a statement of how many times the experiment shown was independently replicated in the laboratory.
- definitions of statistical methods and measures:
  - common tests, such as t-test (please specify whether paired vs. unpaired), simple χ2 tests, Wilcoxon and Mann-Whitney tests, can be unambiguously identified by name only, but more complex techniques should be described in the methods section;
  - are tests one-sided or two-sided?
  - are there adjustments for multiple comparisons?
  - exact statistical test results, e.g., P values = x but not P values < x;
  - definition of 'center values' as median or average;
  - definition of error bars as s.d. or s.e.m.

Please complete ALL of the questions below.
Select "Not Applicable" only when the requested information is not relevant for your study.

Materials

Newly Created Materials	Information included in the manuscript?	In which section is the information available? (Reagents and Tools Table, Materials and Methods, Figures, Data Availability Section)
New materials and reagents need to be available; do any restrictions apply?	Not Applicable	

Antibodies	Information included in the manuscript?	In which section is the information available? (Reagents and Tools Table, Materials and Methods, Figures, Data Availability Section)
For **antibodies** provide the following information:<br>- Commercial antibodies: RRID (if possible) or supplier name, catalogue number and or/clone number<br>- Non-commercial: RRID or citation	Yes	Materials and Methods section and Appendix Table S1

DNA and RNA sequences	Information included in the manuscript?	In which section is the information available? (Reagents and Tools Table, Materials and Methods, Figures, Data Availability Section)
**Short novel DNA or RNA including primers, probes**: provide the sequences.	Not Applicable	

Cell materials	Information included in the manuscript?	In which section is the information available? (Reagents and Tools Table, Materials and Methods, Figures, Data Availability Section)
**Cell lines:** Provide species information, strain. Provide accession number in repository **OR** supplier name, catalog number, clone number, and/**OR** RRID.	Not Applicable	
**Primary cultures:** Provide species, strain, sex of origin, genetic modification status.	Not Applicable	
Report if the cell lines were recently **authenticated** (e.g., by STR profiling) and tested for mycoplasma contamination.	Not Applicable	

Experimental animals	Information included in the manuscript?	In which section is the information available? (Reagents and Tools Table, Materials and Methods, Figures, Data Availability Section)
**Laboratory animals or Model organisms:** Provide species, strain, sex, age, genetic modification status. Provide accession number in repository **OR** supplier name, catalog number, clone number, **OR** RRID.	Not Applicable	
**Animal observed in or captured from the field:** Provide species, sex, and age where possible.	Not Applicable	
Please detail **housing and husbandry conditions**.	Not Applicable	

Plants and microbes	Information included in the manuscript?	In which section is the information available? (Reagents and Tools Table, Materials and Methods, Figures, Data Availability Section)
**Plants:** provide species and strain, ecotype and cultivar where relevant, unique accession number if available, and source (including location for collected wild specimens).	Not Applicable	
**Microbes:** provide species and strain, unique accession number if available, and source.	Not Applicable	

Human research participants	Information included in the manuscript?	In which section is the information available? (Reagents and Tools Table, Materials and Methods, Figures, Data Availability Section)
If collected and within the bounds of privacy constraints report on age, sex and gender or ethnicity for all study participants.	Yes	Materials and Methods section and Table EV1

Core facilities	Information included in the manuscript?	In which section is the information available? (Reagents and Tools Table, Materials and Methods, Figures, Data Availability Section)
If your work benefited from core facilities, was their service mentioned in the acknowledgments section?	Yes	Materials and Methods and acknoledgment sections

Design

Study protocol	Information included in the manuscript?	In which section is the information available? (Reagents and Tools Table, Materials and Methods, Figures, Data Availability Section)
If study protocol has been **pre-registered, provide DOI in the manuscript**. For clinical trials, provide the trial registration number **OR** cite DOI.	Not Applicable	
Report the **clinical trial registration number** (at ClinicalTrials.gov or equivalent), where applicable.	Not Applicable	

Laboratory protocol	Information included in the manuscript?	In which section is the information available? (Reagents and Tools Table, Materials and Methods, Figures, Data Availability Section)
Provide DOI OR other citation details if **external detailed step-by-step protocols** are available.	Yes	Materials and Methods section and Appendix Supplementary Methods

Experimental study design and statistics	Information included in the manuscript?	In which section is the information available? (Reagents and Tools Table, Materials and Methods, Figures, Data Availability Section)
Include a statement about **sample size** estimate even if no statistical methods were used.	Yes	In Materials and Methods section
Were any steps taken to minimize the effects of subjective bias when allocating animals/samples to treatment (e.g. **randomization procedure**)? If yes, have they been described?	Not Applicable	In Materials and Methods section
Include a statement about **blinding** even if no blinding was done.	Yes	In Materials and Methods section
Describe **inclusion/exclusion criteria** if samples or animals were excluded from the analysis. Were the criteria pre-established?<br><br>If sample or data points were omitted from analysis, report if this was due to attrition or intentional exclusion and provide justification.	Yes	In Materials and Methods section
For every figure, are **statistical tests** justified as appropriate? Do the data meet the assumptions of the tests (e.g., normal distribution)? Describe any methods used to assess it. Is there an estimate of variation within each group of data? Is the variance similar between the groups that are being statistically compared?	Yes	In Materials and Methods section

Sample definition and in-laboratory replication	Information included in the manuscript?	In which section is the information available? (Reagents and Tools Table, Materials and Methods, Figures, Data Availability Section)
In the figure legends: state number of times the experiment was **replicated** in laboratory.	Yes	
In the figure legends: define whether data describe **technical or biological replicates**.	Not Applicable	

Ethics

Ethics	Information included in the manuscript?	In which section is the information available? (Reagents and Tools Table, Materials and Methods, Figures, Data Availability Section)
Studies involving **human participants**: State details of **authority granting ethics approval** (IRB or equivalent committee(s), provide reference number for approval.	Yes	In Materials and Methods section
Studies involving **human participants**: Include a statement confirming that **informed consent** was obtained from all subjects and that the experiments conformed to the principles set out in the WMA Declaration of Helsinki and the Department of Health and Human Services Belmont Report.	Yes	In Materials and Methods section
Studies involving **human participants: For** publication of **patient photos**, include a statement confirming that consent to publish was obtained.	Not Applicable	
Studies involving experimental **animals**: State details of **authority granting ethics approval** (IRB or equivalent committee(s), provide reference number for approval. Include a statement of compliance with ethical regulations.	Not Applicable	
Studies involving **specimen and field samples:** State if relevant **permits** obtained, provide details of authority approving study; if none were required, explain why.	Not Applicable	

Dual Use Research of Concern (DURC)	Information included in the manuscript?	In which section is the information available? (Reagents and Tools Table, Materials and Methods, Figures, Data Availability Section)
Could your study fall under dual use research restrictions? Please check biosecurity documents and list of **select agents and toxins** (CDC): https://www.selectagents.gov/sat/list.htm	Not Applicable	
If you used a select agent, is the security level of the lab appropriate and reported in the manuscript?	Not Applicable	
If a study is subject to dual use research of concern regulations, is the name of the **authority granting approval and reference number** for the regulatory approval provided in the manuscript?	Not Applicable	

Reporting

The MDAR framework recommends adoption of discipline-specific guidelines, established and endorsed through community initiatives. Journals have their own policy about requiring specific guidelines and recommendations to complement MDAR.

Adherence to community standards	Information included in the manuscript?	In which section is the information available? (Reagents and Tools Table, Materials and Methods, Figures, Data Availability Section)
State if relevant guidelines or checklists (e.g., **ICMJE, MIBBI, ARRIVE, PRISMA**) have been followed or provided.	Not Applicable	
For **tumor marker prognostic studies,** we recommend that you follow the **REMARK** reporting guidelines (see link list at top right). See author guidelines, under 'Reporting Guidelines'. Please confirm you have followed these guidelines.	Not Applicable	
For **phase II and III randomized controlled trials**, please refer to the **CONSORT** flow diagram (see link list at top right) and submit the CONSORT checklist (see link list at top right) with your submission. See author guidelines, under 'Reporting Guidelines'. Please confirm you have submitted this list.	Not Applicable	

Data Availability

Data availability	Information included in the manuscript?	In which section is the information available? (Reagents and Tools Table, Materials and Methods, Figures, Data Availability Section)
Have **primary datasets** been deposited according to the journal's guidelines (see 'Data Deposition' section) and the respective accession numbers provided in the Data Availability Section?	Yes	In Materials and Methods section
Were **human clinical and genomic datasets** deposited in a public access-controlled repository in accordance to ethical obligations to the patients and to the applicable consent agreement?	Not Applicable	
Are **computational models** that are central and integral to a study available without restrictions in a machine-readable form? Were the relevant accession numbers or links provided?	Yes	In Materials and Methods section. "All scripts used for data analysis are available from GitHub, (https://github.com/scReumaKI/Myositis_scPipeline_2022)"
If publicly available data were reused, provide the respective **data citations in the reference list**.	Yes	In Materials and Methods section: "Tissue resident ...obtained from available datasets in the Gene Expression Omnibus (GEO) with the identifier GSE94964 (31)"
